# Conservation of chromatin conformation in carnivores

**DOI:** 10.1073/pnas.2120555119

**Published:** 2022-02-25

**Authors:** Marco Corbo, Joana Damas, Madeline G. Bursell, Harris A. Lewin

**Affiliations:** ^a^The Genome Center, University of California, Davis, CA 95616;; ^b^Department of Plant and Wildlife Sciences, Brigham Young University, Provo, UT 84602;; ^c^Data Science Lab, Office of the Chief Information Officer, Smithsonian Institution, Washington, DC 20002;; ^d^Department of Evolution and Ecology, University of California, Davis, CA 95616;; ^e^John Muir Institute for the Environment, University of California, Davis, CA 95616

**Keywords:** chromatin conformation, chromosome evolution, carnivores, mammals

## Abstract

High throughput chromatin conformation capture (Hi-C) of leukocyte DNA was used to investigate the evolutionary stability of chromatin conformation at the chromosomal level in 11 species from three carnivore families: Felidae, Canidae, and Ursidae. Chromosome-scale scaffolds (C-scaffolds) of each species were initially used for whole-genome alignment to a reference genome within each family. This approach established putative orthologous relationships between C-scaffolds among the different species. Hi-C contact maps for all C-scaffolds were then visually compared and found to be distinct for a given reference chromosome or C-scaffold within a species and indistinguishable for orthologous C-scaffolds having a 1:1 relationship within a family. The visual patterns within families were strongly supported by eigenvectors from the Hi-C contact maps. Analysis of Hi-C contact maps and eigenvectors across the three carnivore families revealed that most cross-family orthologous subchromosomal fragments have a conserved three-dimensional (3D) chromatin structure and thus have been under strong evolutionary constraint for ∼54 My of carnivore evolution. The most pronounced differences in chromatin conformation were observed for the X chromosome and the red fox genome, whose chromosomes have undergone extensive rearrangements relative to other canids. We also demonstrate that Hi-C contact map pattern analysis can be used to accurately identify orthologous relationships between C-scaffolds and chromosomes, a method we termed “3D comparative scaffotyping.” This method provides a powerful means for estimating karyotypes in de novo sequenced species that have unknown karyotype and no physical mapping information.

## Significance

We found the three-dimensional (3D) structure of chromatin at the chromosome level to be highly conserved for more than 50 million y of carnivore evolution. Intrachromosomal contacts were maintained even after chromosome rearrangements within carnivore lineages, demonstrating that the maintenance of 3D chromatin architecture is essential for conserved genome functions. These discoveries enabled the identification of orthologous chromosomal DNA segments among related species, a method we call 3D comparative scaffotyping. The method has application for putative chromosome assignment of chromosome-scale DNA sequence scaffolds produced by de novo genome sequencing. Broadly applied to biodiversity genome sequencing efforts, the approach can reduce costs associated with karyotyping and the physical mapping of DNA segments to chromosomes.

DNA interactions are responsible for chromatin folding and the genome’s three-dimensional (3D) organization in the cell nucleus during interphase (1). Sequencing technologies, such as chromosome conformation capture (e.g., Hi-C) (2, 3) and chromatin interaction analysis by paired-end tag sequencing (4), revealed the spatial conformation of chromatin within the nucleus and demonstrated that it is organized hierarchically in chromosome territories (1), chromosome compartments (3), topological associated domains (TADs) (5), and DNA loops (6). TADs act as the fundamental unit in which genes and regulatory elements interact (5), and DNA loops play an important role in transcription by bringing physically distant genomic regions into proximity (6). For example, the conserved CCCTC-binding factor (CTCF), known to colocalize with cohesins, creates a structural anchor for the spatial organization of constitutively expressed genes and RNA polymerase II interactions (6). Recent work demonstrated that interphase 3D genome organization in eukaryotes is correlated with the presence or absence of condensin II subunits, and that the presence of condensin II promotes the clustering of centromeres at nucleoli in the nucleus of human cells (7).

Relatively few studies have examined the conservation of 3D chromatin conformation in different species and its role in genome evolution. Mouse and human TADs colocated when compared within the same shared syntenic fragment (5, 8), and CTCF sites were found enriched at the edges of TADs, although these sites were not conserved between species (9). Regions in the human and gibbon genomes where synteny was interrupted by chromosome rearrangements colocalized with TAD boundaries, which was suggested to relate to higher chromatin fragility in these regions (10, 11). A small fraction of these rearrangements was found to destroy or create novel TADs (10, 11). These observations suggest that disruption of TADs could result in functional differences between species by creating new gene-enhancer interactions that may be favored by selection. Additionally, correlations between A/B compartments, specific histone modifications, and replication timing patterns were found within primates (12). These findings suggest that 3D chromosome conformation is conserved at a relatively small scale across species and that it plays an essential role in genome stability, gene expression, and chromatin maintenance. Recently, chromosome fusions in mice were found to change chromosome 3D structure, affecting recombination in the germline (13).

The relative simplicity of Hi-C and related methods has enabled their use as a scaffolding tool for assembling genomes de novo (14). The properties of Hi-C that allow for the identification of contact points within and between chromosomes can be exploited for linking DNA sequence contigs on the same chromosome, even across large physical distances and difficult to assemble regions (15). Large-scale projects, such as the Vertebrate Genomes Project (16), have used Hi-C in conjunction with long DNA sequence reads and other methods to consistently produce high-quality chromosome-scale assemblies (15–18). Combining just two methods, Hi-C and PacBio HiFi reads, produced de novo assemblies with high base call accuracy and chromosome-scale scaffolds (C-scaffold), thus transforming efforts to produce reference-quality genome sequences across the eukaryotic tree of life (19). Recent efforts by the DNA Zoo Consortium (15) to assemble and upgrade large numbers of genome assemblies to chromosome-scale using Hi-C have led to valuable new datasets for comparative genomics and the study of chromosome evolution (20, 21). An important property of these datasets is that haploid chromosome numbers can be estimated from the number of C-scaffolds obtained from Hi-C–based assemblies even when the karyotype is unknown (15, 22). However, Hi-C data have not been used systematically to address the issue of chromosome orthology and evolution. Implementation of a rules-based system for naming and relating C-scaffolds in mammals and possibly other eukaryotic taxa would greatly facilitate comparative genomic analysis. Furthermore, a system for naming or relating C-scaffolds to a reference genome will solve problems in the naming of chromosomes now encountered by large-scale genome sequencing projects (23).

The availability of DNA Zoo’s uniformly produced Hi-C datasets for a large number of vertebrate species creates an unprecedented opportunity to examine the evolution of 3D chromatin conformation at chromosome scale. In this study, we use DNA Zoo Hi-C data to compare 3D chromatin conformation for 11 species in 3 families within the order Carnivora (Felidae, Canidae, and Ursidae). Complete orthologous chromosomes and C-scaffolds were readily identified from the Hi-C contact map patterns, providing a highly accurate method for chromosome identification. The 3D chromatin conformation of chromosomes and subchromosomal fragments was found to be highly conserved within and between the three families of carnivores.

## Results

### Chromosome-Level Conservation of Chromatin Conformation in Felidae.

**Fig. 1.**
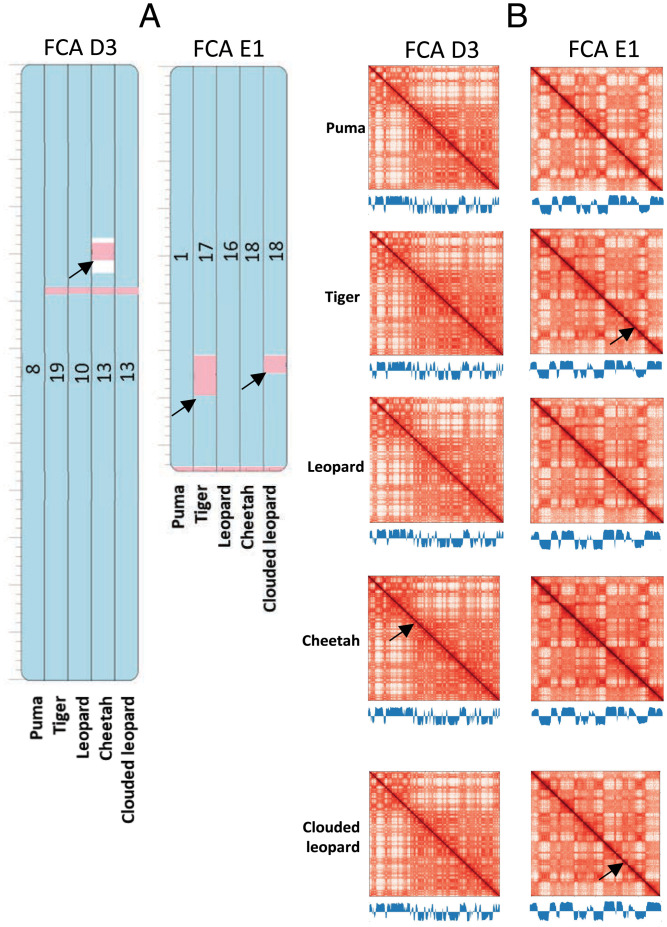
Comparative analysis of chromatin conformation in felids. Puma, tiger, leopard, cheetah, and clouded leopard C-scaffolds orthologous to cat chromosome D3 (FCA D3) and E1 (FCA E1) are shown as examples. (*A*) Homologous synteny blocks of the five felids visualized in Evolution Highway at 300-kb resolution. Blue indicates same sequence orientation as the reference genome. Pink depicts chromosome inversions (arrows indicate inversions bigger than 1 Mb). Numbers represent the scaffold identifier of the target species. (*B*) Juicer plots of orthologous C-scaffolds for the five felids as numbered in part A. Color intensity reflects the frequency of interactions between pairs of loci on C-scaffolds (range 1 to 1,000 for each map). Blue histograms depict eigenvector values for each species matrix at 500-kb resolution. Similar comparisons for all other cat chromosomes are shown in *SI Appendix*, Fig. S2. Alignment coordinates can be found in Dataset S3.

Genome assemblies of six species of felids—clouded leopard, leopard, tiger, cheetah, cat, and puma—were used to investigate whether orthologous chromosomes could be identified from Hi-C contact map patterns. Felid species were selected for analysis first among the carnivores because they have the same diploid number (2*n* = 38) (*SI Appendix*, Fig. S1) (24) and relatively few rearrangements, which simplified the comparative analysis of Hi-C data. Except for the domestic cat, only those felids for which C-scaffold assemblies were available from the DNA Zoo were used to ensure uniformity with respect to tissue source (leukocyte DNA), and methods for library construction, sequencing, and genome assembly (Dataset S1). The cat genome (felCat9) was used as a reference because most sequence scaffolds have been physically mapped to chromosomes (25). However, cat leukocyte Hi-C data were not available from the DNA Zoo collection, which precluded us from including the cat in comparing Hi-C contact maps. Chromosome orthologies were identified by pairwise whole-genome alignments to the cat genome at 300-kb resolution and by visualizing the alignments on cat chromosomes using the Evolution Highway comparative chromosome browser (Fig. 1 and *SI Appendix*, Fig. S2). Each of the 18 cat autosomes and X chromosome corresponds to a single C-scaffold in the other felid species (Fig. 1 and *SI Appendix*, Fig. S2). The Y chromosome was not included in our analysis because only two representative individuals were males (tiger and leopard) (Dataset S1). Very few chromosome rearrangements (only inversions) relative to the cat were identified from the alignments (*n* = 30), most of them being shorter than 1 Mb (*n* = 18) (Fig. 1*A*, *SI Appendix*, Fig. S2, and Datasets S2 and S3).

Hi-C contact maps generated using Juicer were visually inspected for each C-scaffold in the five felid species to determine whether orthologous chromosomes could be recognized using their Hi-C contact patterns. Orthologs of each of the 18 cat autosomes and X were readily identified from their visually distinct Hi-C contact map patterns in all five felid species (Fig. 1*A*, *SI Appendix*, Fig. S2, and Datasets S2 and S3). The autosomal patterns were consistent, adjusting visually for read counts (color intensity), even for chromosomes bearing inversions, such as tiger C-scaffold 17 (Fig. 1). The X chromosome showed variation in Hi-C contact map patterns and intensity, primarily due to differences in the number of reads mapped for clouded leopard, puma, and cheetah, as expected for homogametic females versus heterogametic males (tiger and leopard) (*SI Appendix*, Fig. S2).

Quantitative analysis of the Hi-C matrices was then performed to evaluate consistency of the observed patterns. Eigenvector values were obtained from Juicer outputs for the Hi-C matrices at 500-kb bin resolution. Eigenvector analysis of each felid chromosome confirmed the visual analysis of the Hi-C contact maps, showing strong similarity of values at equivalent genome positions (Fig. 1*B* and *SI Appendix*, Fig. S2) with few compartment changes. Analysis of eigenvectors of the felid X chromosomes identified several A/B compartment shifts (*SI Appendix*, Fig. S2) not directly correlated to sex, with the clouded leopard having the largest number and magnitude of shifts. These results demonstrate that for a given cell type and under standardized experimental conditions, Hi-C contact patterns are highly consistent for orthologous chromosomes within the Felidae family, although X may show a high degree of variation (e.g., clouded leopard). Chromatin conformation in felid leukocytes thus appears to be highly conserved over a maximum divergence time of 15 million years (My) (*SI Appendix*, Fig. S1). Inversions in felids did not appear to disrupt chromatin conformation over long-range distances on the same chromosome, although chromatin conformation changes at resolutions higher than those permitted by our analysis cannot be excluded.

### Conservation of Chromatin Conformation in Canidae.

**Fig. 2.**
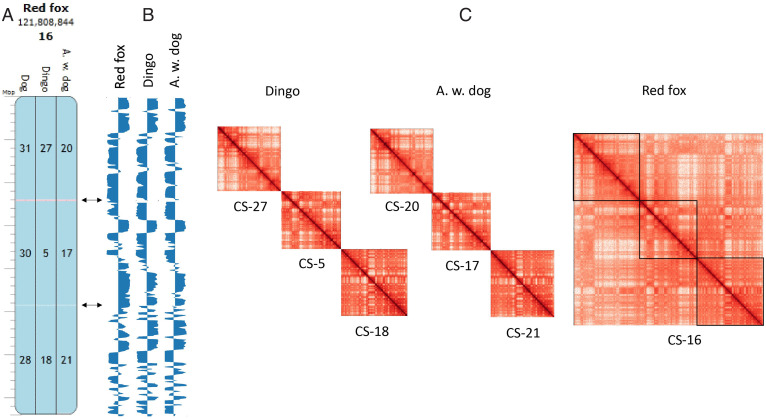
Comparative analysis of chromatin conformation in canids. Dog chromosomes, and dingo and African wild dog C-scaffolds orthologous to red fox C-scaffold 16 are shown as examples. (*A*) Homologous synteny blocks of the three canids visualized in Evolution Highway at 300-kb resolution. Blue indicates same sequence orientation as the reference genome. Pink depicts chromosome inversions. Numbers represent the scaffold identifier of the target species. (*B*) Eigenvector values of each species aligned to the red fox reference genome at 500-kb resolution. Arrows point to compartment variation. (*C*) Juicer plots of C-scaffolds for dingo, African wild dog, and red fox. Color intensity reflects the frequency of interactions between pairs of loci on C-scaffolds (range 1 to 1,000 for each map). Similar comparisons for all other canids C-scaffolds are shown in *SI Appendix*, Fig. S3. Alignment coordinates can be found in Dataset S5.

As done for the felids, we conducted the same visual analysis of Hi-C contact map patterns in individual females of three species from the Canidae family: dingo (2*n* = 78), African wild dog (2*n* = 78), and red fox (2*n* = 34 + 0–8 B chromosomes). These species had Hi-C data available in DNA Zoo produced from leukocyte DNA in the same manner as the felids. The canids have diploid numbers distinctly different from those of the felids, with red fox having less than half the number of chromosomes as dingo and African wild dog. Chromosome orthologies were identified by aligning whole-genome sequences of domesticated dog (2*n* = 78), dingo, and African wild dog to the red fox genome (Fig. 2, *SI Appendix*, Fig. S3, and Datasets S4 and S5). The domesticated dog assembly was used to standardize the numbering of orthologous chromosomes because dingo does not have sequence scaffolds that are physically assigned to chromosomes in its karyotype (*SI Appendix*, Fig. S3 and Datasets S4 and S5). However, we did not have standardized Hi-C data for the domesticated dog reference genome, so dingo Hi-C data were used for the analysis (dingo and domesticated dog are related subspecies). When the Hi-C maps of dingo and African wild dog C-scaffolds were visually compared, all 38 autosomes and X could be readily identified (Fig. 2, *SI Appendix*, Fig. S3, and Datasets S4 and S5). C-scaffold orthology was well supported by the analysis of eigenvectors (*SI Appendix*, Fig. S3). The three canid X chromosomes showed Hi-C pattern similarity, allowing for easy distinction from the autosomes. However, there was considerable variation in eigenvectors unrelated to sex and was most pronounced for the dingo in the three-way comparison (*SI Appendix*, Fig. S3 and Datasets S4 and S5).

We then compared Hi-C maps of African wild dog and dingo to the red fox to investigate whether chromosome fusions and fissions might affect chromatin conformation (Fig. 2, *SI Appendix*, Fig. S3, and Datasets S4 and S5). Each red fox autosomal C-scaffold was found to have a Hi-C pattern that is a composite of two or more C-scaffolds present in African wild dog and dingo (Fig. 2 and *SI Appendix*, Fig. S3). For example, red fox C-scaffold 16 was found to have a Hi-C pattern that is a composite of three C-scaffolds present in African wild dog and dingo, which correspond 1:1 to three dog chromosomes (C-scaffolds 31, 30, and 28) (Fig. 2 and *SI Appendix*, Fig. S3). We show that the red fox karyotype resulted from 26 fusions and 4 fissions as compared to the dog genome, confirming previous results (26), and that the fusion on red fox C-scaffold 8, which is orthologous to dog chromosome 18, underwent another internal rearrangement (*SI Appendix*, Fig. S3 and Datasets S4 and S5). In addition, shared and species-specific inversions were identified (*n* = 28) (Datasets S4 and S5). Although the comparison method did not allow high-resolution analysis of compartment boundaries, primarily due to depth of sequencing data and lack of genome alignment at the breakpoint regions, comparison of eigenvectors revealed compartment switches in red fox compared to dingo and African wild dog for several orthologs (e.g., see red fox C-scaffolds 2, 3, 9, and 11) (*SI Appendix*, Fig. S3). However, eigenvectors were similar enough that orthologous segments could readily be discerned between red fox and the other canids (Fig. 2*C*, *SI Appendix*, Fig. S3, and Datasets S4 and S5). Canid autosomes thus show conservation of chromosome-scale 3D chromatin conformation over 14 My of evolution (*SI Appendix*, Fig. S1), but the fusions and fissions that led to the red fox karyotype appear to be associated with changes in 3D chromatin architecture relative to the other canid species.

### Conservation of Chromatin Conformation in Ursidae.

Black bear, polar bear, and grizzly bear Hi-C contact maps were analyzed using black bear as a reference. These species diverged from a common ancestor ∼6 Mya (*SI Appendix*, Fig. S1) and have the same diploid chromosome number (2*n* = 74). The three ursids showed nearly identical Hi-C contact map visual patterns for the 37 C-scaffolds identified in each species, demonstrating that all orthologous bear C-scaffolds could be distinguished by their Hi-C contact maps. No fusions or fissions were identified, but seven and nine inversions were found in grizzly bear and polar bear relative to black bear (Datasets S6 and S7), respectively. The Hi-C contact map patterns were strongly supported by eigenvector analysis, which showed high overall similarity between the bear species for all orthologous C-scaffolds (*SI Appendix*, Fig. S4 and Datasets S6 and S7). The Y chromosome was not included in our analysis because only one representative individual was male (grizzly bear) (Dataset S1). The polar bear X chromosome showed extensive differences in eigenvectors, indicating underlying A/B compartment switches (*SI Appendix*, Fig. S4).

### Conservation of Chromatin 3D Structure across the Canidae, Felidae, and Ursidae.

**Fig. 3.**
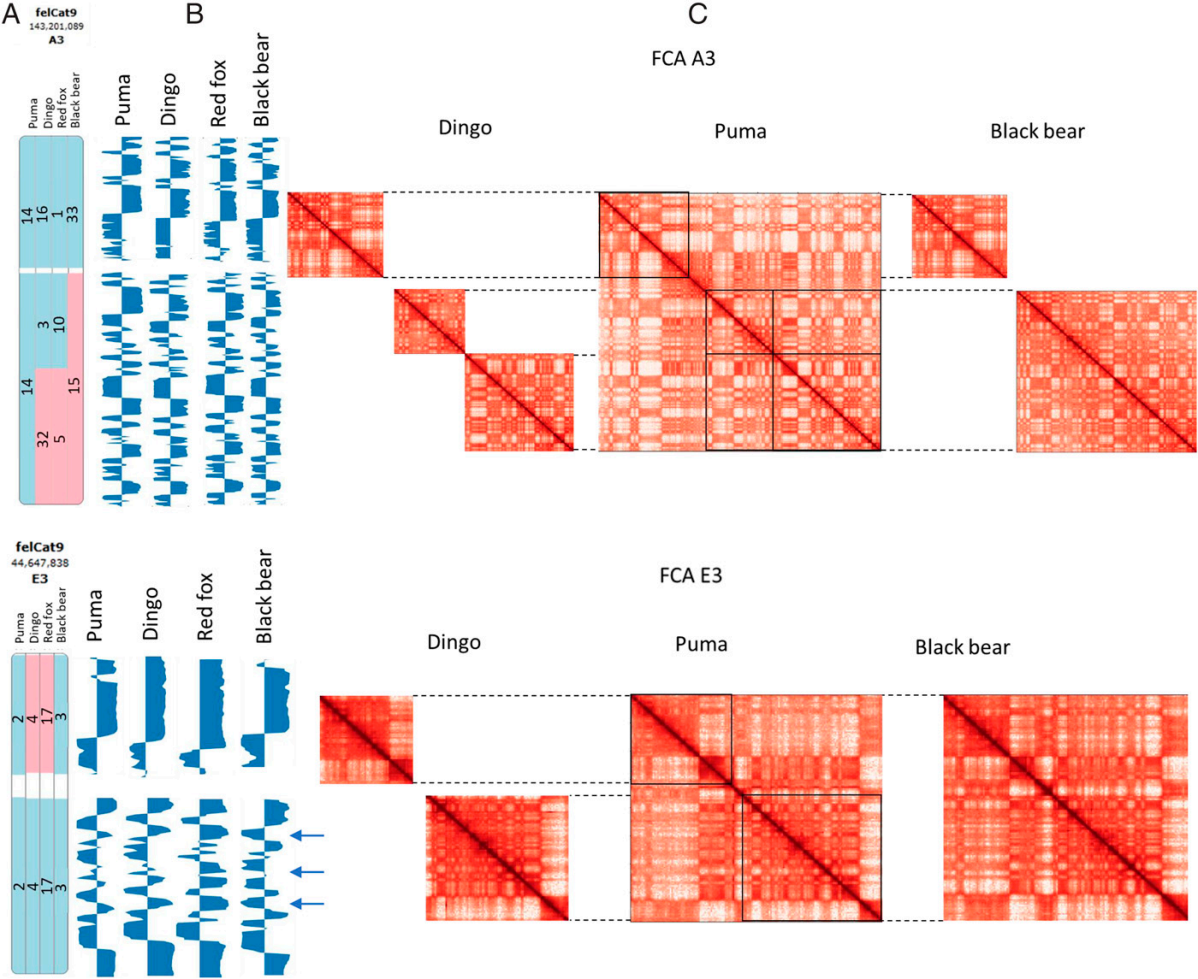
Comparative 3D chromatin conformation analysis across carnivore families. (*A*) Alignments of representative species of felids, ursids, and canids (p uma, dingo, red fox, and black bear) C-scaffolds to cat chromosomes A3 (*Upper*) and E3 (*Lower*). Blue indicates the homologous synteny blocks in the same sequence orientation. Pink depicts chromosome inversions. Numbers indicate C-scaffold identifiers. (*B*) Eigenvector values of each species aligned to the cat reference genome at 500-kb resolution. Arrows point to compartment variation. Eigenvectors within inverted regions were reoriented to be consistent with the reference genome. (*C*) Comparative analysis of Hi-C maps of orthologous C-scaffolds and C-scaffold fragments. Boxed areas on the puma Hi-C map demarcate the boundaries of orthology corresponding to dingo and black bear Hi-C maps, as shown in part A. Corresponding numbers of C-scaffolds can be found in the Evolution Highway images (part A) and in Table 1. Color intensity reflects the frequency of interactions in the C-scaffolds (range 1 to 1,000 for each map). The inverted region in the dingo Hi-C map was reoriented for comparison. Alignment coordinates can be found in Dataset S3.

To address the question of whether chromatin conformation is conserved across the three carnivore families, we first built a table of chromosome orthologies for all species studied. The cat genome was selected as a reference because its karyotype is closer to the ancestral carnivore karyotype than those of canids or ursids (27). A summary of all orthologous relationships based on LastZ alignments between cat chromosomes and C-scaffolds in other felid, canid, and ursid species is shown in Fig. 3. As presented above, all felid species’ C-scaffolds showed 1:1 orthology with cat chromosomes, and chromatin conformation results were concordant. Among the other carnivore families, 1:1 orthology to cat autosomes was found for only three ursid C-scaffolds (Table 1 and *SI Appendix*, Fig. S5). The X chromosome showed 1:1 orthology to the cat for all species (Table 1 and *SI Appendix*, Fig. S5). Although there were no other 1:1 orthologies with cat chromosomes in ursids and canids, orthologous subchromosomal C-scaffold fragments were readily identified. The number of subchromosomal C-scaffold fragments orthologous to cat chromosomes ranged from one to nine in the canids and one to five in the ursids (Table 1 and *SI Appendix*, Fig. S5), as would be expected for the higher number of chromosomes among the species in these two families, with the exception of the red fox. Within the canids, C-scaffolds of dingo and African wild dog showed complete 1:1 orthology, while red fox C-scaffolds were found to have different orthologous relationships due to chromosome fissions, fusions, and translocations (Table 1, *SI Appendix*, Fig. S5, and Datasets S3 and S4). Within the ursids, C-scaffolds for all three species showed complete 1:1 orthology (Table 1, *SI Appendix*, Fig. S5, and Datasets S3–S6).

**Table 1.** 3DSC: Orthologous relationships for all species’ scaffolds using the cat genome as a reference

Cat Chr	P	C		L	T		CL	Dingo (2*n* = 78)	African wild dog (2*n* = 78)	Red fox (2*n* = 34)	Black bear (2*n* = 74)	Grizzly bear (2*n* = 74)	Polar bear (2*n* = 74)
	(2*n* = 38)												
A1	9	1	12			13	1	28c+30 + 31b+38b+10a +8c+39c+1a+21b	30a+1 + 34b+32b+2b +29c+11c+37b+19b	3b+15b+7b+14b+9e +11b+12c+17c	36b+27 + 12	36b+35 + 10	11a+6 + 16
A2	15	4	5			14	4	7 + 14b+15a+31a	26 + 8a+28b+34a	3a+7a+8a	28a+2	14a+5	14a+4
A3	14	9	6			12	9	16 + 36a+3a+32a	23 + 24b+33b+5b	11a+1b+10d+5a	33 + 15	15 + 16	23 + 7
B1	5	3	4			6	3	22a+14a+28b+13a+29a +33 + 35a+39a	13a+8b+30b+36d +7a+6 + 3a+11a	10a+7c+3c+6f+12a+9c+11d	35 + 31+32	26 + 1+8	28 + 12+9
B2	18	6	1			10	6	26b+11 + 2b	15a+35 + 38b	14c+2a	11 + 9+14	22 + 30+4	32 + 30+15
B3	4	7	11			5	7	39b+5 + 13c+38a	11b+17 + 36b+32a	11c+16b+6d+15a	6 + 7+8 + 49+10	20 + 29+16 + 6206+25	29 + 36+38 + 37+25
B4	6	8	3			11	8	8d+12 + 3b+13b	29d+18 + 33a+36c	9d+5c+10c+6e	34 + 19+18	31 + 27+24	31 + 27+22
C1	16	2	7			7	2	9b+8a+13d+4a+32b +29b+23 + 22b+28a	31c+29a+36a+25a+5a +7b+14 + 13b+30c	14g+9g+6c+17a+5b +8e+10b+3d	17 + 26+36a+13	18 + 9+36a+2	33 + 13+11b+2
C2	17	5	2			4	5	27 + 24+36b+21a	20 + 16+24a+19a	16a+2b+1c+17d	25 + 29	3 + 33	5 + 21
D1	12	11	8			8	11	9d+20 + 15b	31a+9 + 28a	14e+1a+8c	5 + 16	32 + 21	17 + 24
D2	19	14	9			9	14	17a+1b+18	22b+37a+21	6b+12b+16c	37	12	8
D3	8	13	10			19	13	17b+2a+34a	22a+38c+4a	6a+8d+4b	4 + 30	17 + 34	34 + 19
D4	13	12	15			3	12	2c+10b+6b	38a+2a+27a	2c+14a+9b	22 + 24	28 + 23	35 + 18
E1	1	18	16			17	18	9c+6a	31b+27b	14f+9a	28b+20	14b+19	14b+26
E2	7	17	18			16	17	2d+8b+9a	29b+31d	2d+9f+14h	23	13	20
E3	2	19	17			15	19	4b	25b	17b	3a	11b	3a
F1	3	16	13			18	16	34b+19	4b+12	4c+8b	3b	11a	3b
F2	11	15	14			2	15	26a+25 + 35b	15b+10 + 3b	14d+4a	21	7	10
X	10	10	19			1	10	37	39	13	1	37	1

C, cheetah; CL, clouded leopard; L, leopard; P, puma; T, tiger.  Numbers in columns are C-scaffold identifiers in each respective assembly. Letters after C-scaffold numbers indicate subchromosomal fragments in order of their position.

To investigate the maintenance of 3D chromatin conformation at the ordinal level, we compared Hi-C contact maps and eigenvector values for all carnivore C-scaffolds that are orthologous to cat chromosomes (Fig. 3, Table 1, *SI Appendix*, Figs. S5 and S6, Dataset S3). Examples of conservation of carnivore chromatin structure as observed for orthologs of two cat chromosomes, A3 and E3, are shown in Fig. 3. Cat A3 has 1:1 orthology with puma C-scaffold 9 and four and two orthologous C-scaffolds or C-scaffold fragments in canids and ursids, respectively (Fig. 3 and Table 1). Hi-C contact map patterns and eigenvectors are near-identical for all studied carnivore C-scaffolds or C-scaffold fragments that are orthologous to cat A3 (Fig. 3), despite fission of an ancestral carnivore chromosome corresponding to A3 in black bear, and multiple fissions and other rearrangements in the canids.

Cat E3 exhibited some differences in chromatin conformation between carnivore families. There is a single C-scaffold fragment orthologous to E3 present in all carnivores studied (Fig. 3 and Table 1). However, in canids and ursids, one or more ancestral fusions joined the orthologous segments to other chromosomes (Table 1). In canids, there is also an inversion that corresponds to E3p. Examination of eigenvector plots of E3p orthologs (Fig. 3) revealed a very similar intrachromosomal pattern between the families even for the canids, although there may be some compartment shifting at the boundaries in the canids and ursids relative to puma. Given the size differences of this orthologous region among the species, it is not possible to make a definitive statement about shifting of compartments boundaries from this level of analysis. By contrast, analysis of the segment orthologous to E3q revealed a small number of compartments shifts in red fox and black bear, but the overall visual pattern and eigenvectors were similar, and the differences did not confuse definition of the orthologous chromosome segments. Thus, overall 3D chromatin conformation and compartment definitions in orthologs of cat E3 appear to be well conserved, even for Canidae, which had multiple chromosome rearrangements in its evolutionary path.

Analysis of Hi-C contact map patterns and eigenvectors of all species showed that for most orthologous C-scaffolds or C-scaffold fragments in canids and ursids, the corresponding A/B compartment definitions were highly similar, indicating conservation of chromosome-level chromatin conformation dating to the ancestral carnivore ∼54 Mya (*SI Appendix*, Fig. S1). Even when C-scaffolds were fragmented by chromosome rearrangements, it was still possible to identify and order orthologous segments based on the Hi-C contact map patterns. However, within orthologous chromosome segments, a relatively small number of across-family differences in A/B compartments were discerned (Fig. 3 and *SI Appendix*, Fig. S6). These differences were most pronounced for the X chromosomes and less so for several autosomal C-scaffolds of red fox, recapitulating within-family differences, and black bear (*SI Appendix*, Fig. S7). In general, canid and ursid orthologs of the carnivore ancestral-type cat chromosomes A1, B1, B4, and C1 showed the greatest amount of between-families variation in 3D chromatin structure (*SI Appendix*, Fig. S6). These chromosomes also have the greatest numbers of rearrangements relative to the cat/ancestral carnivore chromosome configuration (Table 1).

### Three-Dimensional Comparative Scaffotyping.

Conservation of 3D chromatin structure allowed for the unambiguous assignment of C-scaffold orthology to cat chromosomes within and between carnivore families (Table 1 and Datasets S2, S4, and S6). We call this method 3D comparative scaffotyping, or 3DCS. On the basis of 3DCS, we developed and used a scheme for naming of C-scaffolds of carnivores (Table 1 and *SI Appendix*, Table S1).

## Discussion

Chromosome rearrangements are a hallmark feature of genome evolution (28). However, there is limited and conflicting information on whether there is conservation of 3D chromatin conformation between species and whether chromosome rearrangements affect chromatin structure (29). Our study addressed the question of whether 3D chromatin conformation is evolutionarily conserved at the scale of whole chromosomes. This new dimension in comparative genomic analysis can reveal whether there are spatial constraints on chromosome evolution. Understanding how evolutionary processes affect 3D chromatin at the level of chromosome structure can provide a deeper understanding of how chromosome rearrangements may contribute to changes in gene regulation, disease processes, the evolution of lineage-specific traits, and speciation. Our method for comparing 3D chromatin conformation, used together with DNA sequence alignment, also supports the unambiguous identification of orthologous chromosomes and subchromosomal syntenic fragments between related species.

The Hi-C data in our study were produced in a standardized manner, using leukocyte DNA collected from 11 representative species in three carnivore families. This allowed us to minimize experimental variability and focus on three carnivore taxonomic families with known variation in karyotype between them. The patterns of synteny among extant carnivore species thus served as a template for investigating conformational changes in chromatin compartmentalization resulting from lineage-specific chromosome rearrangements. Within and between the three carnivore families, 3D chromatin conformation was found to be highly conserved for most orthologous chromosomes, C-scaffolds, and subchromosomal fragments, even after ancestral chromosome fusions, fissions, and inversions that occurred over 54 My divergence from a common ancestor. Our results demonstrate that in carnivores, and likely within other vertebrate taxonomic groups, chromosome-scale 3D chromatin conformation is under strong evolutionary constraint for autosomes. Our results significantly extend results obtained at much smaller chromosomal scale, showing that many TADs, which are on the order of ∼1 Mbp (5), are conserved between human and mice (5, 8, 30) and between human and gibbon (10). This constraint adds to the evidence that 3D chromosome architecture in the interphase nucleus is an important hierarchical organizing principle for maintaining genome stability and function (1).

One of the most important unresolved issues in genome evolution is how chromosome rearrangements affect genome and organismal functions. The comparison of the Hi-C contact maps of the five felid species, which all have identical diploid number, showed that orthologous C-scaffolds have similar visual and corresponding eigenvector patterns (Fig. 1 and *SI Appendix*, Fig. S2). As shown by the eigenvector analysis, it was striking that chromatin conformation, even at the compartment level, was nearly identical for all felids (Fig. 1 and *SI Appendix*, Fig. S2). The 30 inversions that distinguish felid species, even the larger inversions, such as the one on tiger scaffold 17 relative to cat, did not produce distinct changes in the overall chromatin conformation pattern. These findings were generally consistent for the canids and ursids, which have more variable karyotypes (Fig. 2 and *SI Appendix*, Figs. S3 and S4). For example, dingo and African wild dog have identical diploid number and their autosomes showed consistent 3D conformation. In red fox, which underwent 26 chromosomal fusions and four fissions relative to dog (26), 3D chromatin organization of C-scaffolds was largely unchanged when compared to orthologous C-scaffolds of African wild dog and dingo. The 3D patterns were even similar for chromosomes that underwent ancestral fusions in the red fox lineage. However, these fused chromosomes appear to have established new long-range intrachromosomal interactions in red fox (Fig. 2), as observed for muntjac species whose chromosomes underwent multiple tandem fusions (31). We cannot exclude the possibility that these interactions might still be present as interchromosomal interactions in the nonfused orthologs in dingo and African wild dog.

At the compartment level, red fox had the highest number of compartment changes within canids. The additional chromosome rearrangements in the red fox lineage, compared to the two dogs, could explain the increased number of compartment changes observed for red fox. This is similar to what was observed for Indian muntjac, which has compartment shifts associated with tandem fusions (31). From an evolutionary perspective, these rearrangements may be responsible for changing interactions between regulatory elements and genes, thus playing a role in defining lineage-specific gene expression (8). Across all families, a small number of compartments shifts were also observed in syntenic regions, which may represent lineage-specific compartment shifts that affect the transcription of specific genes, or could be related to small inversions beyond the resolution of the analysis. We can thus conclude that within carnivore families, inversions do not change the general pattern of 3D chromatin conformation at the chromosome level. However, new long-range intrachromosomal interactions appear to be established in chromosomes that have undergone ancestral fusions. These results are consistent with recent results in mice showing that chromosome fusions affect 3D chromatin topology and interactions (13).

By comparing Hi-C contact maps and associated eigenvectors across the three carnivore families, we identified striking conservation of 3D chromatin conformation within orthologous subchromosomal fragments over 54 My of carnivore evolution (Fig. 3, Table 1, and *SI Appendix*, Figs. S5 and S6). The C-scaffolds that are orthologous to cat chromosome A3 are a fitting example of cross-family conservation of 3D chromatin conformation (Fig. 3). However, among the autosomes, eigenvector analysis showed that there are cases of clear compartment shifts in orthologous subchromosomal segments even though the Hi-C contact maps are indistinguishable by eye. Most of the differences in chromatin conformation between families were due to patterns observed in bears and red fox (Fig. 3 and *SI Appendix*, Fig. S6). Many of these apparent compartment shifts are associated with chromosome rearrangements relative to the more ancestral cat genome. For example, eigenvector analysis of canid C-scaffolds orthologous to cat E3p, showed only small differences in chromatin conformation relative to other species (Fig. 3 and *SI Appendix*, Fig. S3). However, C-scaffolds orthologous to cat E3q, did show more prominent variation in compartment definitions in canids and also in black bear. While the inverted segments cannot be observed in the eigenvector plots because they were reoriented to make for easier comparison, the locations of the inverted segments can be determined from the Evolution Highway ideograms and the accompanying genome coordinates (Fig. 3 and *SI Appendix*, Fig. S6).

For orthologous chromosome segments, our analysis showed that when there is an inversion in one or more lineages, chromosomal interactions within the inverted segment are generally maintained. However, when an orthologous fragment becomes part of a different chromosome due to translocations fissions, and fusions, such as the segments orthologous to E3q described above (Fig. 3; see also Table 1), long-distance interactions are broken, and new long-distance interactions must be established (if joined to another chromosome). These results suggest different local 3D chromatin folding of red fox and black bear C-scaffolds orthologous to cat E3q as a result of a canid-specific inversion corresponding to E3p, and ancestral fissions that occurred in canids and ursids. These rearrangements caused part of the same canid and ursid C-scaffolds to become orthologous segments corresponding to other cat chromosomes (C1 and F1, respectively) (Table 1 and *SI Appendix*, Fig. S3). This may have functional significance because intrachromosomal interactions have been shown to create contact between promoters and enhancers located several thousands of bases apart (32). In addition, changes in TADs have been shown to be associated with chromosome rearrangements (33–35). Our findings reinforce the conclusion that the maintenance of chromatin conformation is functionally and evolutionarily important, and its structural constraints extend from the level of TADs to subchromosomal regions.

While the visual Hi-C contact maps of the X chromosome were similar enough for all species to distinguish X from the autosomes, eigenvector analysis suggested differences in X chromatin conformation within and between families and sex (*SI Appendix*, Figs. S6 and S7). Rearrangements apparently do not play a role in these differences, because there are only a few small inversions that differentiate the X chromosomes of the species studied. Random or nonrandom X chromosome inactivation and differences in Hi-C data coverage of the X in males and females may contribute to the variation in contact map patterns. In humans and mice, the active X (Xa) chromosome has typical compartment structure, while in cell lines, the inactive X (Xi) lacks clear delineation of compartment boundaries (36, 37). In Xi, TADs are less abundant and show a bipartite organization in two megadomains that are absent from Xa (2, 38, 39). In felids, puma, cheetah, and clouded leopard, Hi-C contact maps exhibited the expected bipartite organization, confirming observations in human and mouse (2, 38–40), and appear to be a combination of signals from Xa and Xi. The female clouded leopard was an outlier among felids with respect to X chromosome compartments. The ursids’ X chromosomes also lacked clear compartment definitions. These observations may be due to nonrandom X inactivation or technical issues relating to the assembly itself or the Hi-C dataset. Surprisingly, the bipartite organization was less evident in canids and ursids, suggesting a different 3D structure of X. Incomplete X inactivation in meiotic cell lines was previously observed in the dog (41), so we do not exclude possible lineage-specific changes in 3D structure for the canid X chromosomes. Male Hi-C maps (tiger, leopard, and grizzly bear), which have only Xa, showed more defined compartments, although the compartment structure was very distinct between the two male felids and the male grizzly bear. Further studies are clearly needed to understand the comparative 3D chromatin architecture of carnivore X chromosomes.

For our study, we took advantage of the growing collection of standardized Hi-C–based whole-genome assemblies in the DNA Zoo (15). These assemblies, which include C-scaffolds, are well-suited for studying chromosome evolution. However, the relatively low coverage (∼24 to 27×) (Dataset S1) of Hi-C data available for the species included in this study hindered the analysis of chromatin conformation at resolutions higher than compartment level, especially detection of TADs and interchromosomal interactions. Interchromosomal interactions are involved in promoting the formation of chromatin domains, such as centromere clusters, but they are also involved in gene regulation (e.g., interferon-related genes, olfactory receptor genes, and X-inactivation) (42, 43). New interchromosomal contacts might be crucial to accommodating changes in chromatin conformation resulting from chromosome rearrangements, which in turn might affect gene-enhancer interactions in some lineages. Hi-C coverage of ∼200× would facilitate higher-resolution comparative studies of the effect of chromosome rearrangements on compartment boundaries, TAD definitions, and interchromosomal interactions, which would provide greater insights into the evolutionary dynamics of 3D chromatin conformation and their possible role in rewiring transcriptional networks (2, 13, 44).

A significant problem faced by large-scale de novo genome sequencing projects, such as the Earth BioGenome Project (19), is the assignment of DNA sequence scaffolds to chromosomes when the karyotype of the newly sequenced species is unknown. We propose that our methodology (3DCS) can be used to identify and name chromosomes of species with unknown karyotype (see *SI Appendix* for detailed discussion and rules-based scaffotyping system). Using the cat genome as the most ancestral genome of carnivores (27), C-scaffolds from all species that aligned to the cat genome could be named according to their orthologous relationships (Table 1). For applying 3DCS, the reference genome should be the most ancestral in the clade being studied (family-level for mammals would be optimal) and should have a known karyotype with >90% of the sequence anchored to chromosomes. Unless there is a full complement of 1:1 chromosome orthologs across species within the clade, naming according to the nomenclature of the reference genome should be avoided. When there are no 1:1 relationships, naming according to the reference genome will be very complicated because fusions, fissions, and translocations will change chromosome numbers, sizes, and comparative organization. For most species, naming by scaffold size will be appropriate. A look-up table with determined orthologous relationships of chromosomes and C-scaffolds, such as the one we produced with our data (Table 1), will be the most efficient way of drawing evolutionary inference from chromosome nomenclature.

We have shown that chromatin conformation is largely conserved for orthologous whole chromosomes and C-scaffolds within three carnivore families. When compared to felid chromosomes, which represent the ancestral karyotype within Carnivora, comparisons across families showed that orthologous subchromosomal fragments retain the same intrachromosomal contacts within the fragments despite one or more lineage-specific rearrangements. In chromosomes that are rearranged during evolution, new long-range intrachromosomal contacts must also be acquired. The conserved contacts appear to be stable over 54 My since the divergence of these carnivore species from a common ancestor. Our results suggest that the chromosome-level conservation of 3D chromatin conformation is as biologically significant as the conservation of underlying TADs. This higher-order organization of chromosomes appears to reflect requirements for maintaining chromosome structure, organization of chromosomes in the nucleus, regulation of gene expression, and genome stability (1, 45). Changes that occur during evolution are likely to disrupt genome anatomy and function, and consequently may be involved in lineage-specific changes in phenotype that accompany speciation. Although our study was limited to carnivores, on the basis of comparative genome organization in mammals, we expect that these relationships will hold true for other mammal orders. It will be important to reveal how deep in mammalian evolution chromatin conformation is conserved, when such changes occurred, and whether ancestral chromosomes differ in their timing and tempo of evolution of their 3D structure.

## Methods

### Whole-Genome Alignment.

Chromosome or scaffold-level assemblies of the following species were used for this study: *Neofelis nebulosa*, clouded leopard; *Panthera pardus*, leopard (46); *Panthera tigris*, tiger (47); *Acinonyx jubatus,* cheetah (48); *Puma concolor,* puma (https://www.ncbi.nlm.nih.gov/nuccore/QAVW00000000.1); *Canis lupus dingo*, dingo (https://www.ncbi.nlm.nih.gov/nuccore/QKWQ00000000.1); *Lycaon pictus*, African wild dog (49); *Vulpes vulpes*, red fox (50); *Ursus americanus*, black bear (51); and *Ursus arctos*, grizzly bear (52); *Ursus maritimus*, polar bear (53) (Dataset S1). All assemblies were obtained from the DNA Zoo database (https://www.dnazoo.org/assemblies, cutoff date, July 2019). The cat (*Felis catus*; felCat9) and the dog (*Canis lupus familiaris*; canFam3) genomes were obtained from the University of California, Santa Cruz (UCSC) repository (https://genome.ucsc.edu/). Chromosome and C-scaffold assemblies of the cat, dog, red fox, and black bear genomes were used as reference genomes for whole-genome alignment of scaffold assemblies of each species. The cat genome was used as reference for every species but the dog. The dog and red fox genomes were used as references within the canid family (dog, dingo, African wild dog, and red fox). The black bear genome was used as the reference within the ursid family (polar bear and grizzly bear). Prior to alignment, all genomes were filtered for scaffolds shorter than 50 kb using faFilter and then converted to .*2bit* format using faToTwoBit tool from the Kentutils package (54). All whole-genome pairwise alignments were generated using LastZ (v1.04) (55) with the following parameters C = 0 E = 30 H = 2,000 K = 3,000 L = 2,200 O = 400. The pairwise alignments were converted into the UCSC chain and net formats with axtChain (parameters: -minScore = 1000 -verbose = 0 -linearGap = medium) followed by chainAntiRepeat, chainSort, chainPreNet, chainNet, and netSyntenic, all with default parameters (56). Pairwise synteny blocks were defined using maf2synteny (57) at 300-kb resolution (Datasets S3, S5, and S7).

### Identification of Chromosome Orthologies.

The pairwise synteny blocks of alignments between the reference genome and C-scaffolds were uploaded and visualized using the Evolution Highway comparative chromosome browser (eh.informatics.illinois.edu/). All orthologous relationships between C-scaffolds of felid, canid, and ursid assemblies and the reference genome chromosomes (cat and dog) or C-scaffolds (black bear and red fox) were tabularized (Table 1 and Datasets S2, S4, and S6). Scaffold numbers are those reported for the individual assemblies (15). Hi-C contact maps and eigenvectors (described below) were used for identifying patterns specific to each C-scaffold and as support for the definition of orthologous relationships.

### Identification of Chromosome Rearrangements.

We detected rearrangements within each family using output block alignment files obtained from each pairwise genome alignment. Rearrangements in felids were identified using the domesticated cat as reference genome (Dataset S2); rearrangements in canids were identified using red fox as the reference genome (Dataset S4); rearrangements in ursids were identified using black bear as the reference genome (Dataset S6).

### Hi-C Data Analysis.

For Hi-C data analysis, 280 million read pairs of Hi-C data were downloaded for each species from DNA Zoo NCBI BioProject PRJNA512907 using fastq-dump (v2.10.5, https://trace.ncbi.nlm.nih.gov/Traces/sra/sra.cgi?view=software) and processed using the Juicer platform (version 1.6.2) (58, 59) with default parameters. The pipeline uses BWA (40) to map reads and remove read duplicates. Each species' genome was used as a reference genome to map Hi-C reads and contact matrices were generated. Hi-C contact maps (.hic format) were inspected with Juicebox (v1.11.08) (58). Hi-C maps were converted to .cool format using hicConvertFormat from the HiCExplorer software (v3.1) (60). Hi-C maps were plotted using the software hicPlotMatrix from HiCExplorer with the following parameters: -region <genomic coordinates> –log –colorMap Reds –vMax 1000 –dpi 720 –bigwig.

Hi-C contact maps of each C-scaffold were scaled to give each map the same physical dimensions. Hi-C maps were aligned chromosome by chromosome to the corresponding reference genome chromosomes using the LastZ pairwise C-scaffold alignments as described above. The Hi-C contact map patterns were used for the first level visual comparison of C-scaffolds.

### Eigenvector Analysis.

Eigenvector values of all analyzed species were obtained from Hi-C maps using the eigenvector option from Juicer (v1.11.09) (58) at 500-kb bin resolution for each individual C-scaffold. Custom parameters were used: java -jar juicer_tools_1.11.09_jcuda.0.8.jar *eigenvector KR BP 500000 -p.* The eigenvector used corresponds to principal component 1 of the Pearson correlation of the contact matrix. Eigenvector analysis gives a numeric translation of the patterns shown in the Hi-C maps, allowing for the comparison of chromatin conformation between different species. Eigenvector values of each species were aligned manually to the respective chromosomes in the reference genome for each family as displayed in Evolution Highway. Direction of A and B compartments was arbitrarily assigned in order to have the same orientation for all species. For cross-family comparisons, eigenvectors of puma, dingo, red fox, and black bear were aligned manually to cat chromosomes and displayed as described above. To create comparative displays that permitted visual comparisons, eigenvectors within inverted regions were reoriented to correspond to the reference genome. The 3DCS method used a combination of all datatypes (e.g., LastZ alignments, Hi-C contact map patterns, and eigenvectors).

## Supplementary Material

Supplementary File

Supplementary File

Supplementary File

Supplementary File

Supplementary File

Supplementary File

Supplementary File

Supplementary File

## Data Availability

All study data are included in the main text and/or supporting information. Previously released data were used for this work (NCBI BioProject PRJNA512907). Genome assemblies and sequence data for clouded leopard, leopard, tiger, cheetah, puma, dingo, African wild dog, red fox, black bear, grizzly bear, and polar bear are used with permission from the DNA Zoo Consortium (https://www.dnazoo.org/assemblies).
