# Supplementary material for: Conservation of chromatin conformation in carnivores
Source: Proc Natl Acad Sci U S A. 2022 Feb 25;119(9):e2120555119. doi: 10.1073/pnas.2120555119 (PMC8892538; doi:10.1073/pnas.2120555119)
Supplement: Supplementary File [file pnas.2120555119.sapp.pdf]

PNAS
www.pnas.org

Supplementary Information for

Conservation of chromatin conformation in carnivores

Marco Corbo[1], Joana Damas[1], Madeline G. Bursell[2,3], Harris A. Lewin[1,4,5]

Harris A. Lewin

Email: lewin@ucdavis.edu

This PDF file includes:

Supplementary text
Table S1
Figures S1 to S7
Legends for Figures S1 to S7
Legends for Datasets S1 to S7
SI References

Other supplementary materials for this manuscript include the following:

Datasets S1 to S7

Supplementary Text

3D-comparative scaffotyping

Typically, karyotypes are determined by cytogenetic methods and definitive chromosome assignments of genes and scaffolds are made by physical mapping methods, such as fluorescence in situ hybridization. Hi-C and other chromatin capture conformation methods have proven useful for producing chromosome-scale scaffolds, and thus have utility for estimating haploid chromosome number. However, confirming that DNA scaffolds created by chromatin conformation capture methods represent complete chromosomes or chromosome arms can be complicated by assembly errors, such as false joins or missed joins (1), thus leading to errors in karyotype estimation. In addition, most chromosome-scale assemblies have hundreds of unplaced scaffolds (1). This presents a dilemma for the naming of C-scaffolds as chromosomes, particularly when C-scaffolds are missing centromeric and telomeric sequences. Thus, although assemblies facilitated by scaffolding methods are good for estimating chromosome number, the estimates may not be definitive, and that complicates chromosome naming and nomenclature (2).

In the present work we demonstrate that 3D chromatin conformation is highly conserved within and between three carnivore families, and that sequence alignments and Hi-C maps can be used to identify orthologous chromosomes, chromosome arms and chromosome fragments. For the felid, canid and ursid species studied, each of which has a known karyotype, but for which C-scaffolds have not been assigned to chromosomes, we have shown that orthologous C-scaffolds and subchromosomal fragments can reliably be identified by their Hi-C contact map patterns and associated eigenvectors (Figs. 1-3, Table 1; SI Appendix, Figs. S2-S7). Conservation of 3D chromatin structure thus allows for assignment of C-scaffolds of a *de novo* sequenced genome to chromosomes or reference genome C-scaffolds (when C-scaffolds have not been assigned to known chromosomes in a karyotype) through their orthologous relationships (SI Appendix, Datasets S2, S4, and S6). We call this method *3D comparative scaffotyping*, or 3DCS. On the basis of 3DCS, we used a scheme for naming of
C-scaffolds of carnivores (Table 1; SI Appendix, Table S1) that is extensible to other mammalian orders and possibly other eukaryotic taxa.

The reference genome for a clade should be the most ancestral high contiguity chromosome-level assembly available for that species. The 3DCS method should work well at the family level for a majority of vertebrate families. Typically, the reference genome should have diverged from the *de novo* genome by no more than 50 My (3). For numbering the C-scaffolds of a *de novo* sequenced genome according to the scheme in Table S1, for case 1 (top row under the header), C-scaffolds would be numbered the same as in the reference. For case 2, numbering of C-scaffolds should be in the size order that they appear in the *de novo* sequenced genome according to the 1:1 relationships of the orthologs. The look-up table of orthologous C-scaffolds and would include all C-scaffold fragments that align to different chromosomes in the reference genome (representing rearrangements relative to the reference). Cases 3 and 4 represent the most likely scenarios in the current research environment involving sequencing of thousands of new species. We suggest usage of the term *scaffotype* because the scaffold assemblies, even with supporting Hi-C data, are not true karyotypes. Scaffotypes are estimates of karyotypes and should not be confused with representing the full chromosome complement without supporting cytological evidence (2). For case 3, numbering in the *de novo* sequenced genome would be according size based on the scaffotype of the reference genome. For case 4, C-scaffold numbering and C-scaffold fragment assignment would be the same as for case 2.

An alternative naming scheme for the 3DCS correspondence table is one that is based on more basal reconstructed ancestral chromosomes for a given clade, e.g., the eutherian ancestor genome (3). Such a naming scheme can provide important evolutionary context to the naming of species' C-scaffolds at different taxonomic levels. Ideally, the chromosome nomenclature of the future would be based on deep ancestral chromosome reconstructions, which would then permit researchers to relate orthologous C-scaffold and/or chromosomes from many species within a clade.

Implementation of a rules-based system for naming and relating C-scaffolds in mammals and possibly other eukaryotic taxa will greatly facilitate comparative genomic analysis. Furthermore, a system for naming or relating C-scaffolds to a reference genome will solve problems in the naming of chromosomes now encountered by large-scale genome sequencing projects (2). Renaming and renumbering of chromosomes can cause massive confusion in the literature and is difficult to reconcile in public databases. Therefore, we do not recommend naming according to a reference genome unless there are clear 1:1 relationships. However, a system based solely on the size of

chromosomes or C-scaffolds does not provide any evolutionary context to end-users of genome information, much like the standard karyotypes of today. It will be less confusing and lead to fewer problems in the future if look-up tables with all orthologous relationships are created, such as the one we constructed using the cat karyotype as the reference genome for all carnivores (Table 1). To be robust across large numbers of taxa, C-scaffold naming for *de novo* sequenced species with no karyotype or physical mapping information should allow end users to easily relate C-scaffold orthologies in any closely related species. The 3DCS approach provides the necessary information to do this and is thus a powerful new tool for naming C-scaffolds and for understanding chromosome evolution.

Table S1. Schema for numbering chromosomes using 3DSC.

Reference genome assembly type	Reference genome has known karyotype	Physical assignment of markers to known chromosomes (reference)	Karyotype of *de novo* species genome known	1:1 orthologs of all chromosomes or C-scaffolds (reference:*de novo*)	**Numbering of *de novo* species C-scaffolds same as reference**	*De novo* genome chromosome nomenclature
Chromosome or C-scaffold	yes	yes	yes	yes	**yes**	karyotype
Chromosome or C-scaffold	yes	yes	yes	no	**no (size)**	karyotype
C-scaffold	yes or no	no	yes or no	yes	**yes**	scaffotype
C-scaffold	yes or no	no	yes or no	no	**no (size)**	scaffotype

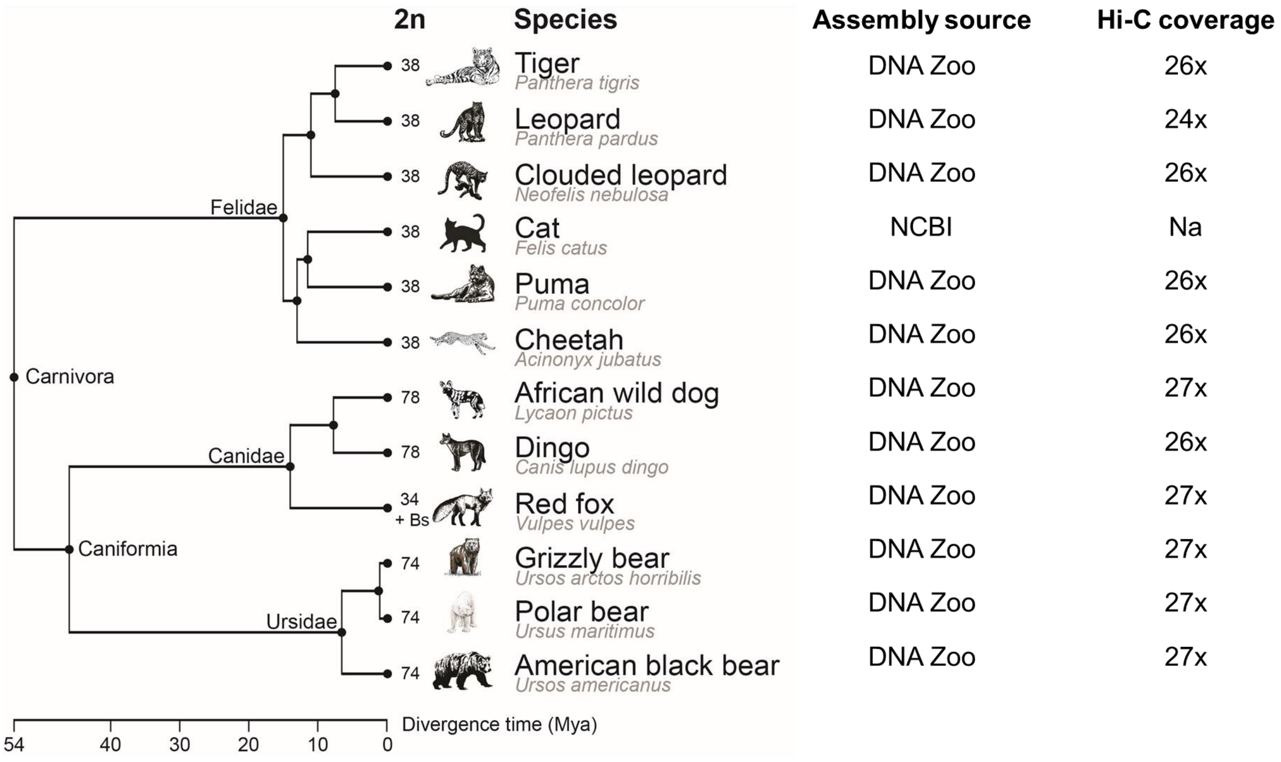

Figure S1. Carnivore phylogenetic tree.

The tree topology for the 12 species and estimated branch lengths were obtained from TimeTree (4). Tree topology was visualized using TreeGraph 2 (5). Species illustrations were obtained from Freepik (https://www.freepik.com/), Vectorstock (https://www.vectorstock.com/), and Shutterstock (https://www.shutterstock.com/). Chromosome numbers were obtained from the animal chromosome count database (6).

FCA A1
242 Mbp

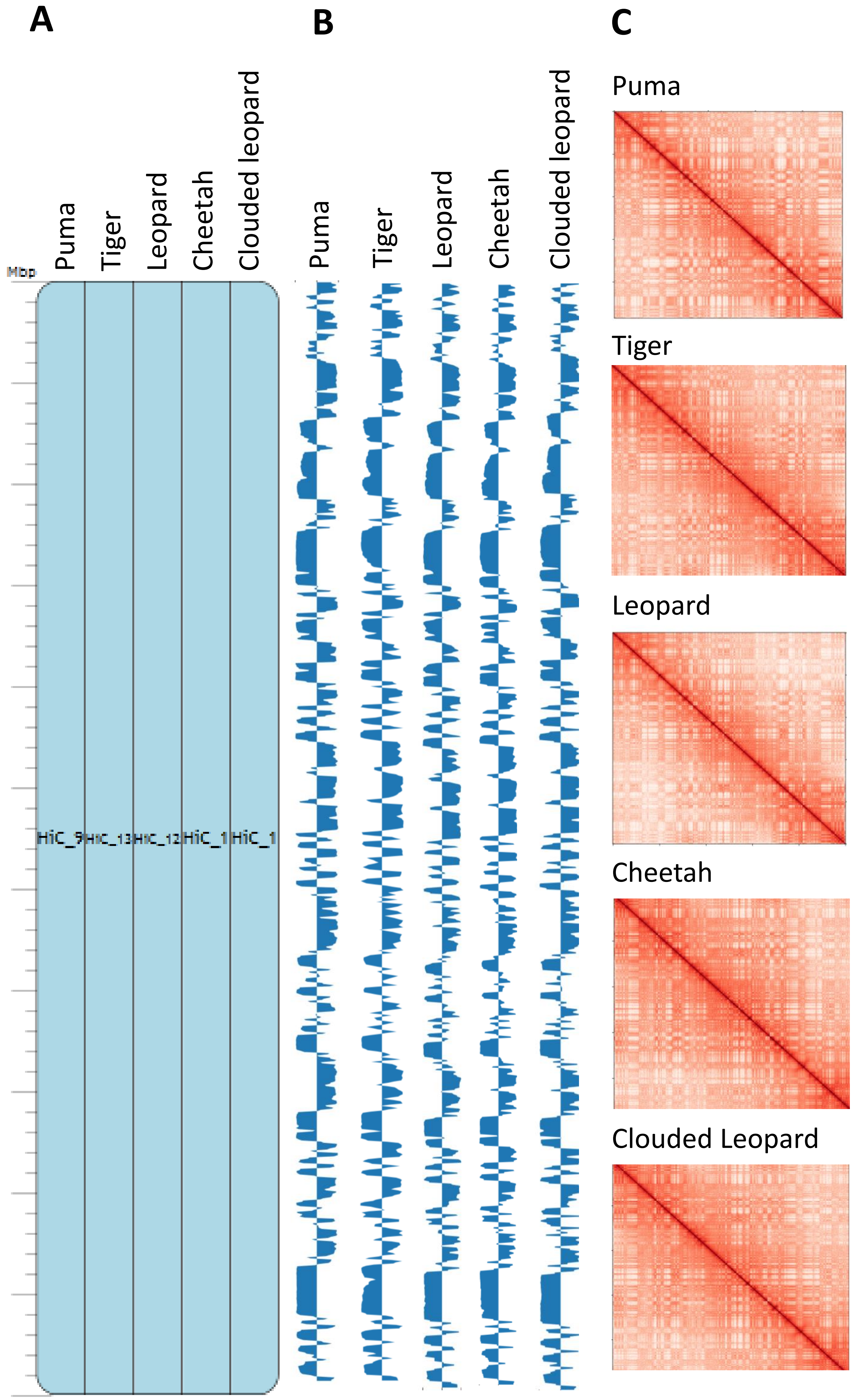

FCA A2

171 Mbp

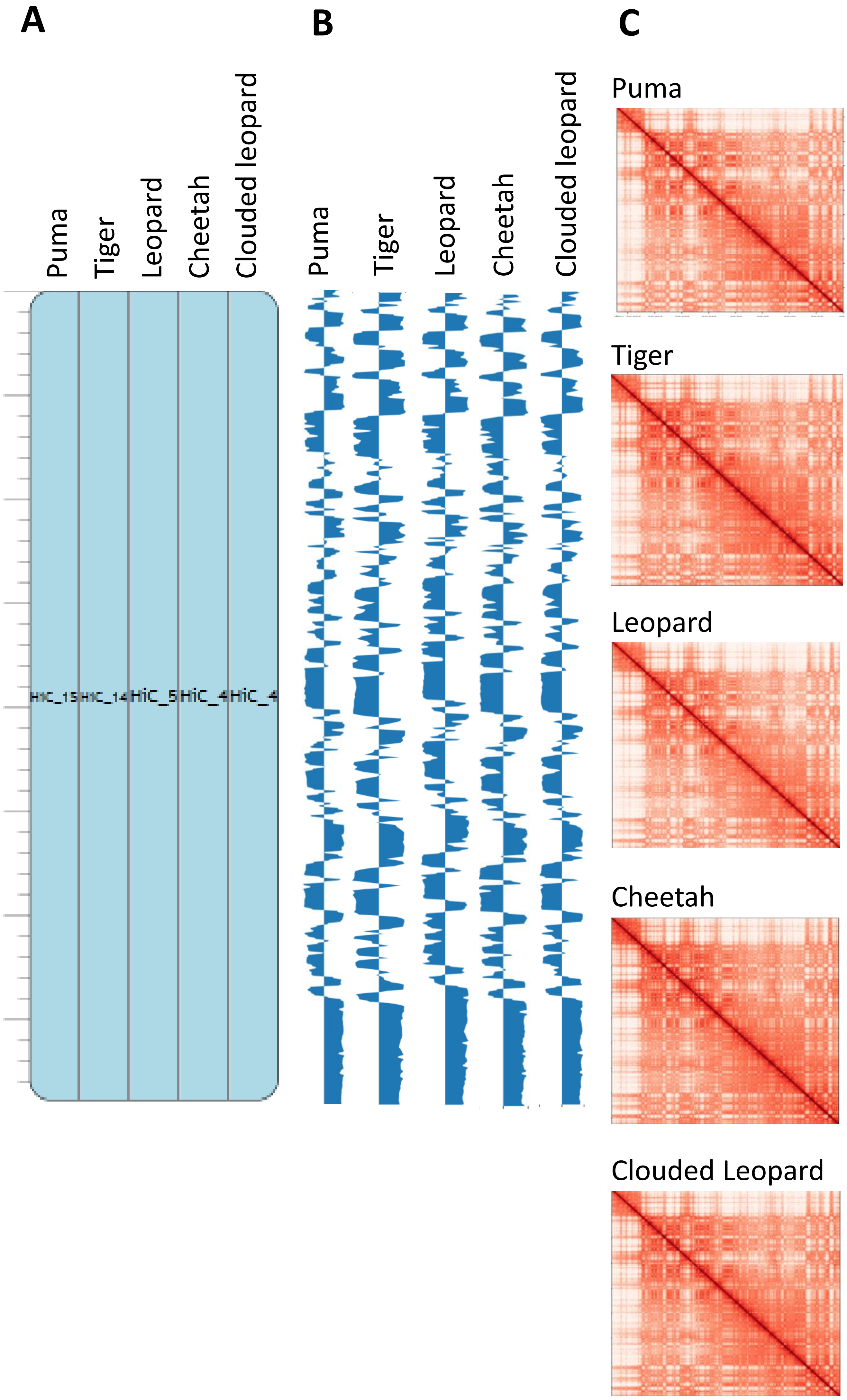

FCA A3
143 Mbp

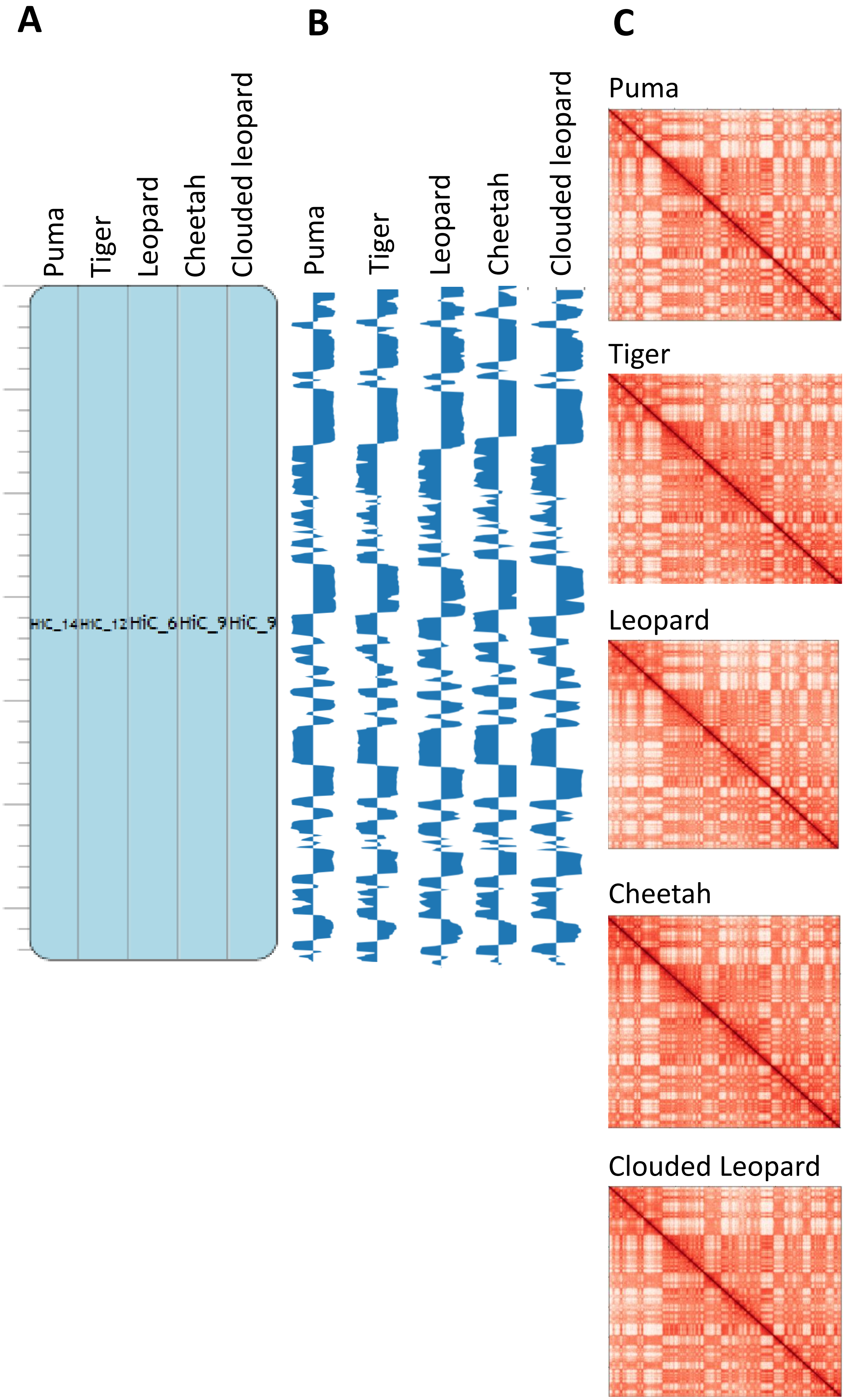

FCA B1
208 Mbp

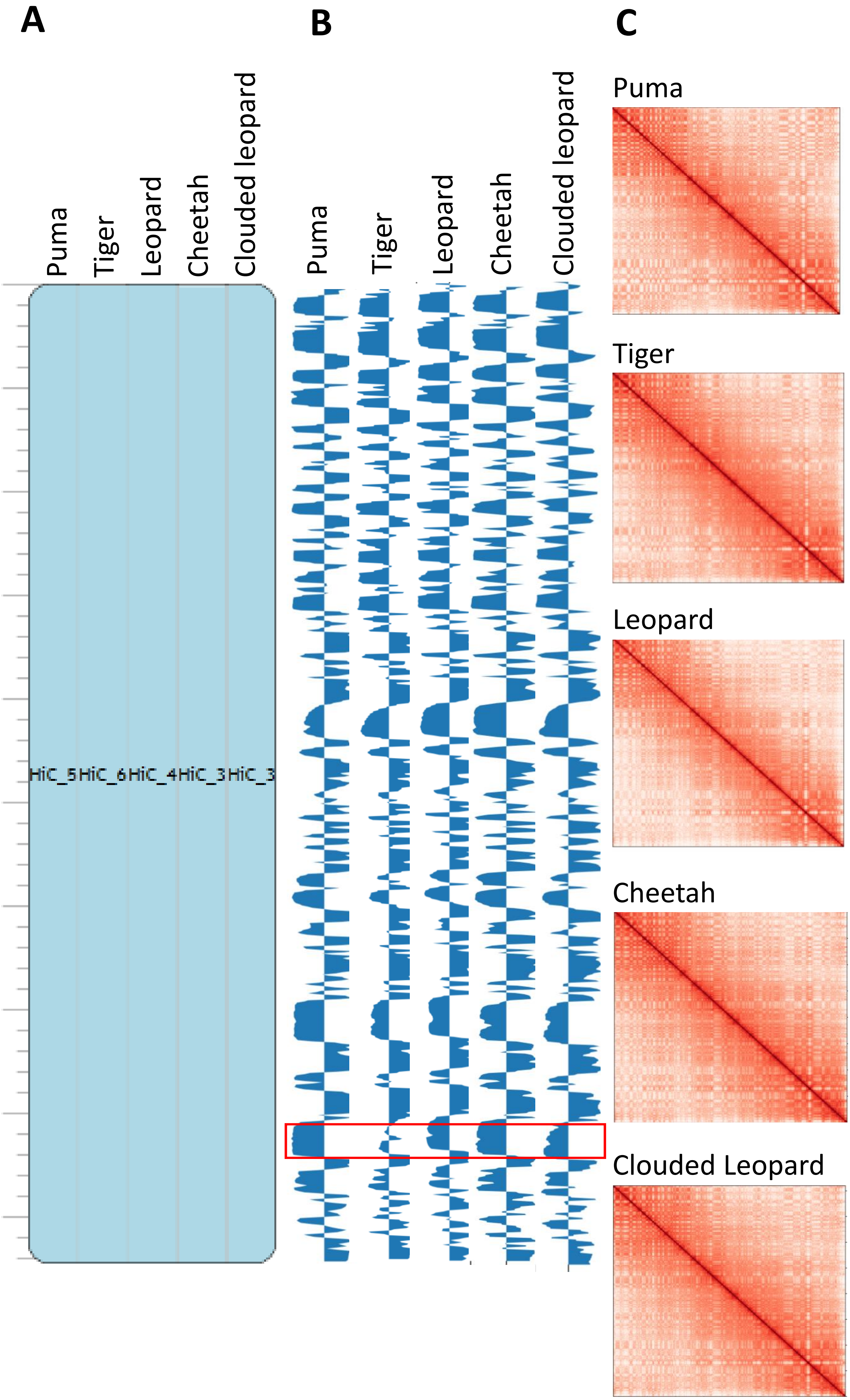

FCA B2

155 Mbp

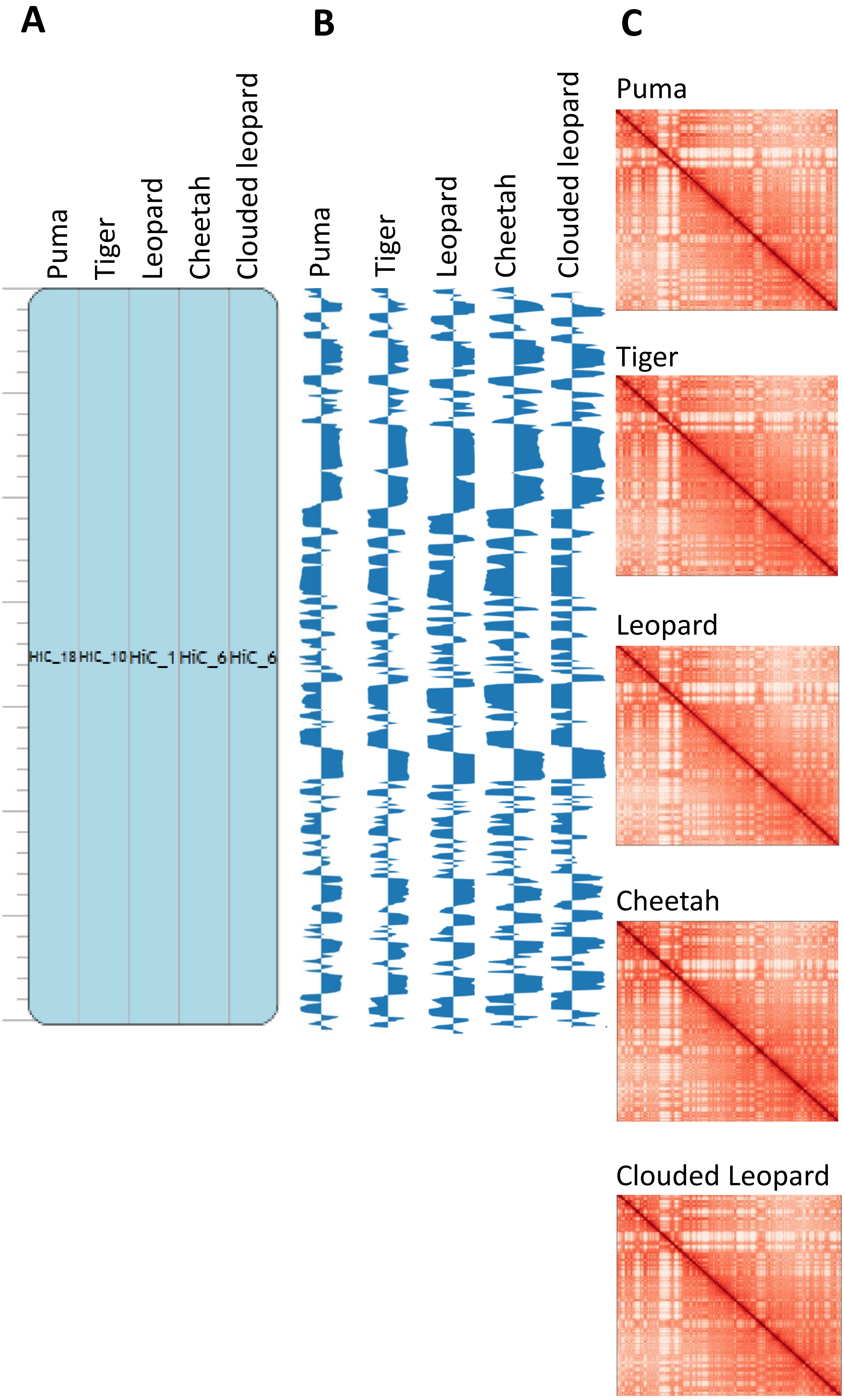

FCA B3

150 Mbp

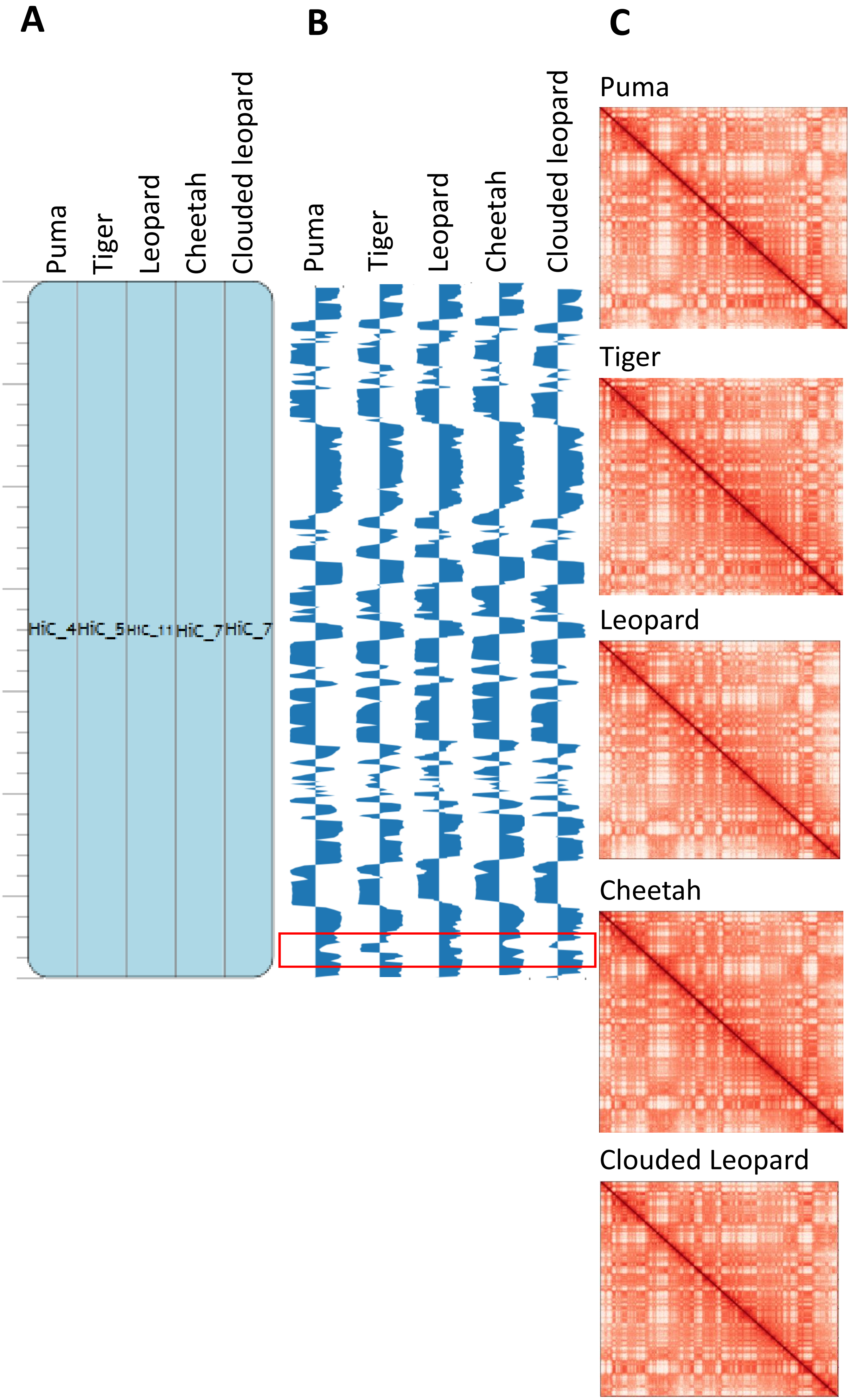

FCA B4

144 Mbp

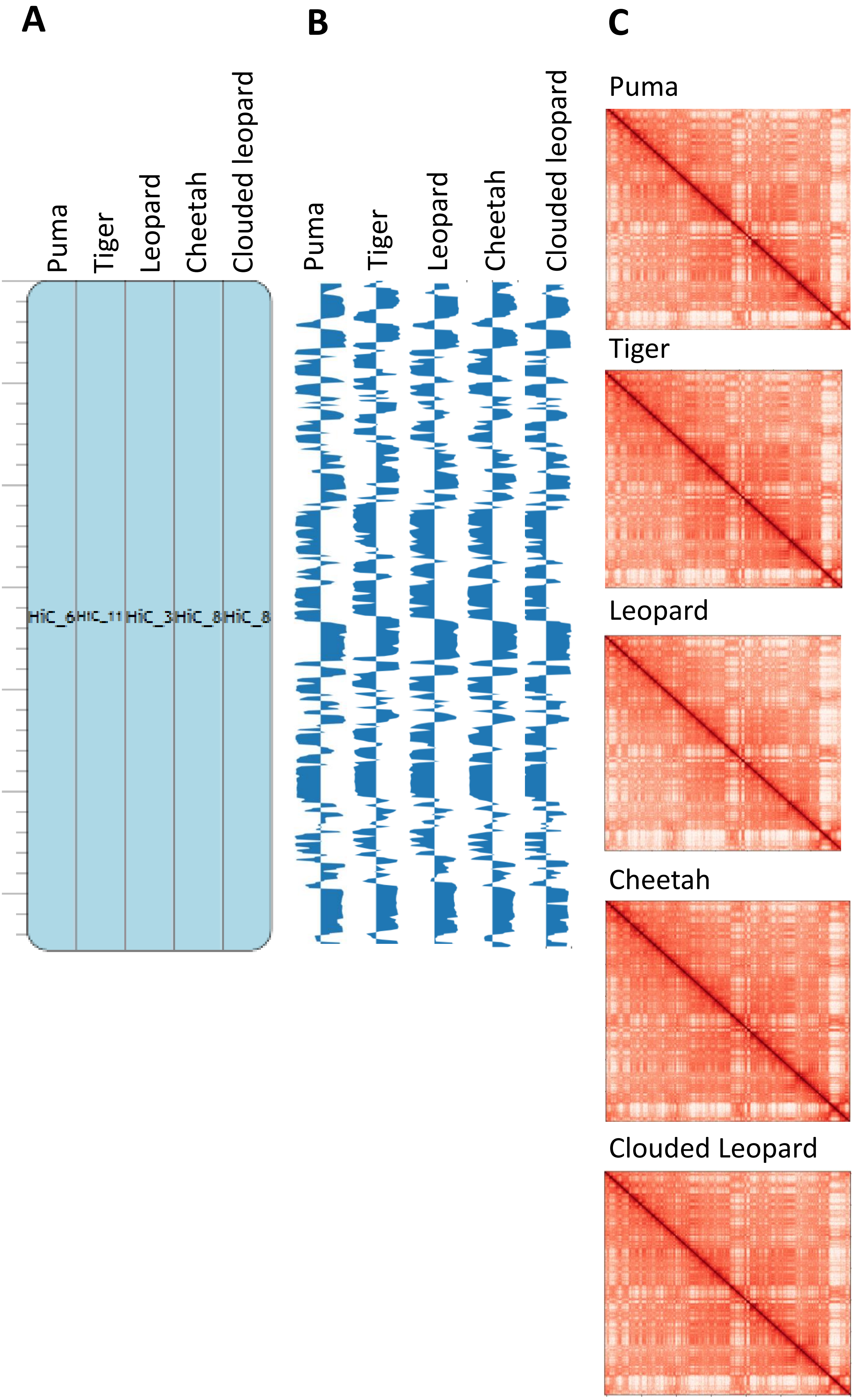

FCA C1

223 Mbp

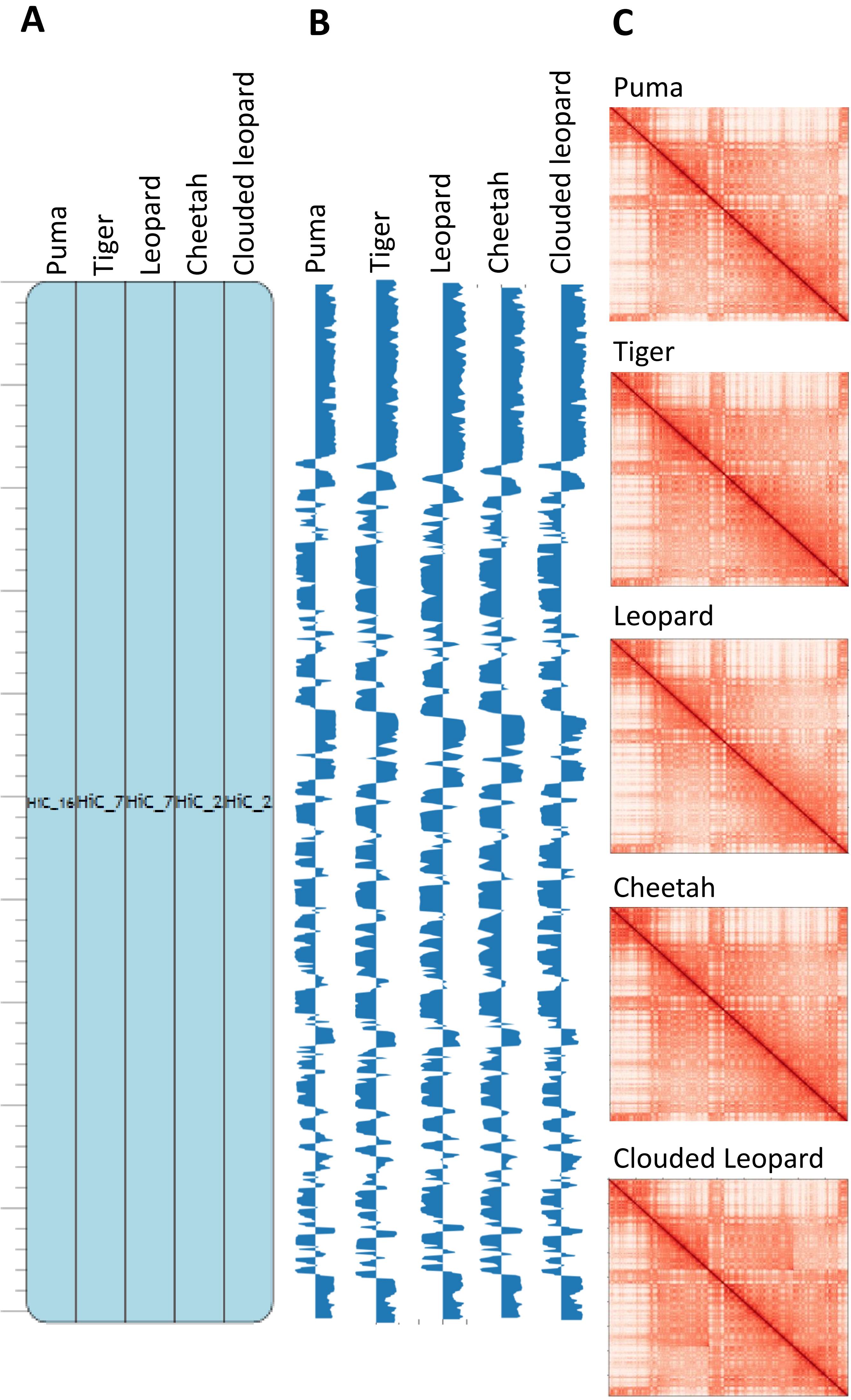

FCA C2
161 Mbp

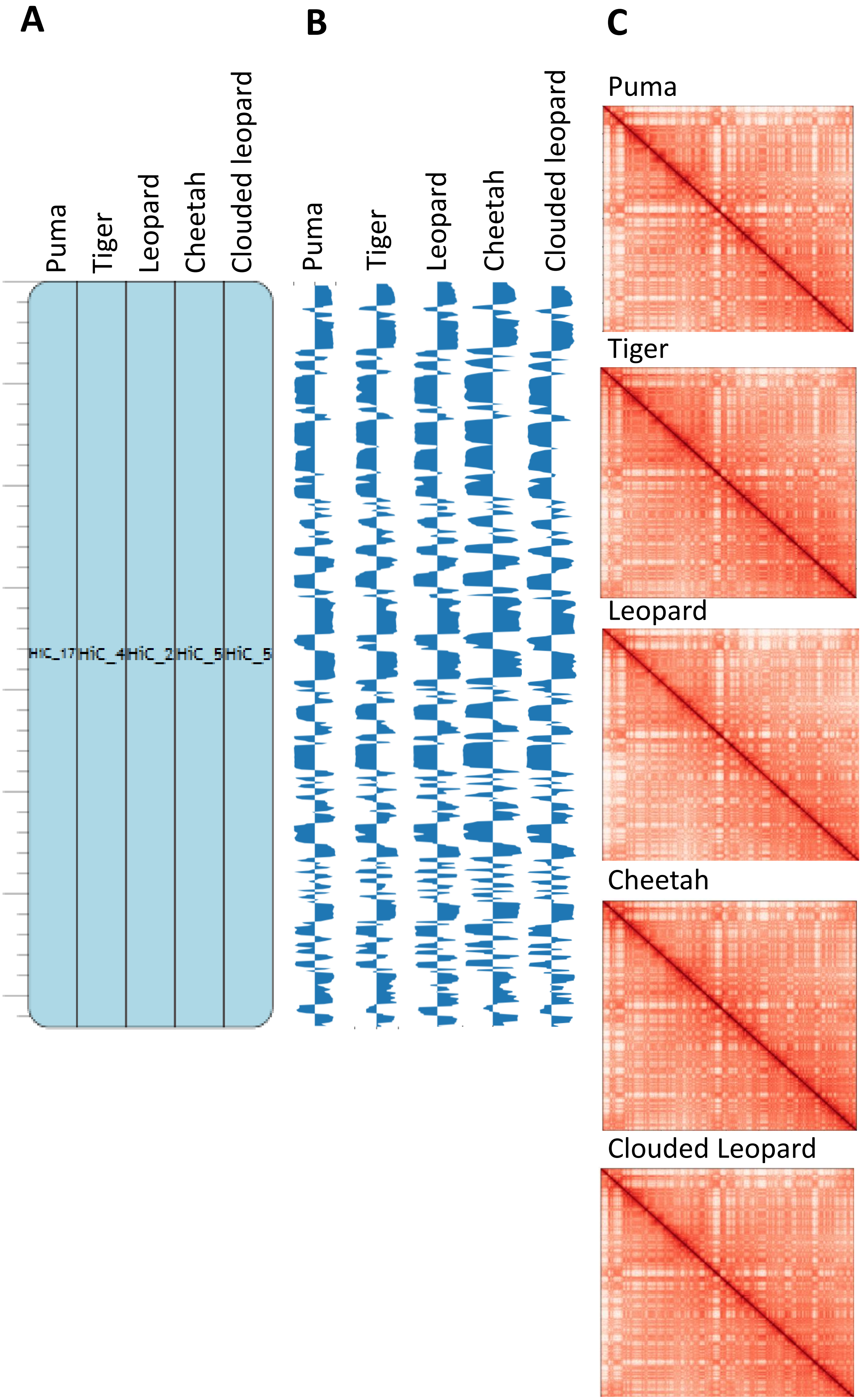

FCA D1

118 Mbp

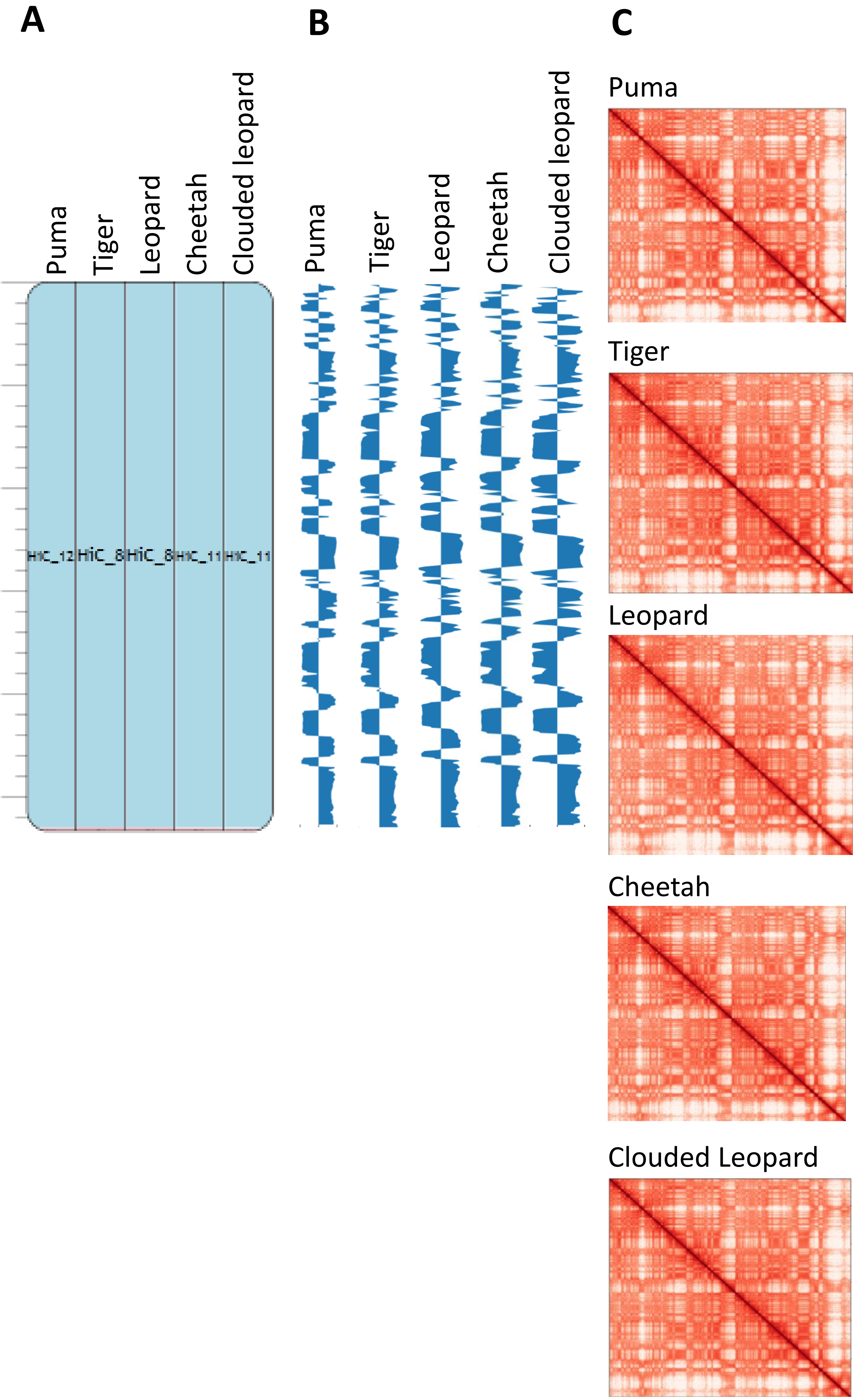

FCA D2

90 Mbp

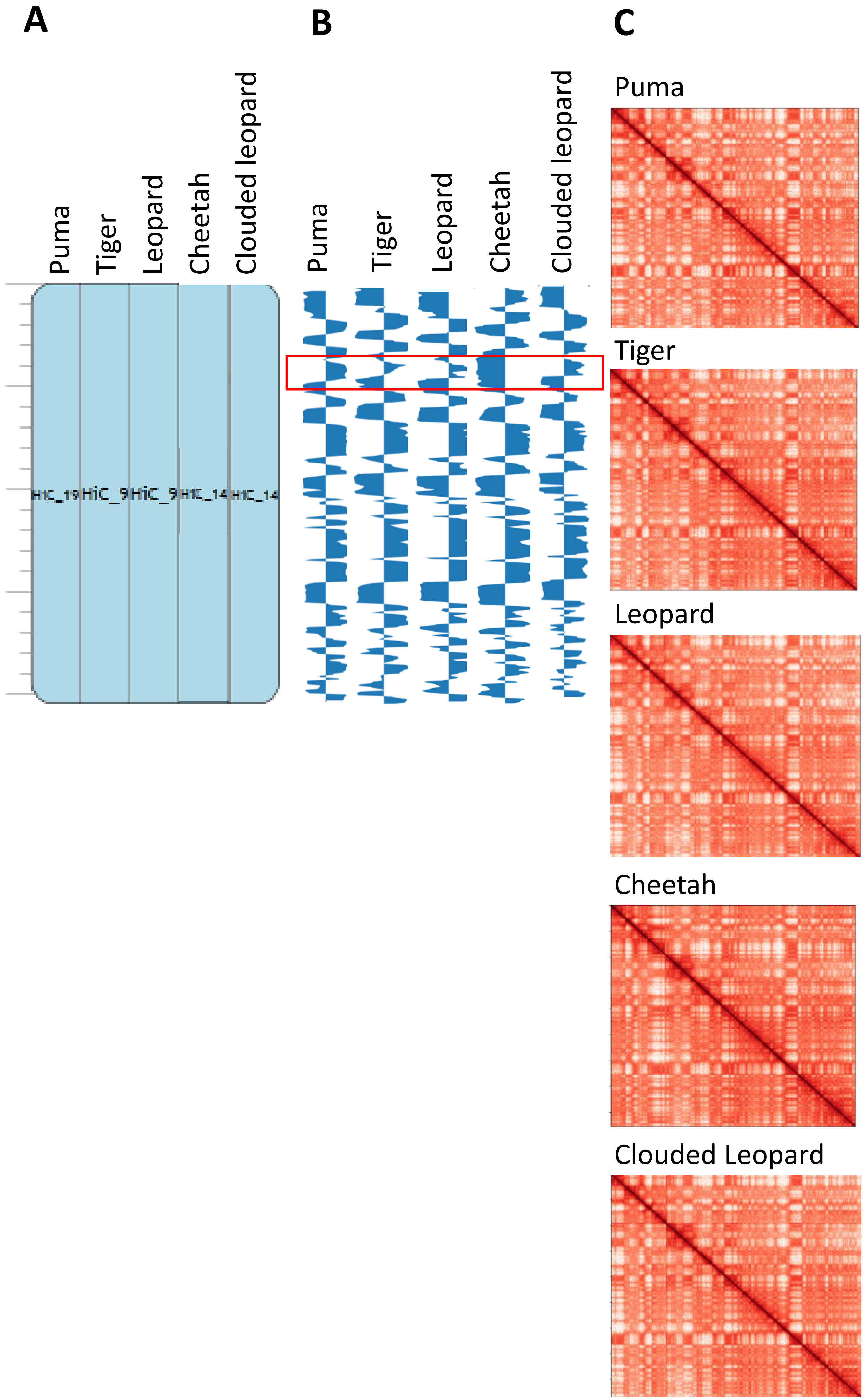
A
B
C
Puma
Tiger
Leopard
Cheetah
Clouded leopard
HiC_19
HiC_9
HiC_9
HiC_14
HiC_14
Puma
Tiger
Leopard
Cheetah
Clouded leopard
Puma
Tiger
Leopard
Cheetah
Clouded Leopard

FCA D4

96 Mbp

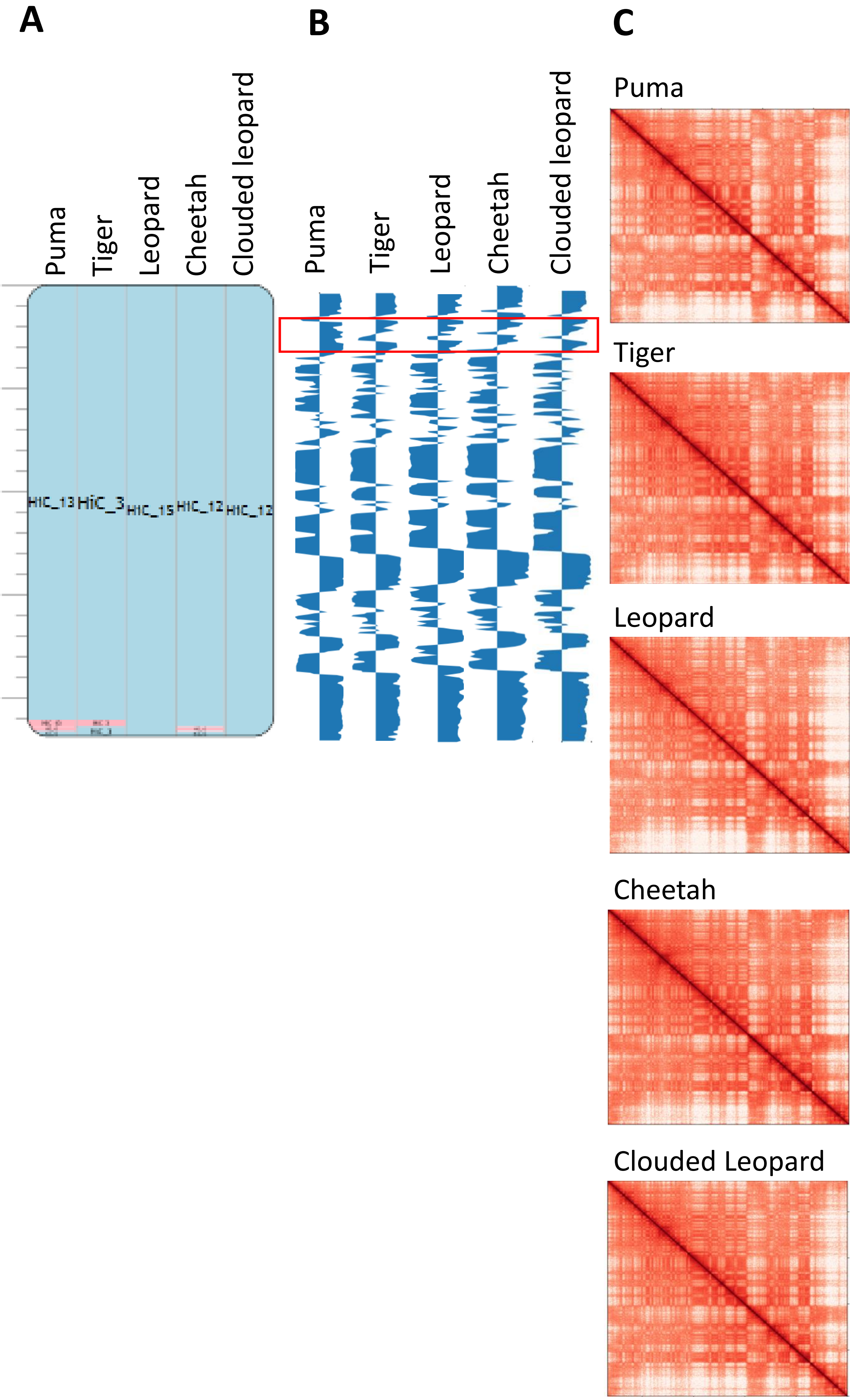
A
B
C
Puma
Tiger
Leopard
Cheetah
Clouded leopard
HiC_13
HiC_3
HiC_15
HiC_12
HiC_12
Puma
Tiger
Leopard
Cheetah
Clouded leopard
Puma
Tiger
Leopard
Cheetah
Clouded Leopard

FCA E2

64 Mbp

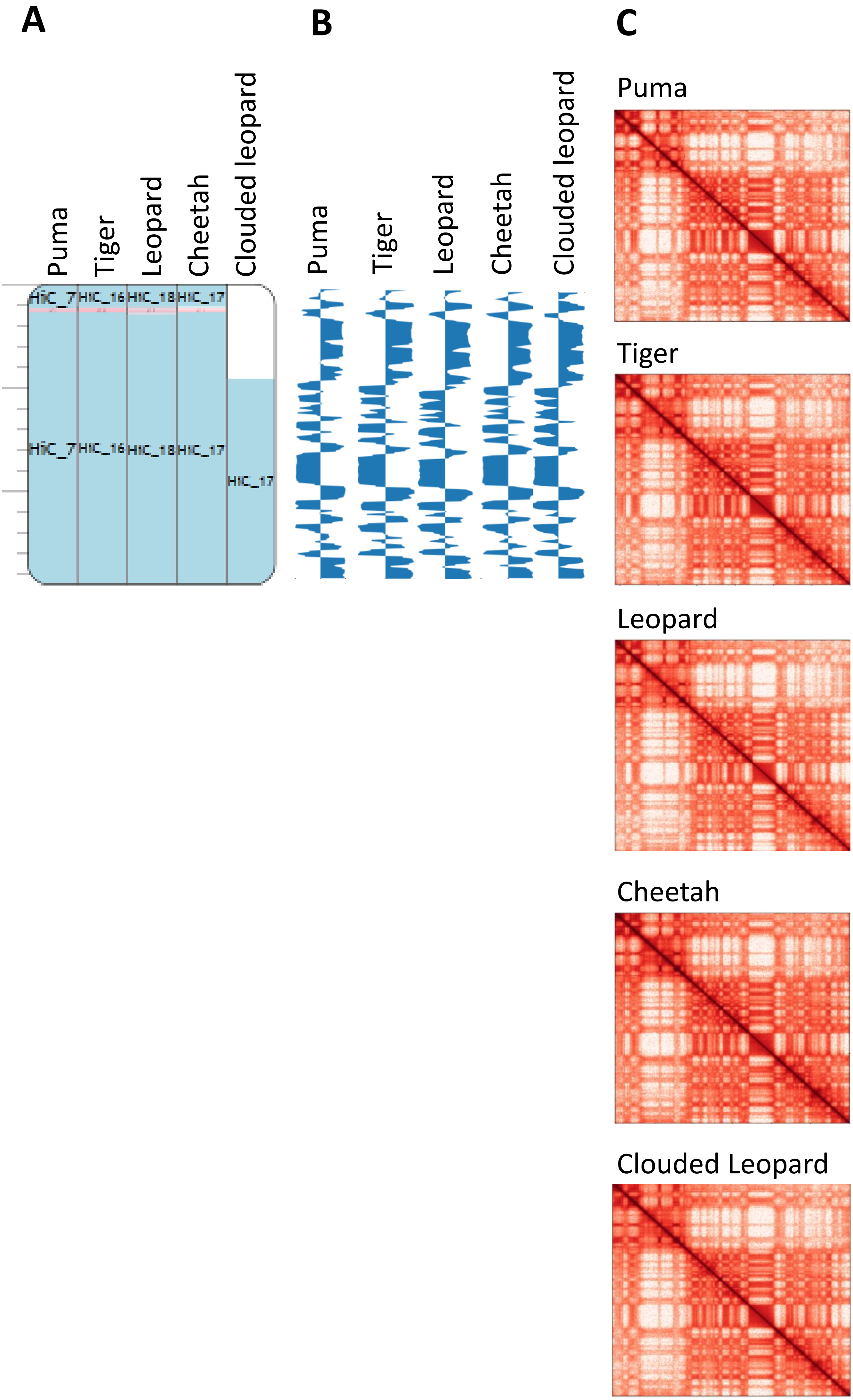
A
B
C
Puma
Tiger
Leopard
Cheetah
Clouded leopard
HiC_7
HiC_16
HiC_18
HiC_17
Puma
Tiger
Leopard
Cheetah
Clouded leopard
Puma
Tiger
Leopard
Cheetah
Clouded Leopard

FCA E3

44 Mbp

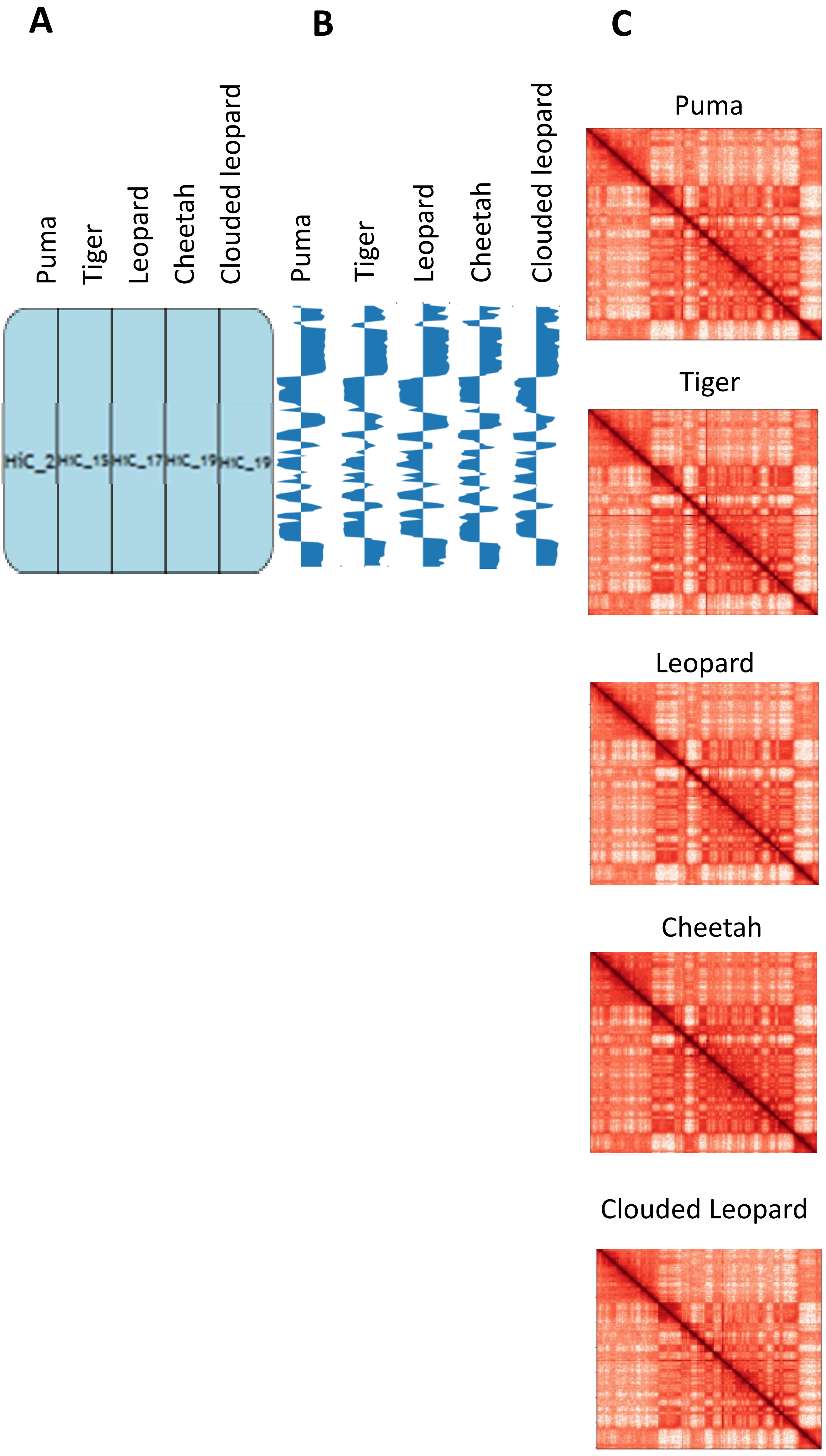
A
B
C
Puma
Tiger
Leopard
Cheetah
Clouded leopard
Puma
Tiger
Leopard
Cheetah
Clouded leopard
Puma
Tiger
Leopard
Cheetah
Clouded Leopard

FCA F1

72 Mbp

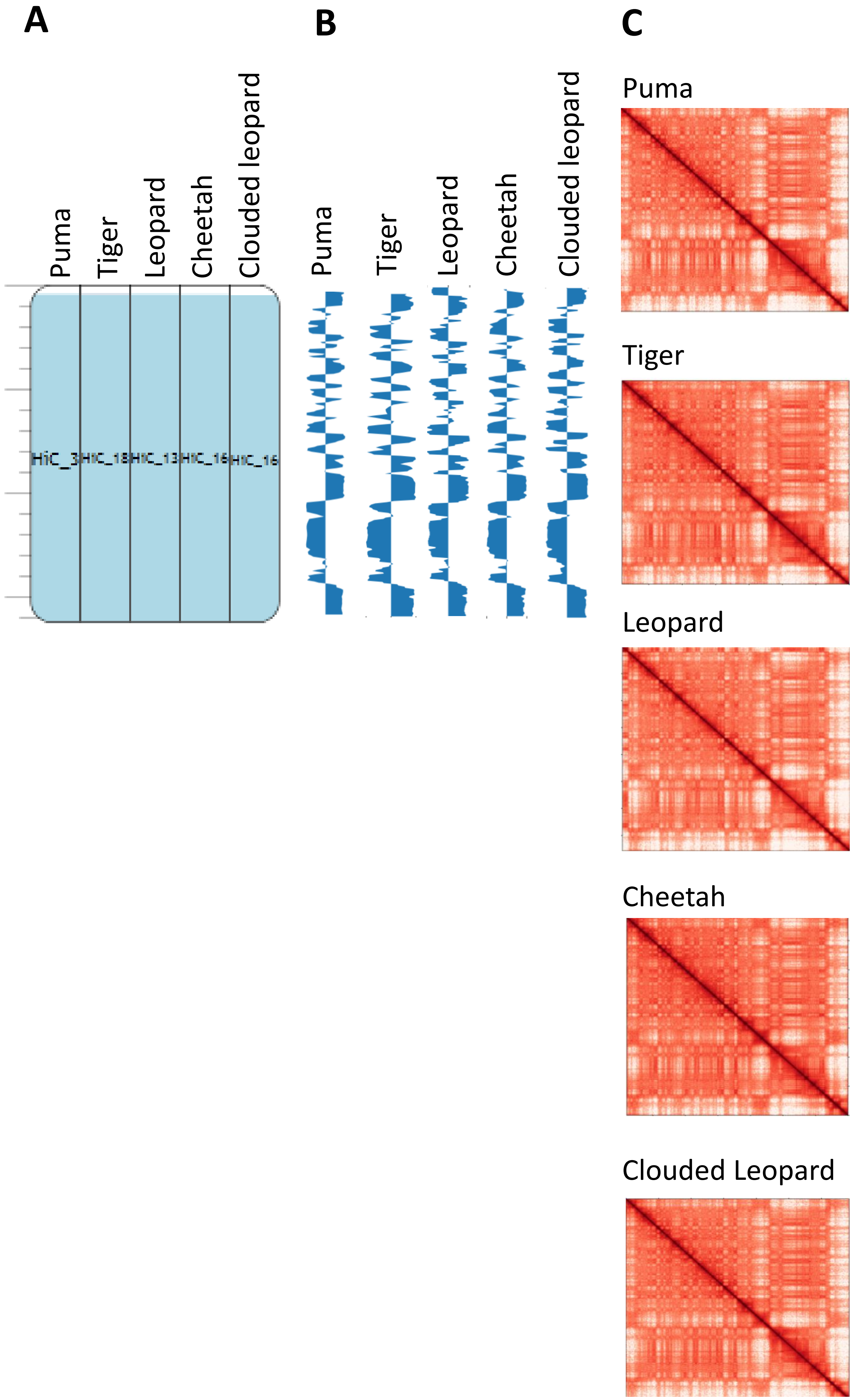
A
B
C
Puma
Tiger
Leopard
Cheetah
Clouded leopard
Puma
Tiger
Leopard
Cheetah
Clouded leopard
Puma
Tiger
Leopard
Cheetah
Clouded Leopard

FCA F2

86 Mbp

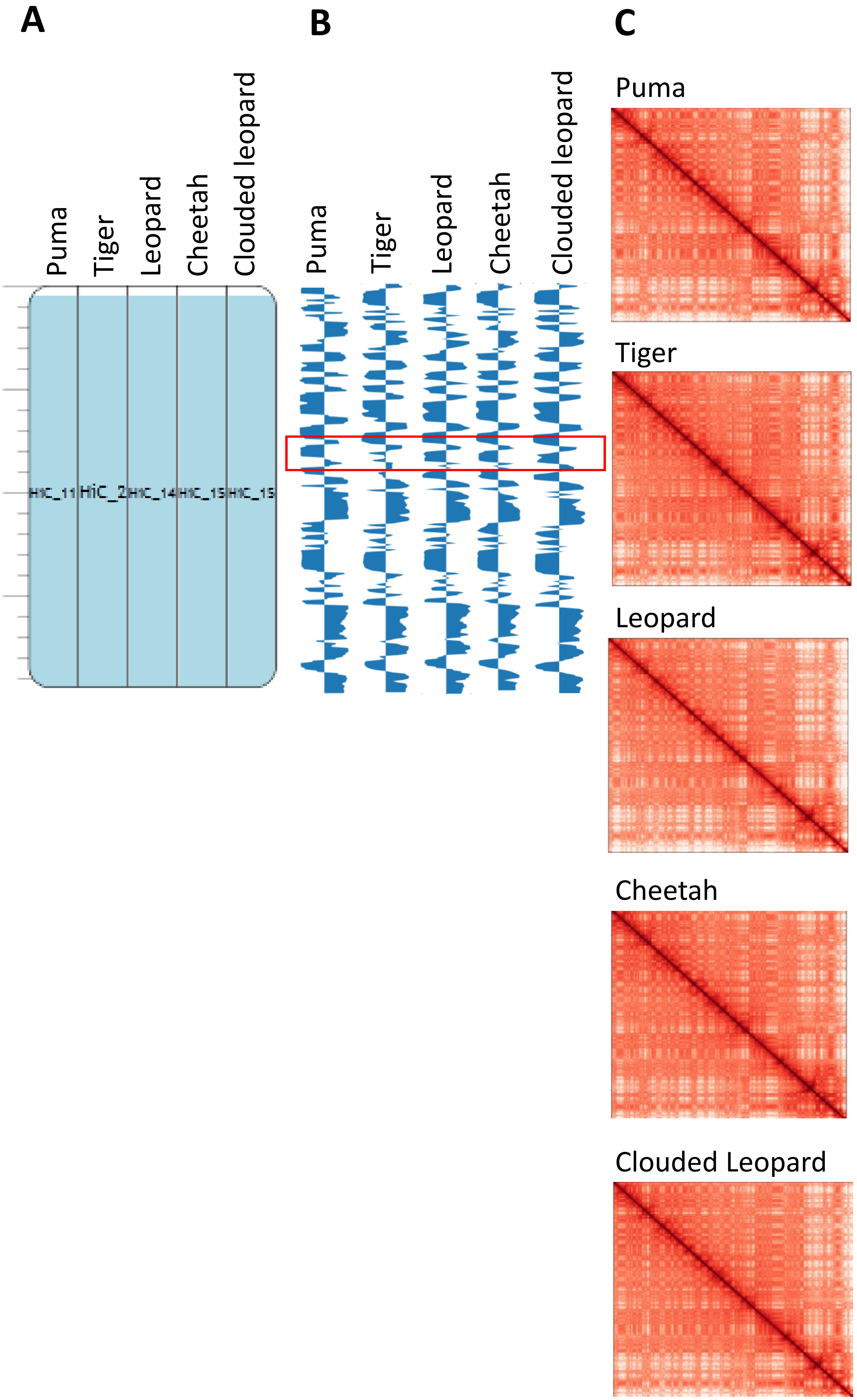
A
B
C
Puma
Tiger
Leopard
Cheetah
Clouded leopard
HiC_2
Puma
Tiger
Leopard
Cheetah
Clouded leopard
Puma
Tiger
Leopard
Cheetah
Clouded Leopard

FCA X

130 Mbp

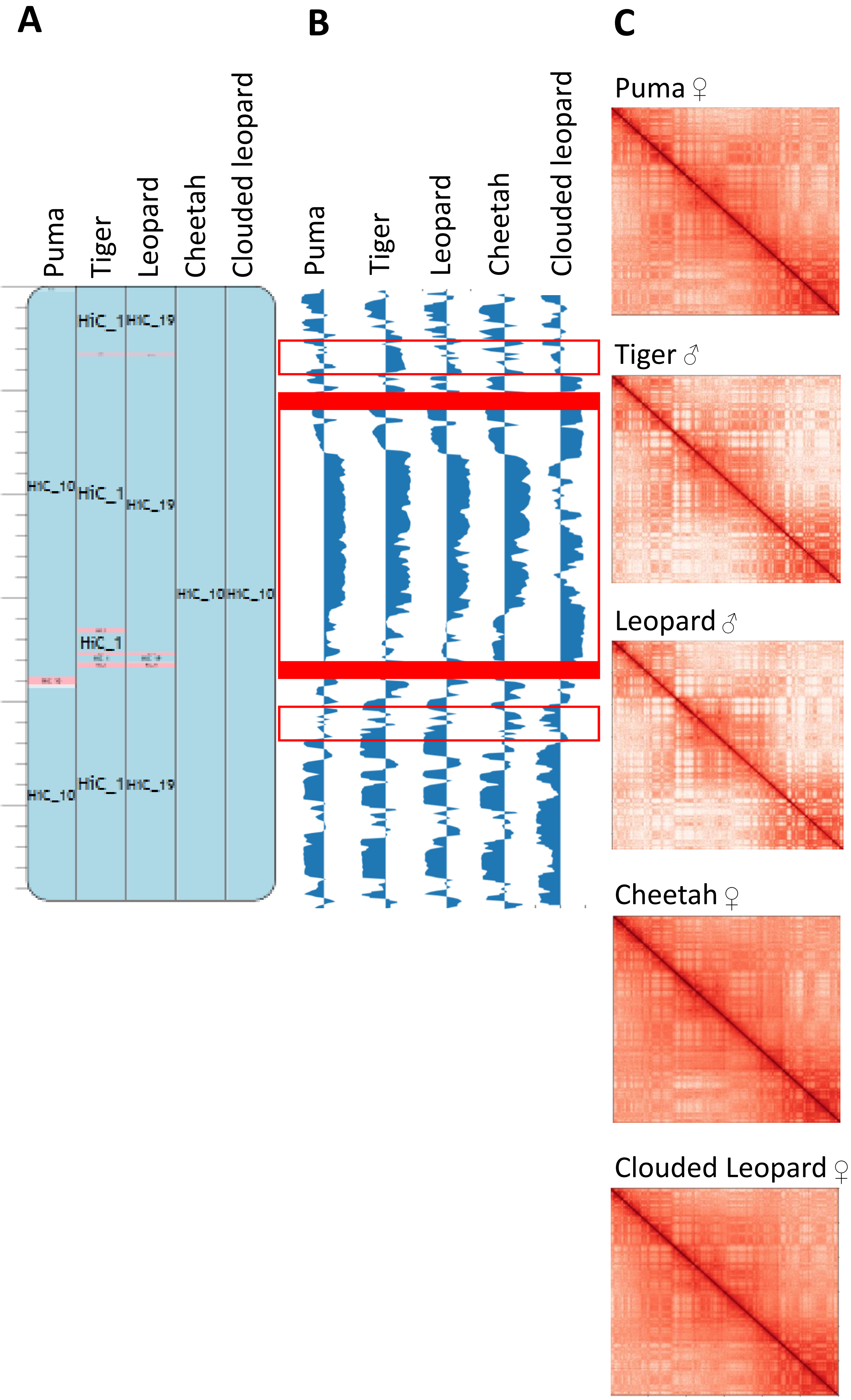

A
B
C
Puma
Tiger
Leopard
Cheetah
Clouded leopard
HiC_10
HiC_1
HiC_19
Puma
Tiger
Leopard
Cheetah
Clouded leopard
Puma ♀
Tiger ♂
Leopard ♂
Cheetah ♀
Clouded Leopard ♀

Figure S2. Comparative chromatin conformation analysis in felids.

Orthologous relationships between puma, tiger, leopard, cheetah, and clouded leopard C-scaffolds and cat chromosomes. (A) Homologous synteny blocks of the three felids visualized in Evolution Highway at 300 kb resolution. Blue indicates same sequence orientation as the reference genome. Pink depicts chromosome inversions. Numbers represent the scaffold identifier in the target species. (B) Eigenvector values of each species aligned to the cat reference genome at 500 kb resolution. Red boxes highlight compartment shifts. (C) Juicer plots of C-scaffolds for the five felid species. Color intensity reflects the frequency of interactions between pairs of loci on the C-scaffolds (range 1-1,000 for each map). Alignment coordinates can be found in SI Dataset S3.

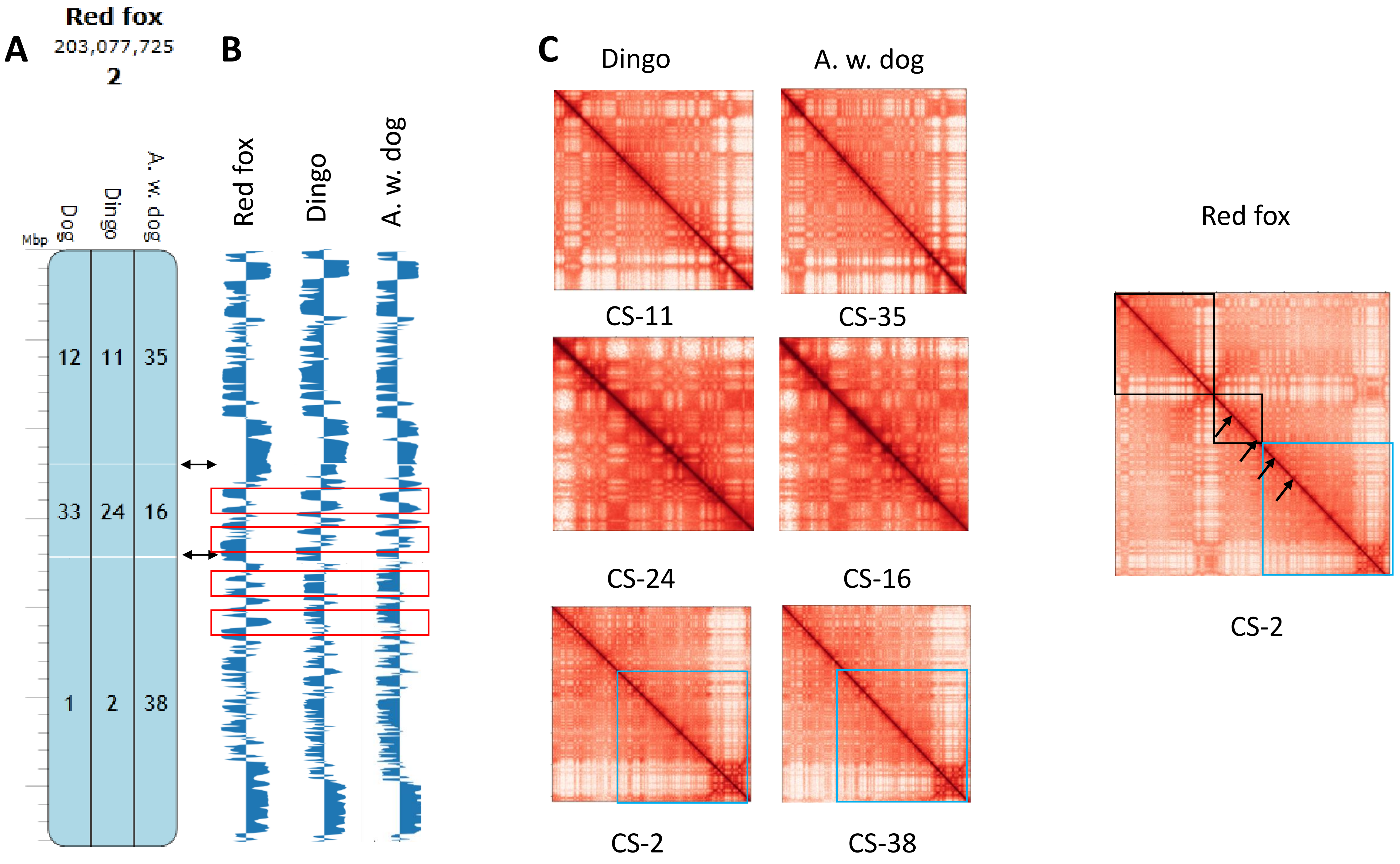
A
Red fox
203,077,725
2
Mbp
Dog
Dingo
A. w. dog
12
11
35
33
24
16
1
2
38
B
Red fox
Dingo
A. w. dog
C
Dingo
A. w. dog
CS-11
CS-35
CS-24
CS-16
CS-2
CS-38
Red fox
CS-2

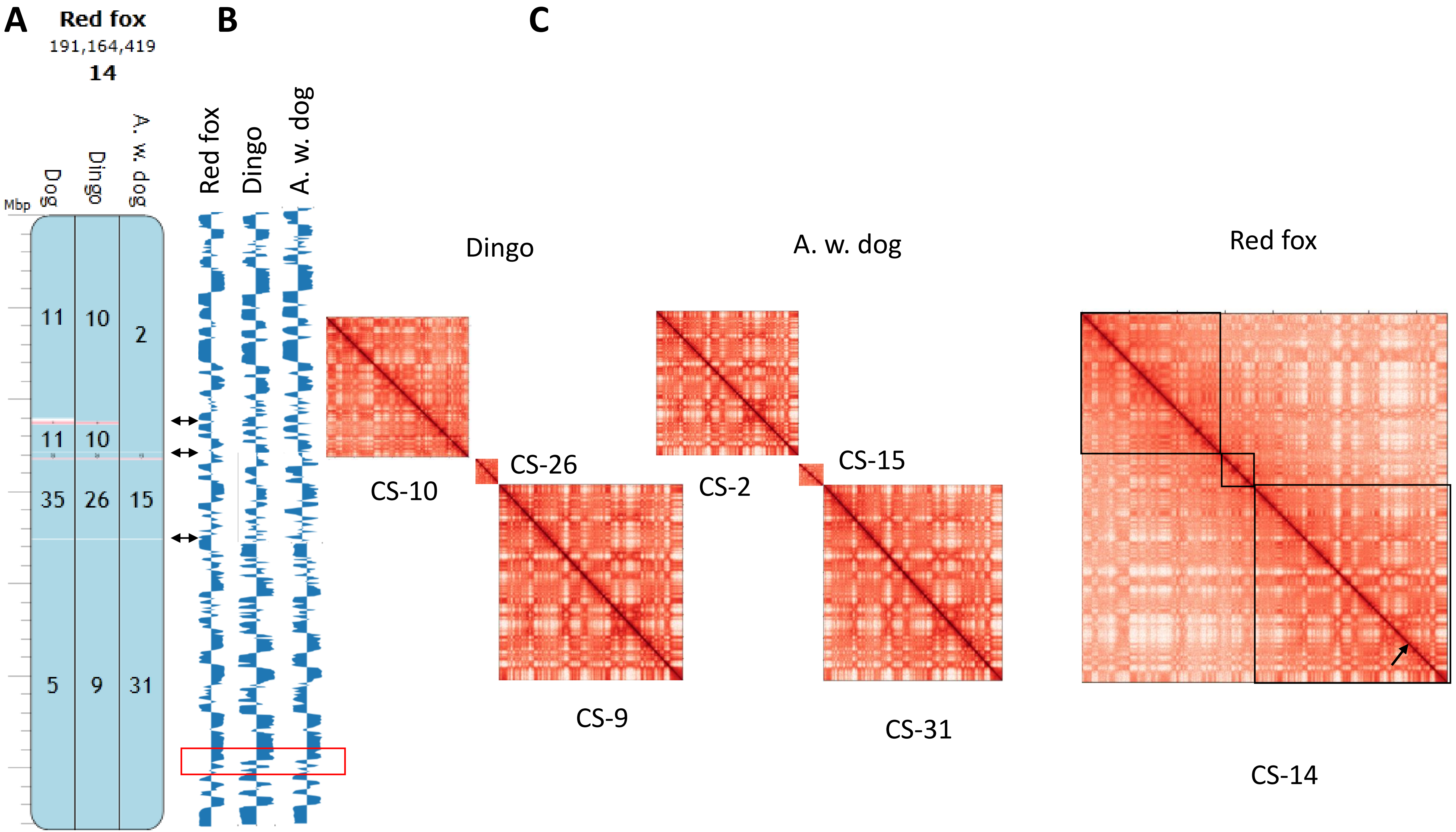
A
Red fox
191,164,419
14
Mbp
Dog
Dingo
A. w. dog
11
10
2
11
10
35
26
15
5
9
31
B
Red fox
Dingo
A. w. dog
C
Dingo
A. w. dog
Red fox
CS-10
CS-26
CS-9
CS-2
CS-15
CS-31
CS-14

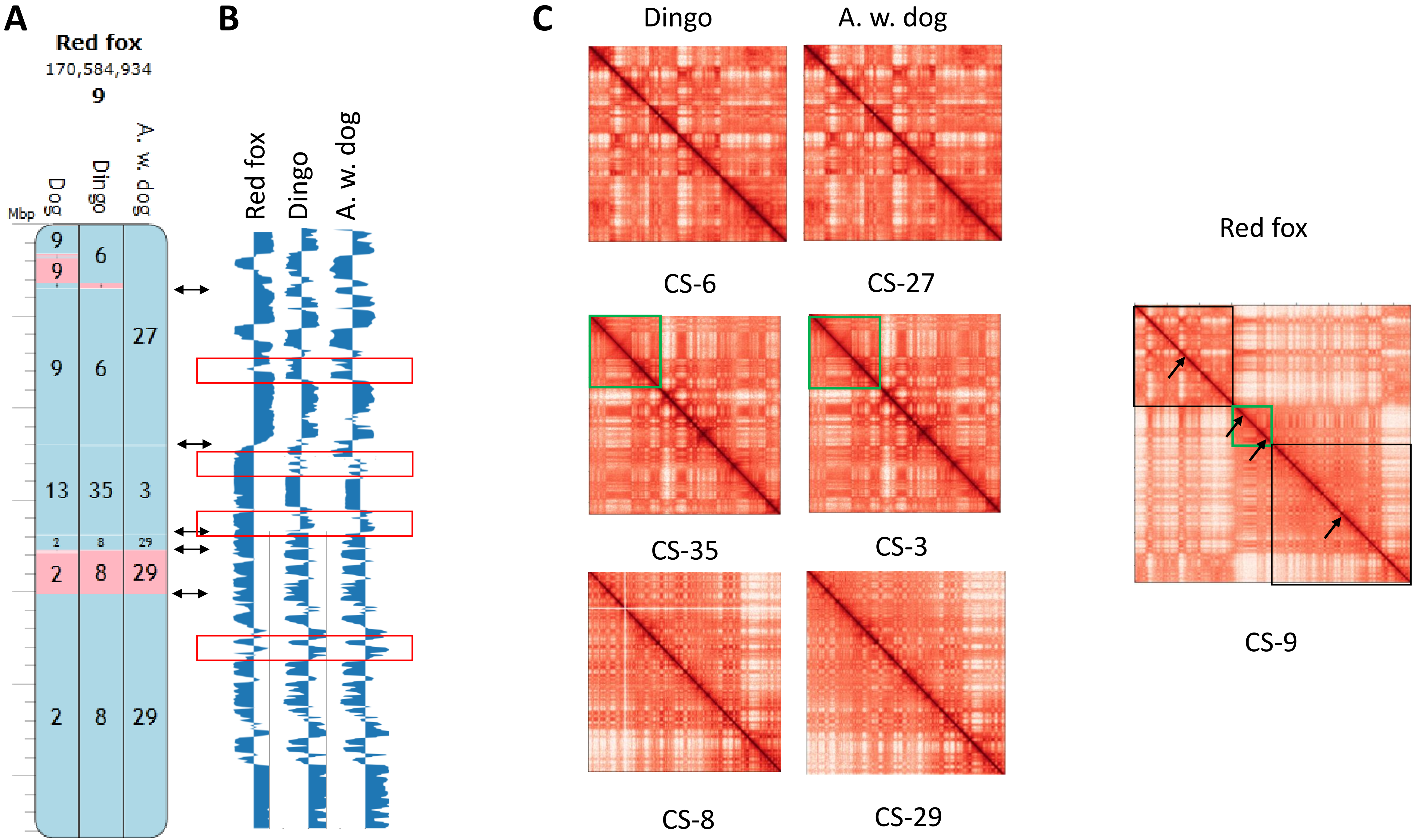
A
Red fox
170,584,934
9
Mbp
Dog
Dingo
A. w. dog
9
9
6
27
9
6
13
35
3
2
8
29
2
8
29
2
8
29
B
Red fox
Dingo
A. w. dog
C
Dingo
A. w. dog
CS-6
CS-27
CS-35
CS-3
CS-8
CS-29
Red fox
CS-9

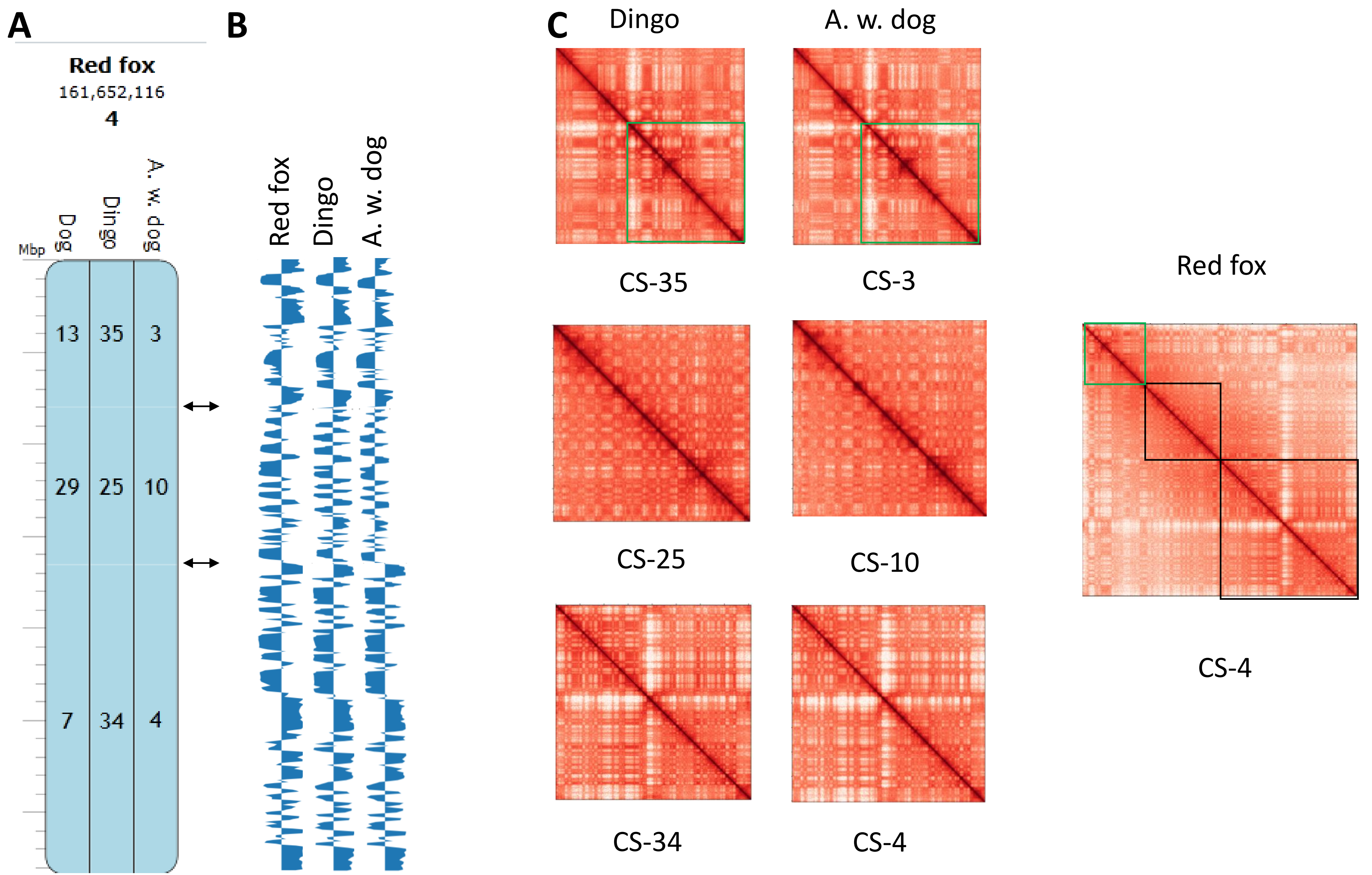

A
Red fox
161,652,116
4
Mbp
Dog
Dingo
A. w. dog
13
35
3
29
25
10
7
34
4
B
Red fox
Dingo
A. w. dog
C
Dingo
A. w. dog
CS-35
CS-3
CS-25
CS-10
CS-34
CS-4
Red fox
CS-4

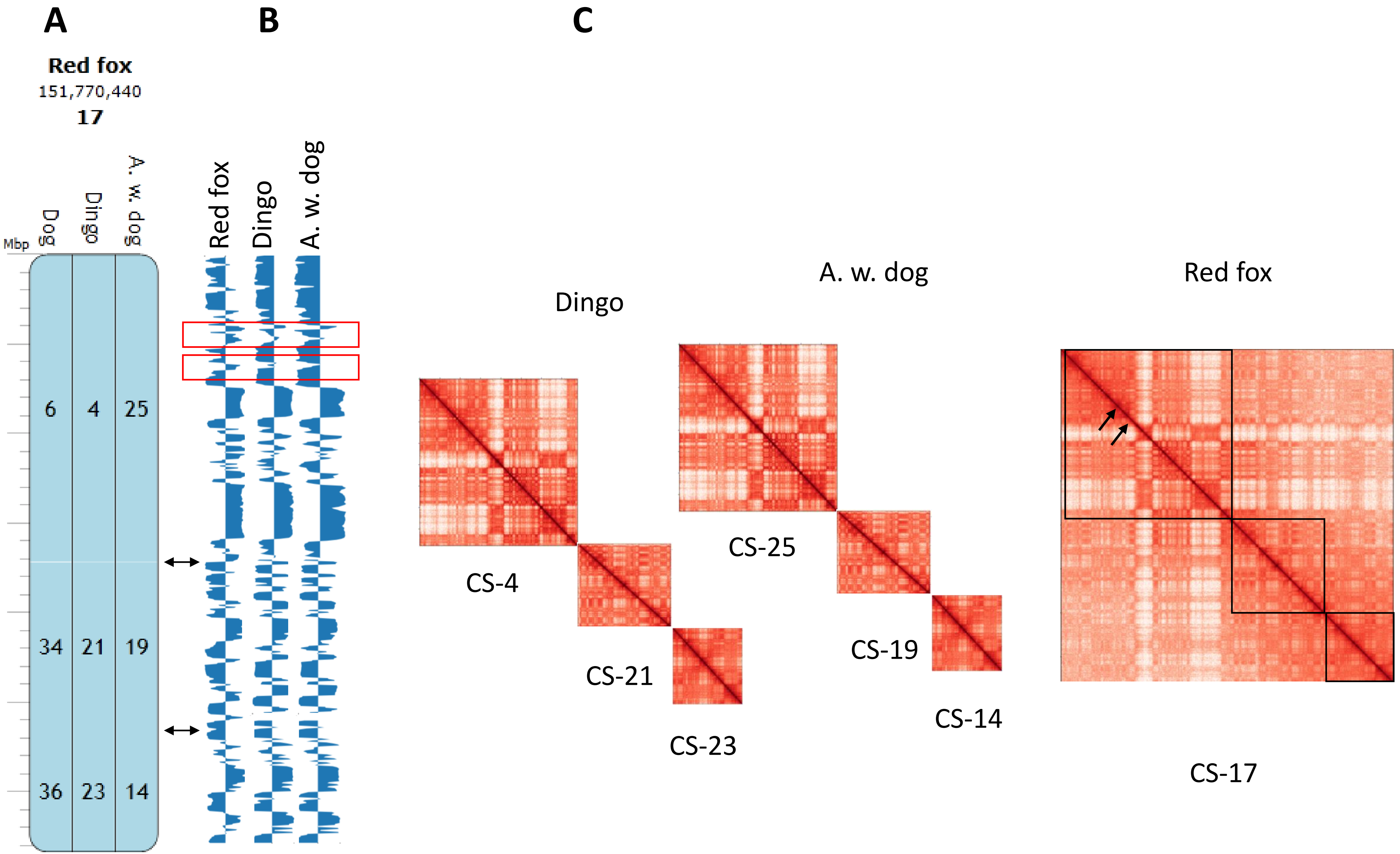
A
B
C
Red fox
151,770,440
17
Mbp
Dog
Dingo
A. w. dog
6
4
25
34
21
19
36
23
14
Red fox
Dingo
A. w. dog
Dingo
A. w. dog
Red fox
CS-4
CS-21
CS-23
CS-25
CS-19
CS-14
CS-17

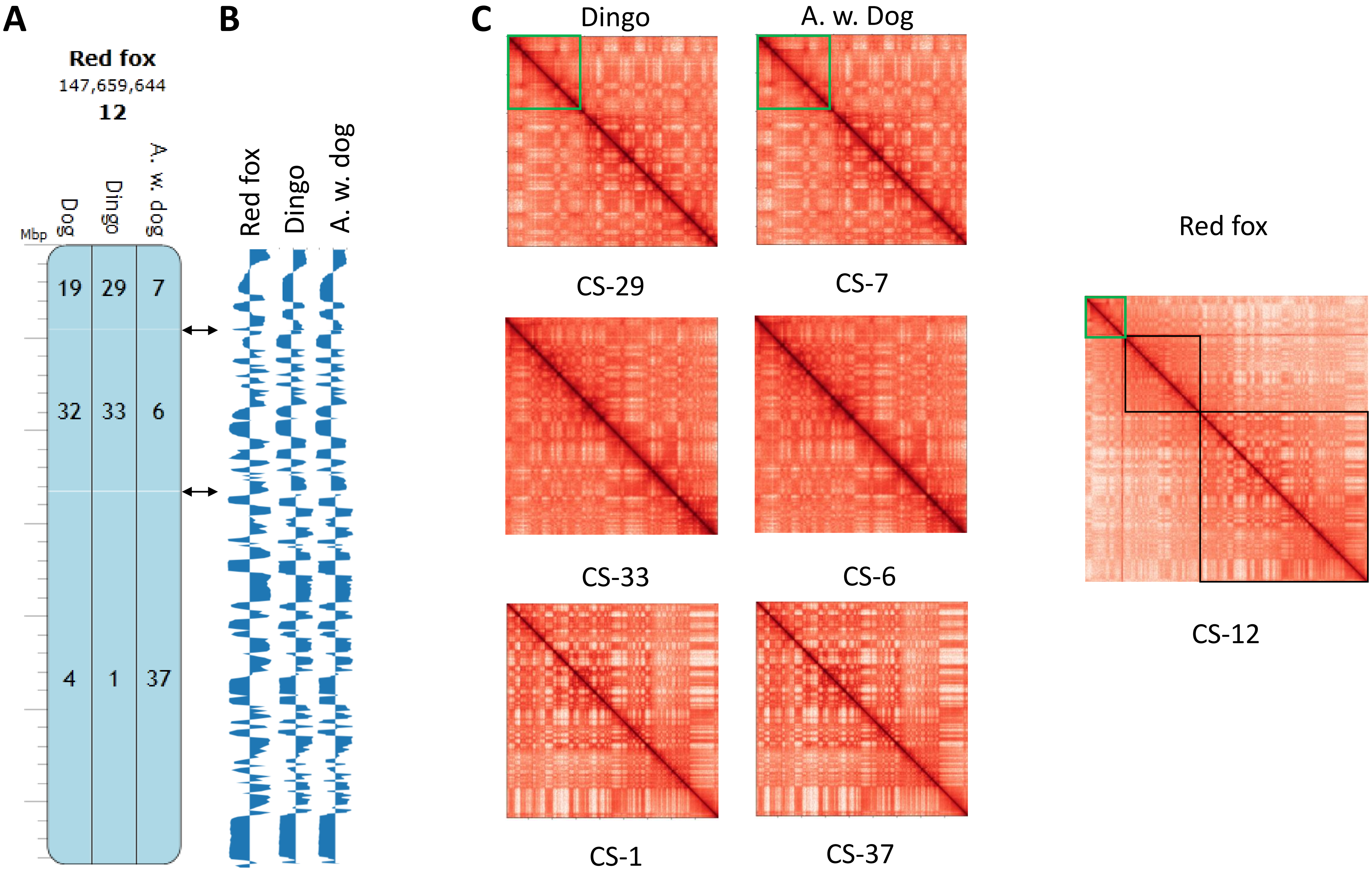
A
Red fox
147,659,644
12
Mbp
Dog
Dingo
A. w. dog
19
29
7
32
33
6
4
1
37
B
Red fox
Dingo
A. w. dog
C
Dingo
A. w. Dog
CS-29
CS-7
CS-33
CS-6
CS-1
CS-37
Red fox
CS-12

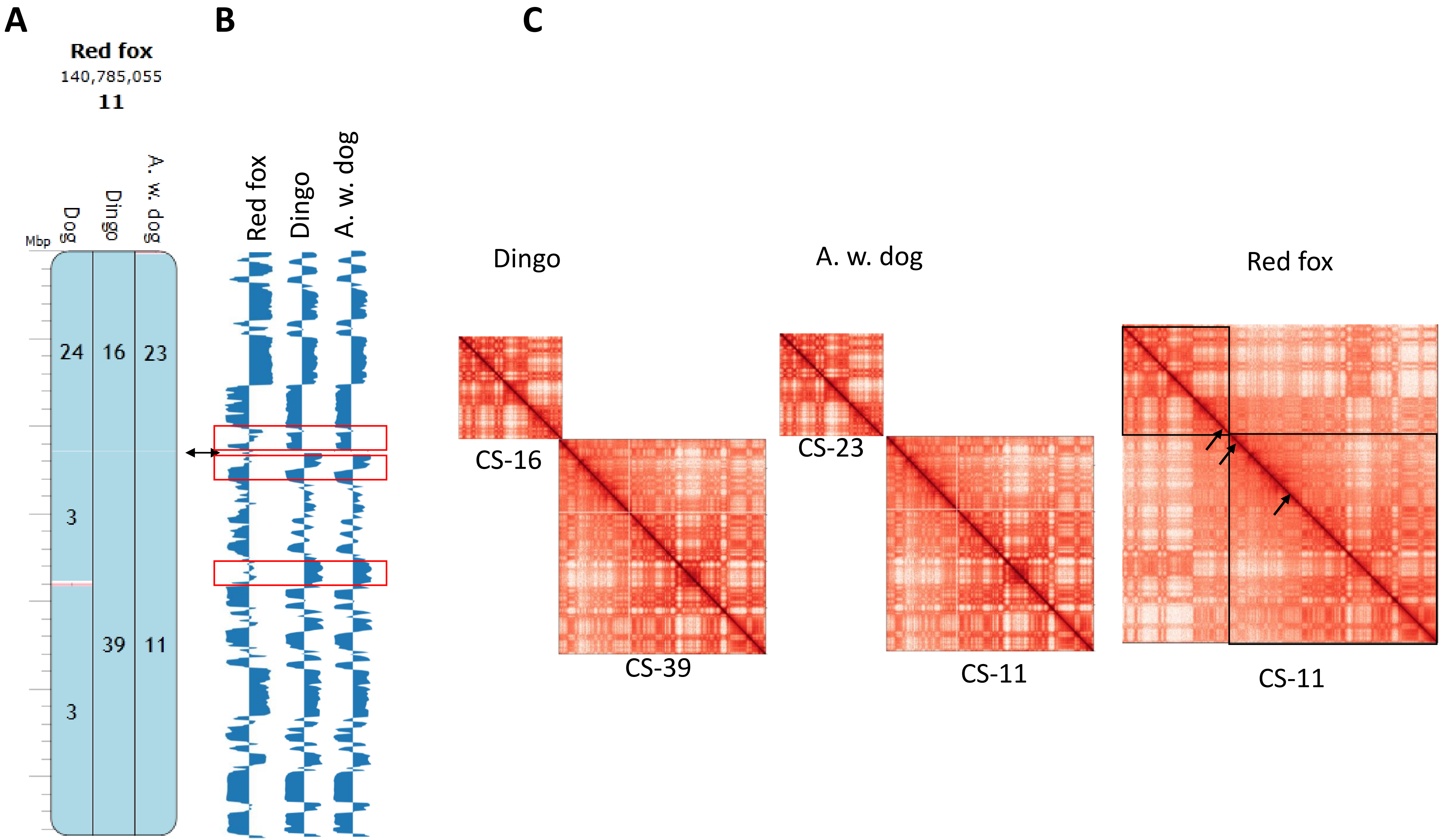
A
Red fox
140,785,055
11
Mbp
Dog
Dingo
A. w. dog
24
16
23
3
39
11
3
B
Red fox
Dingo
A. w. dog
C
Dingo
A. w. dog
Red fox
CS-16
CS-39
CS-23
CS-11
CS-11

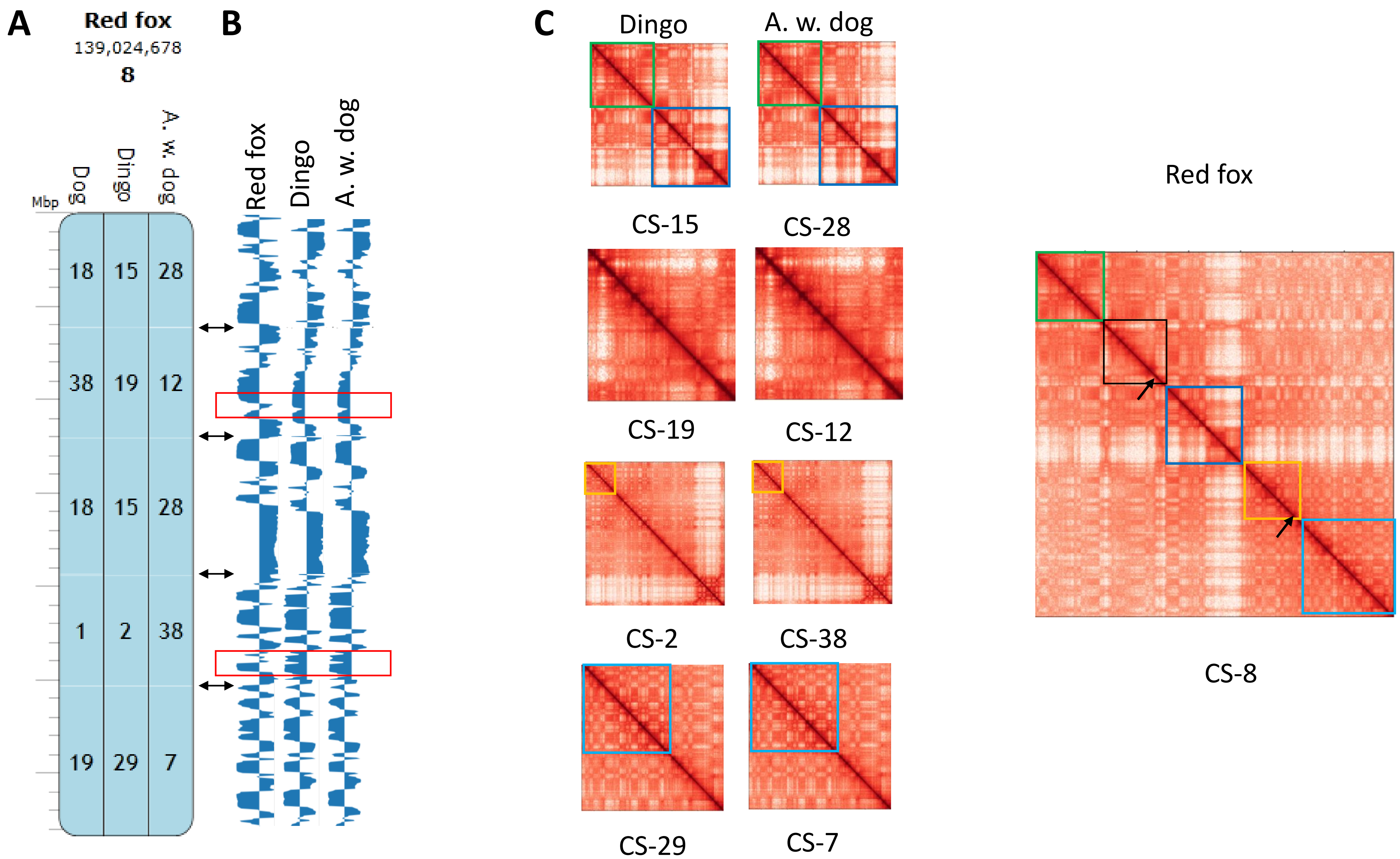

A
Red fox
139,024,678
8
Mbp
Dog
Dingo
A. w. dog
18 15 28
38 19 12
18 15 28
1 2 38
19 29 7
B
Red fox
Dingo
A. w. dog
C
Dingo
A. w. dog
CS-15
CS-28
CS-19
CS-12
CS-2
CS-38
CS-29
CS-7
Red fox
CS-8

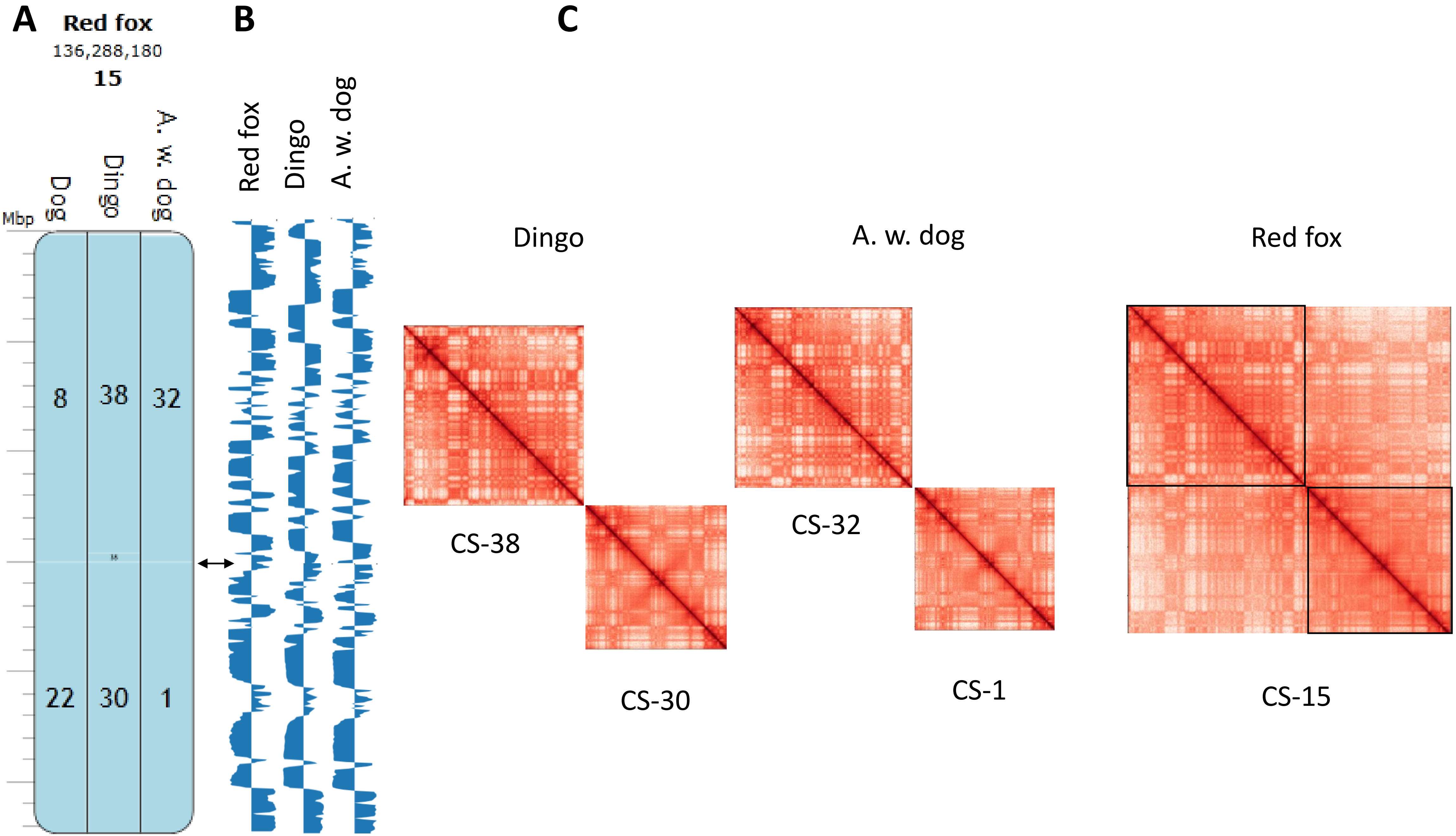
A
Red fox
136,288,180
15
Mbp
Dog
Dingo
A. w. dog
8
38
32
22
30
1
B
Red fox
Dingo
A. w. dog
C
Dingo
A. w. dog
Red fox
CS-38
CS-30
CS-32
CS-1
CS-15

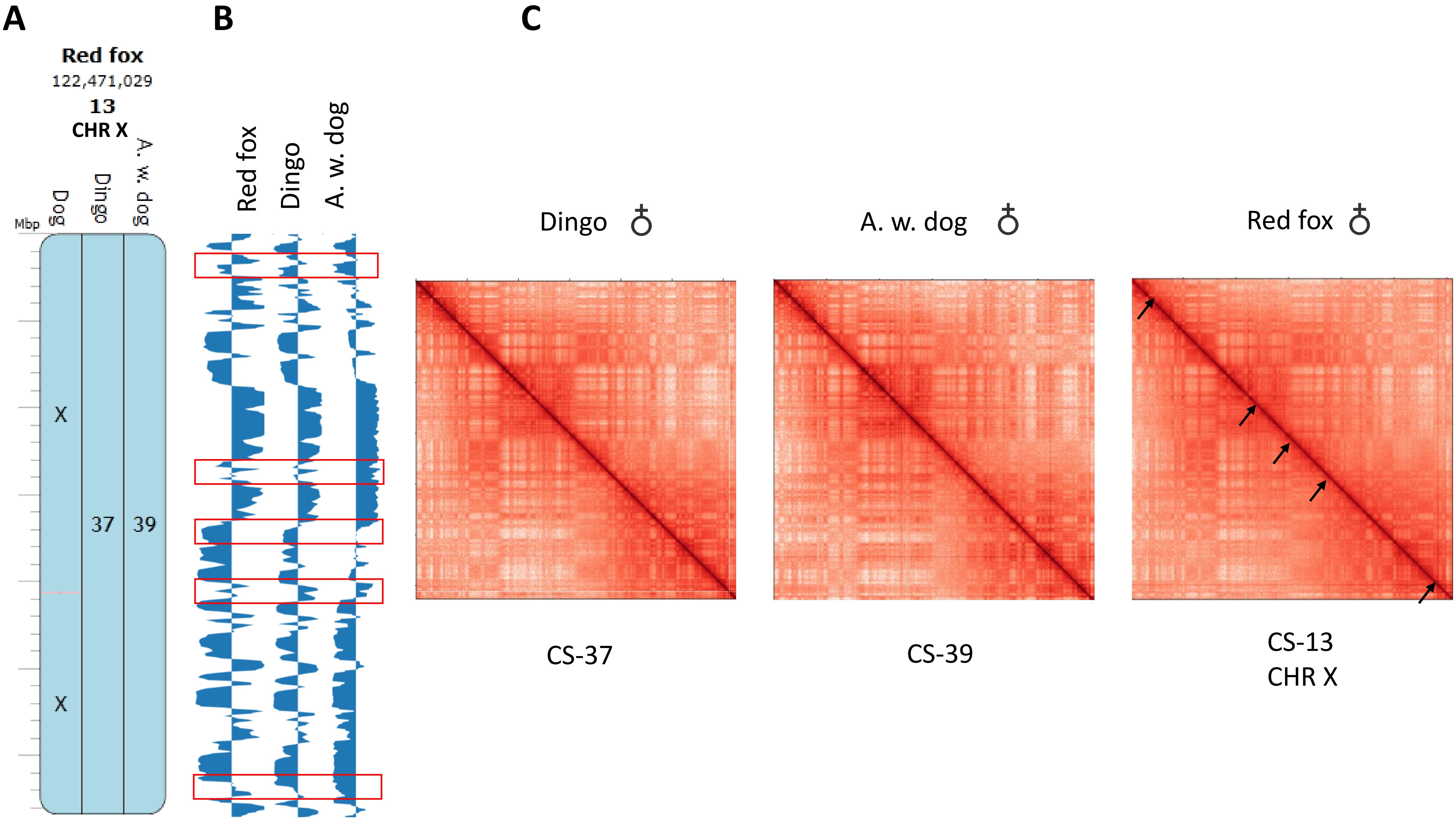
A
B
C
Red fox
122,471,029
13
CHR X
Mbp
Dog
Dingo
A. w. dog
X
X
37
39
Red fox
Dingo
A. w. dog
Dingo ♂
A. w. dog ♂
Red fox ♂
CS-37
CS-39
CS-13
CHR X

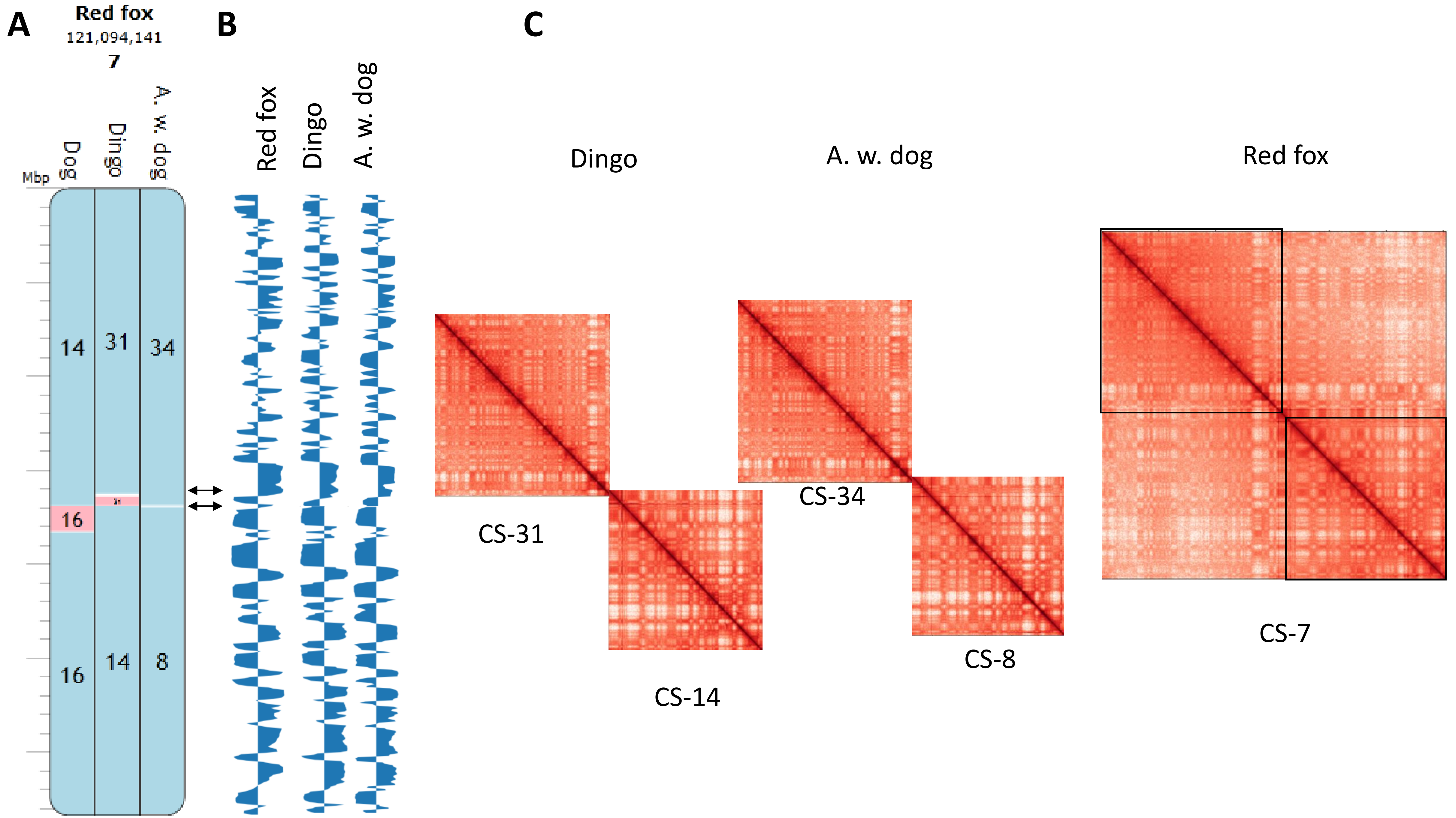
A
Red fox
121,094,141
7
Mbp
Dog
Dingo
A. w. dog
14
31
34
16
16
14
8
B
Red fox
Dingo
A. w. dog
C
Dingo
A. w. dog
Red fox
CS-31
CS-14
CS-34
CS-8
CS-7

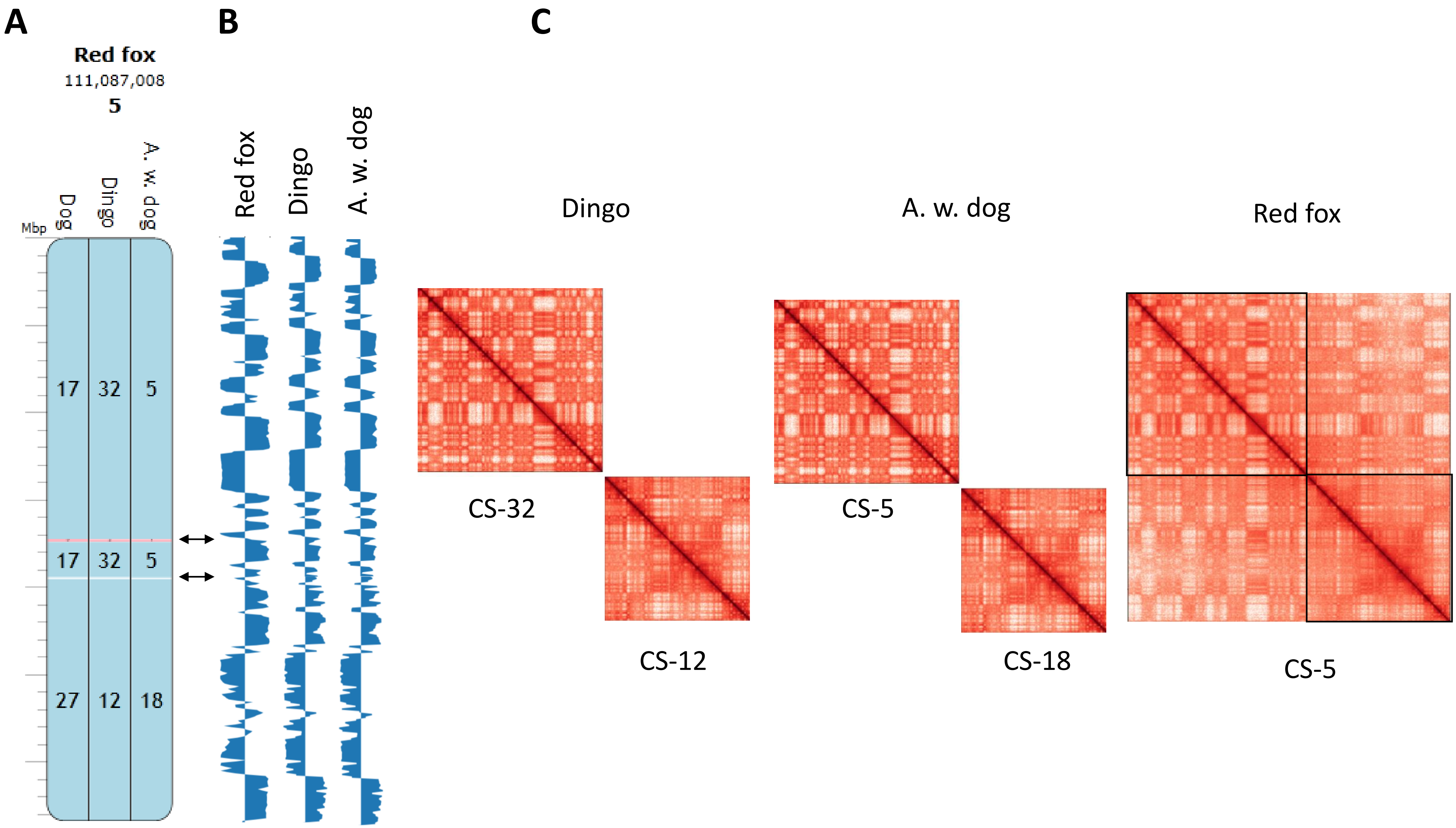
A
Red fox
111,087,008
5
Mbp
Dog
Dingo
A. w. dog
17
32
5
17
32
5
27
12
18
B
Red fox
Dingo
A. w. dog
C
Dingo
A. w. dog
Red fox
CS-32
CS-12
CS-5
CS-18
CS-5

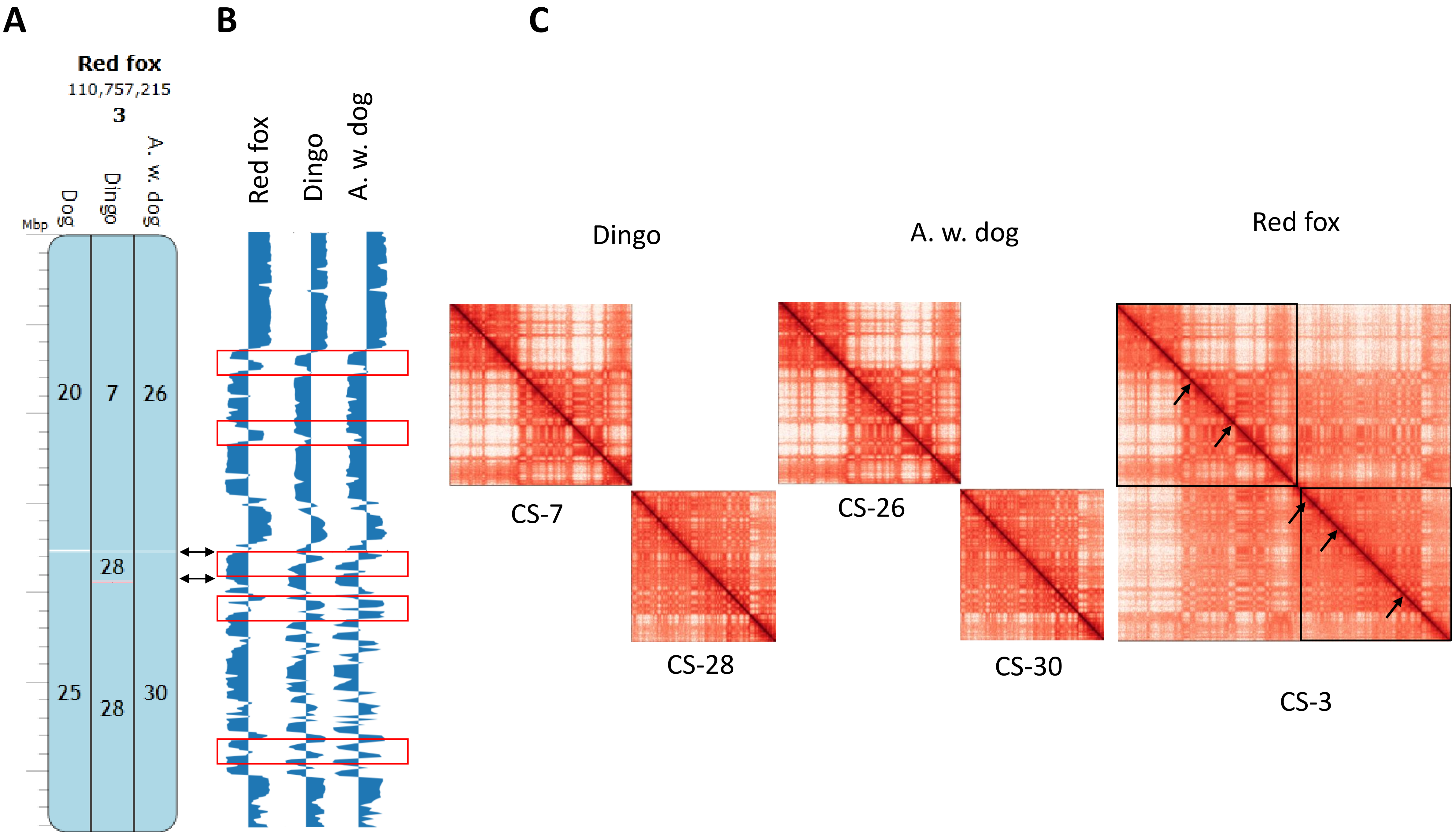
A
B
C
Red fox
110,757,215
3
Mbp
Dog
Dingo
A. w. dog
20
7
26
28
25
28
30
Red fox
Dingo
A. w. dog
Dingo
A. w. dog
Red fox
CS-7
CS-28
CS-26
CS-30
CS-3

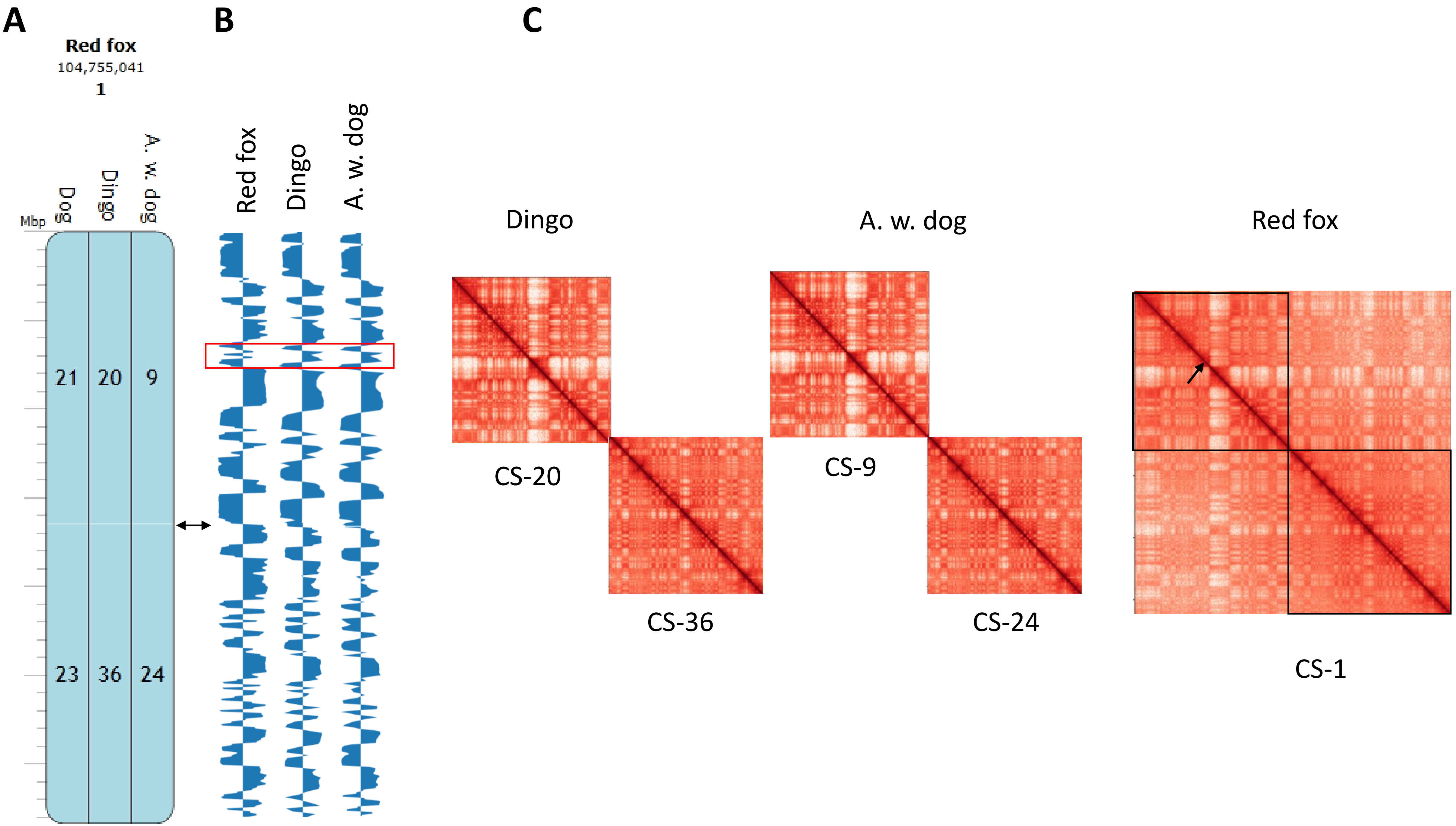

A
Red fox
104,755,041
1
Mbp
Dog
Dingo
A. w. dog
21
20
9
23
36
24
B
Red fox
Dingo
A. w. dog
C
Dingo
A. w. dog
Red fox
CS-20
CS-36
CS-9
CS-24
CS-1

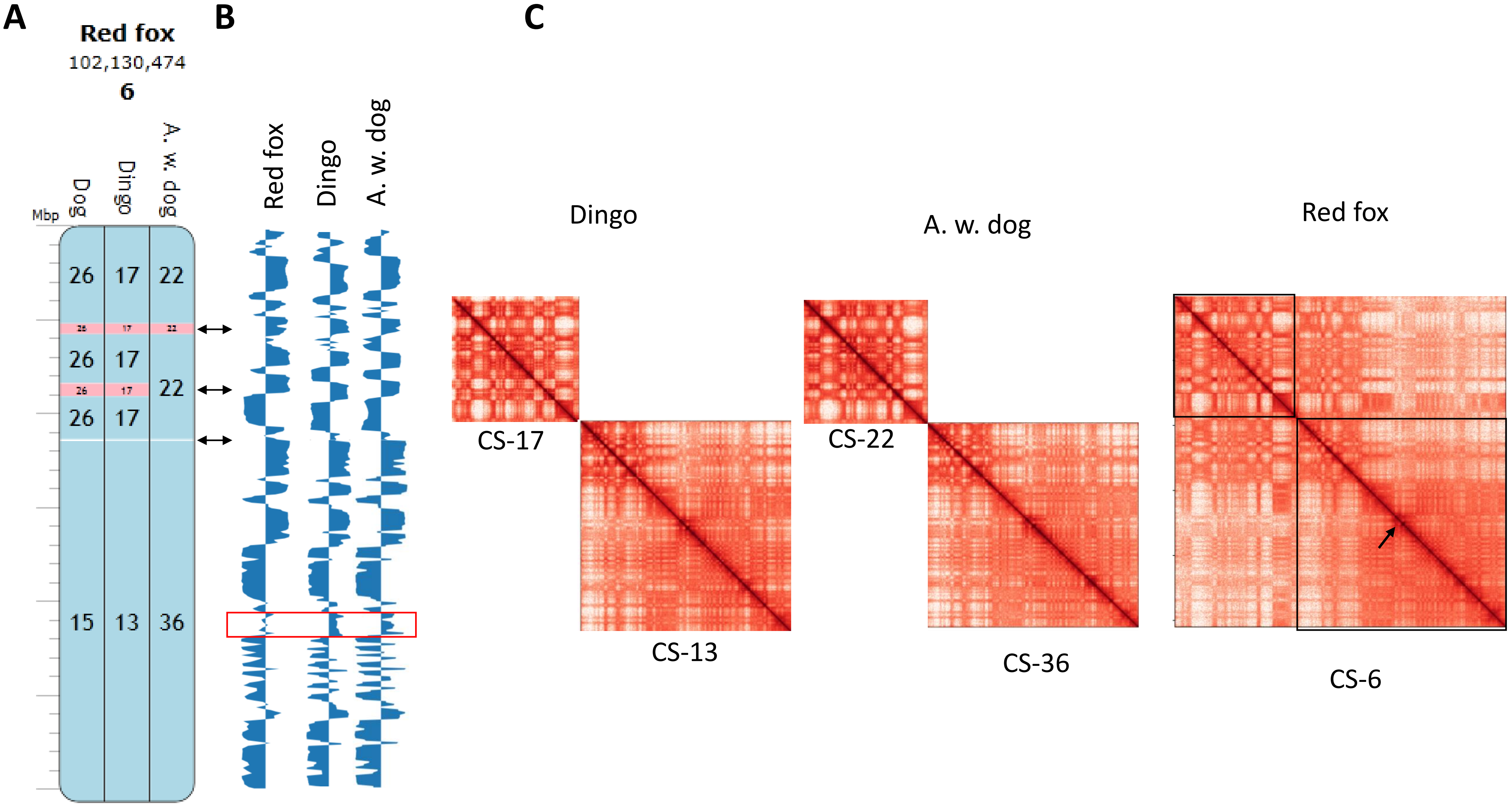

A
Red fox
102,130,474
6
Mbp
Dog
Dingo
A. w. dog
26
17
22
15
13
36
B
Red fox
Dingo
A. w. dog
C
Dingo
A. w. dog
Red fox
CS-17
CS-13
CS-22
CS-36
CS-6

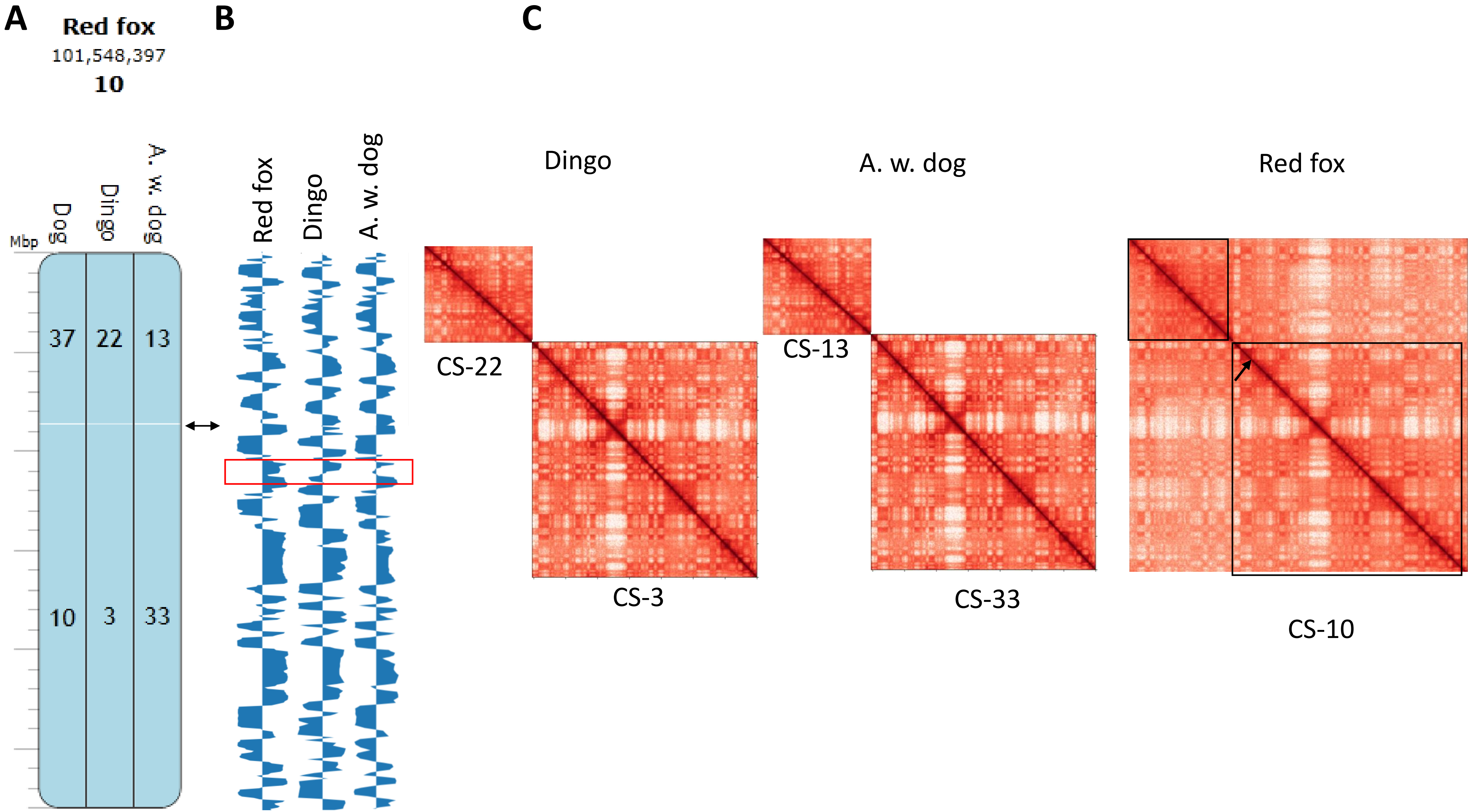
A
Red fox
101,548,397
10
Mbp
Dog
Dingo
A. w. dog
37
22
13
10
3
33
B
Red fox
Dingo
A. w. dog
C
Dingo
A. w. dog
Red fox
CS-22
CS-3
CS-13
CS-33
CS-10

Figure S3. Comparative chromatin conformation analysis in canids.

Orthologous relationships between dog chromosomes, dingo and African wild dog C-scaffolds and red fox C-scaffolds. (A) Homologous synteny blocks of the three canid species visualized in Evolution Highway at 300 kb resolution. Blue indicates same sequence orientation as the reference genome. Pink depicts chromosome inversions. Numbers represent the scaffold identifier of the target species. (B) Eigenvector values of each species aligned to the red fox reference genome at 500 kb resolution. Red boxes highlight compartment shift. (C) Juicer plots of C-scaffolds for dingo, African wild dog and red fox. Color intensity reflects the frequency of interactions between pairs of loci on the C-scaffolds (range 1-1,000 for each map). Boxes with different colors within the Hi-C plots highlight subchromosomal fragments of the dingo and wild dog C-scaffolds that are orthologous to subchromosomal fragments of red fox C-scaffolds. Arrows indicate position of compartment change. Alignment coordinates can be found in SI Dataset S5 We were unable to investigate the chromatin structure of red fox B chromosomes mostly because their intrinsic repetitive sequence content and the inability to map the putative B chromosome contigs to the assembly (7).

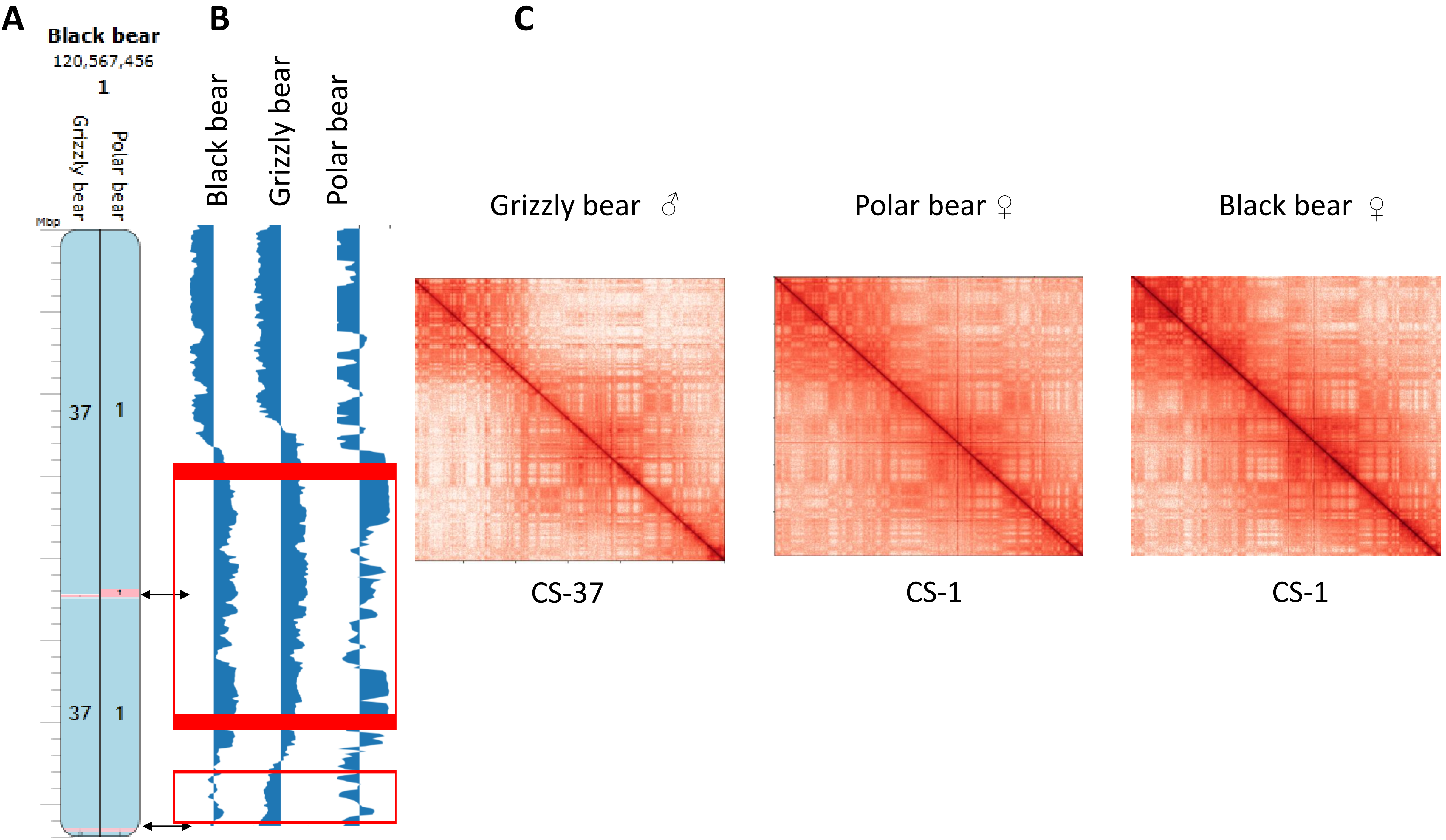

A
Black bear
120,567,456
1
Grizzly bear
Polar bear
Mbp
37
1
37
1
B
Black bear
Grizzly bear
Polar bear
C
Grizzly bear ♂
Polar bear ♀
Black bear ♀
CS-37
CS-1
CS-1

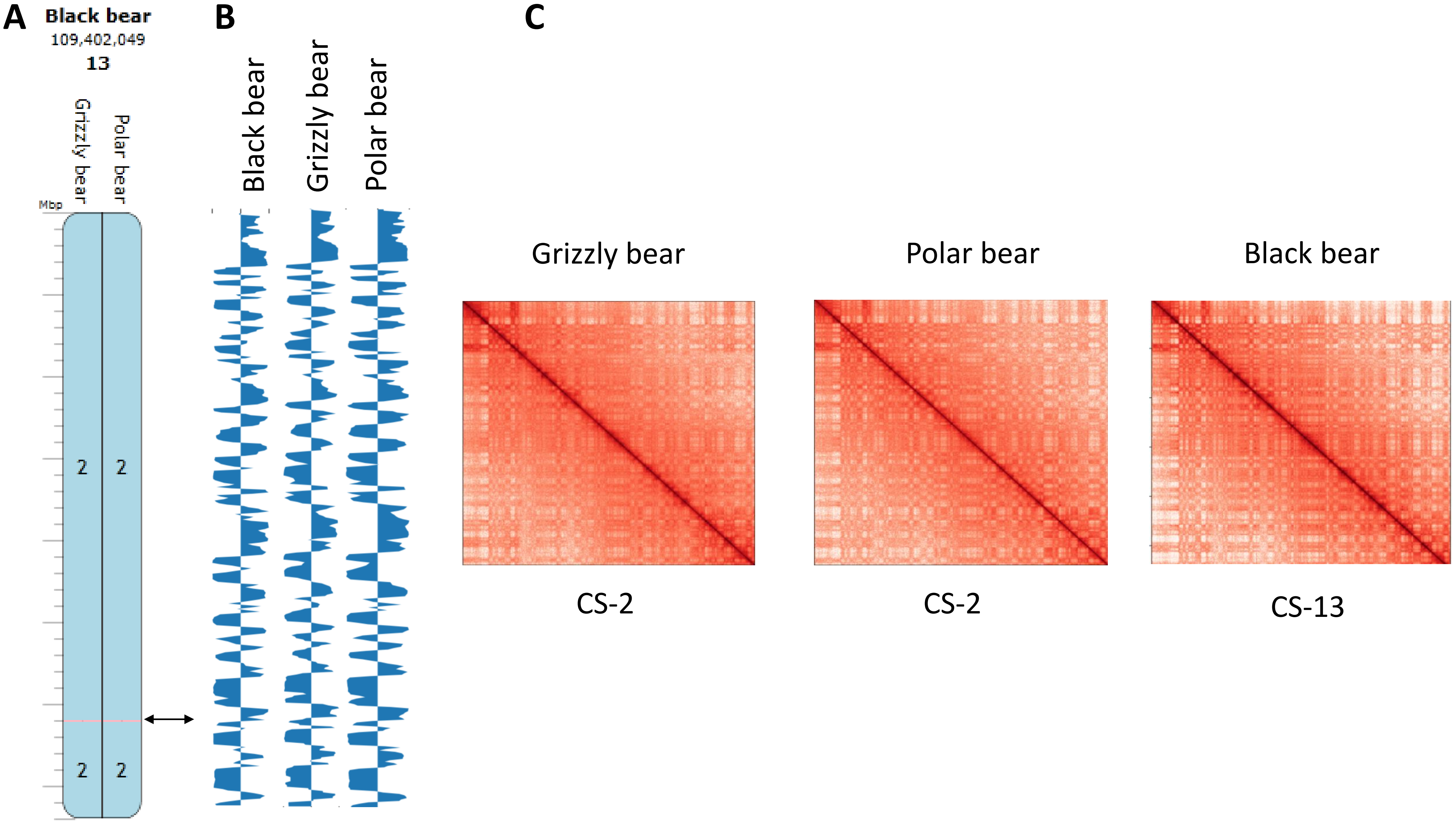
A
Black bear
109,402,049
13
Grizzly bear
Polar bear
Mbp
2
2
2
2
B
Black bear
Grizzly bear
Polar bear
C
Grizzly bear
Polar bear
Black bear
CS-2
CS-2
CS-13

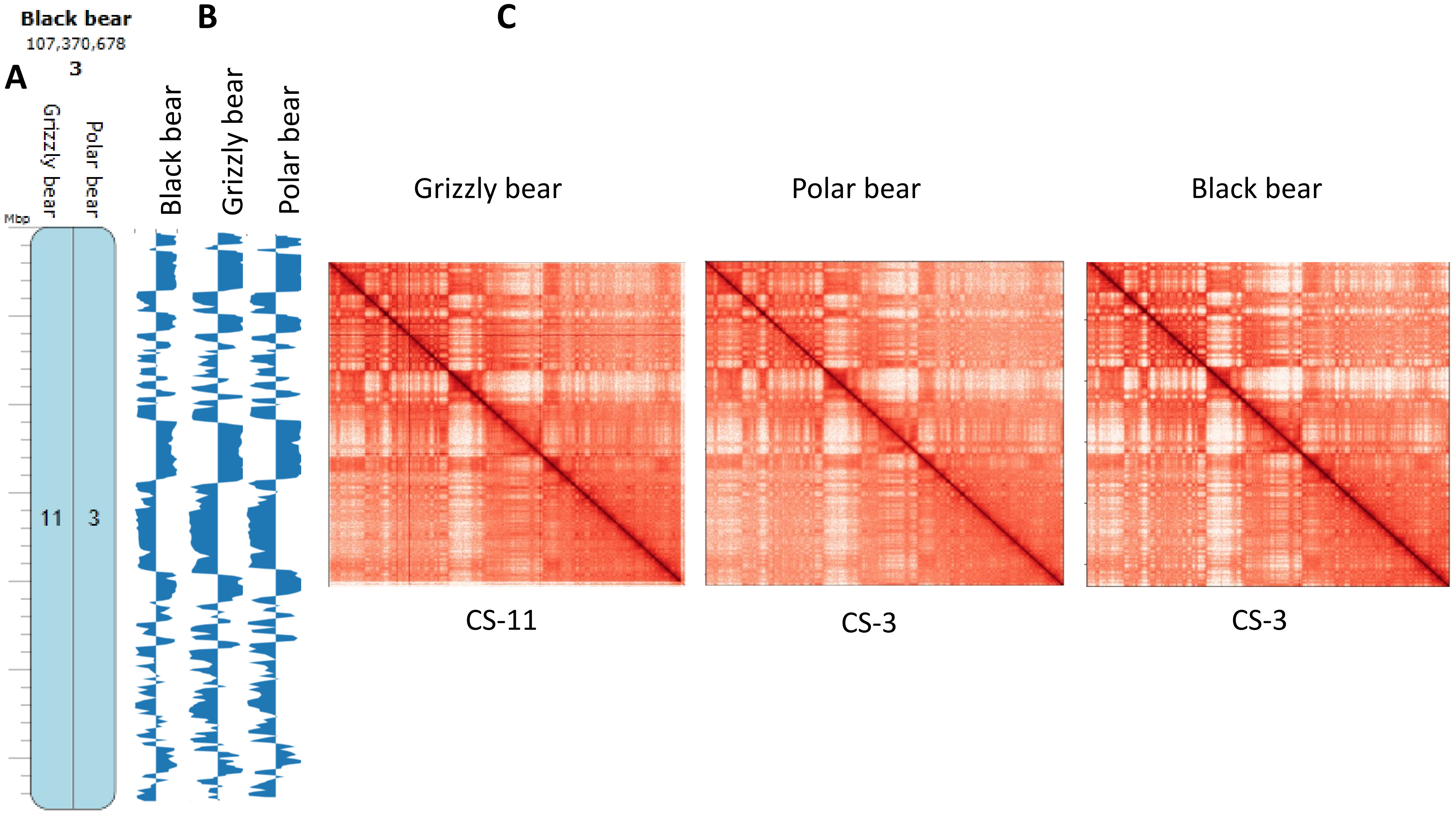
Black bear
107,370,678
3
A
B
C
Grizzly bear
Polar bear
Mbp
11
3
Black bear
Grizzly bear
Polar bear
Grizzly bear
Polar bear
Black bear
CS-11
CS-3
CS-3

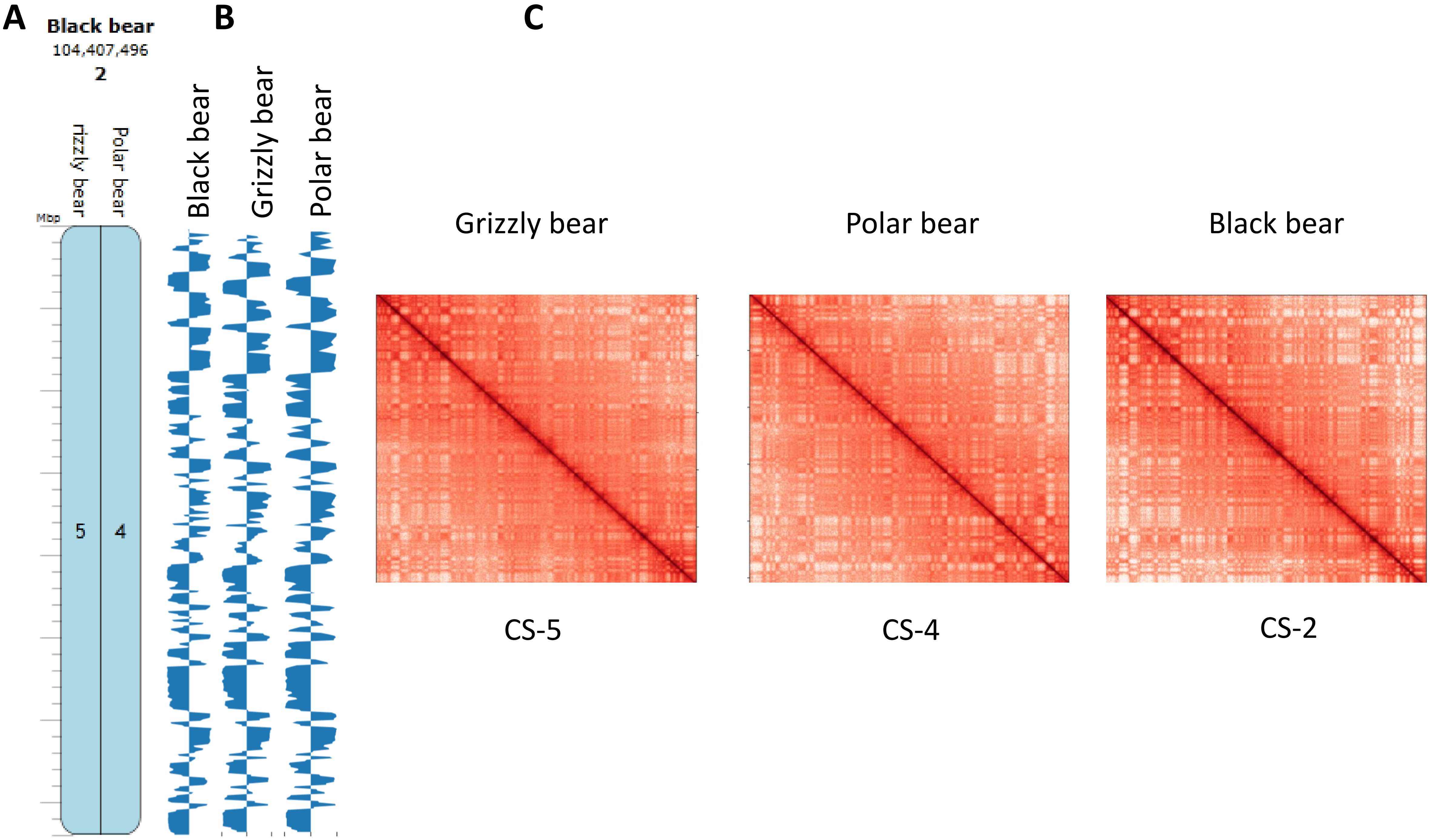
A
Black bear
104,407,496
2
rizzly bear
Polar bear
Mbp
5
4
B
Black bear
Grizzly bear
Polar bear
C
Grizzly bear
Polar bear
Black bear
CS-5
CS-4
CS-2

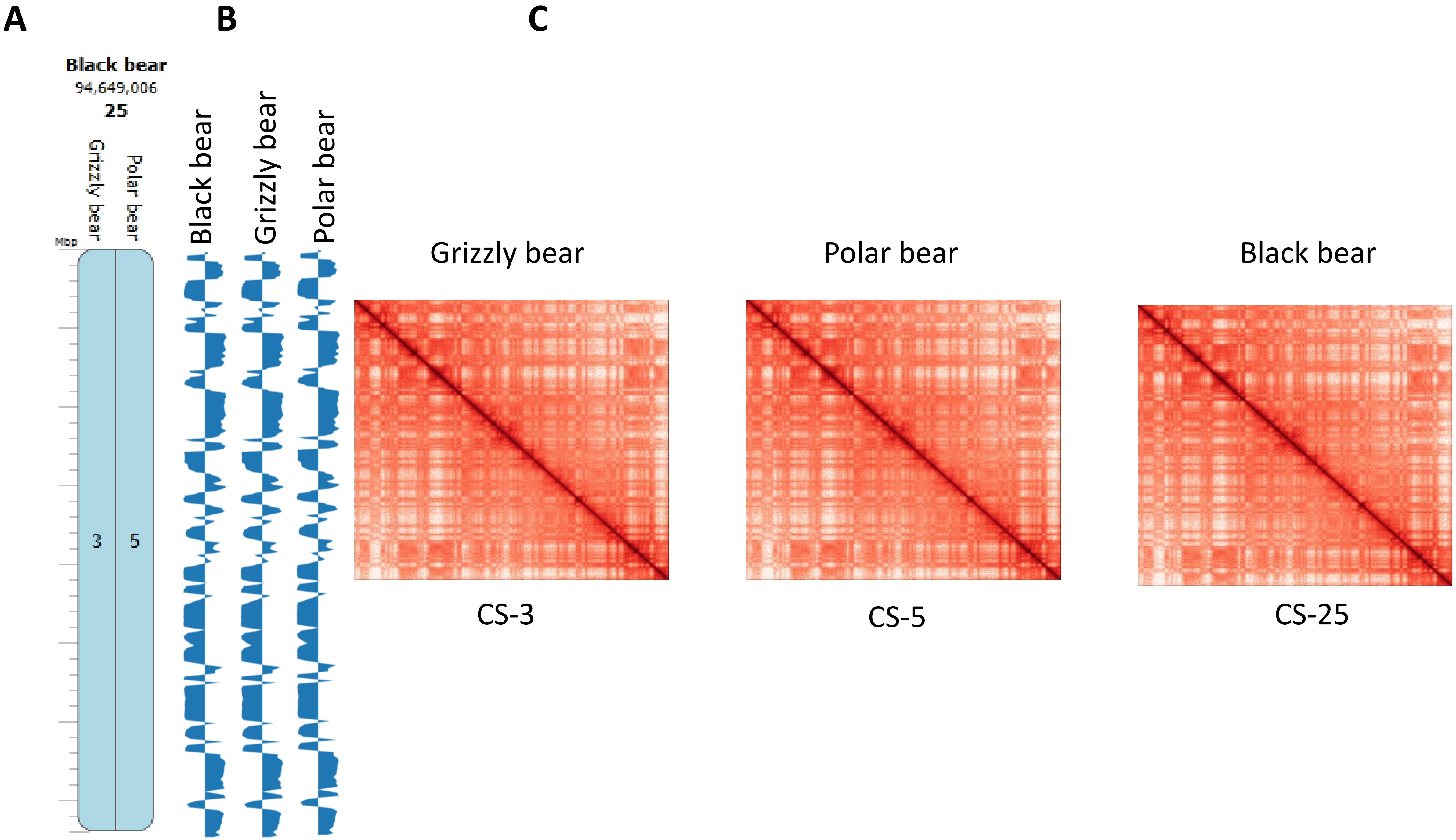
A
B
C
Black bear
94,649,006
25
Grizzly bear
Polar bear
Mbp
3
5
Black bear
Grizzly bear
Polar bear
Grizzly bear
Polar bear
Black bear
CS-3
CS-5
CS-25

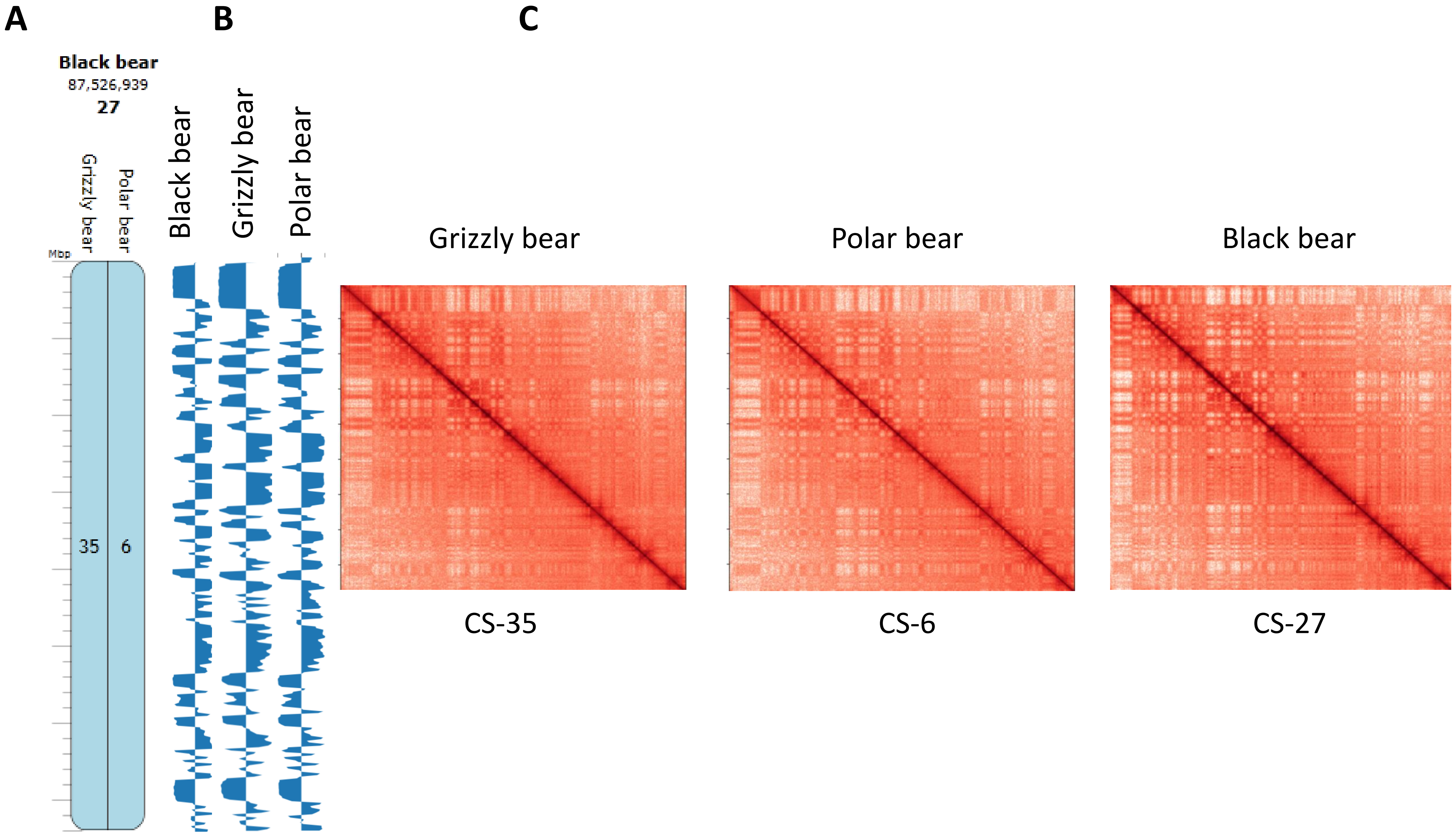
A
B
C
Black bear
87,526,939
27
Grizzly bear
Polar bear
Mbp
35
6
Black bear
Grizzly bear
Polar bear
Grizzly bear
Polar bear
Black bear
CS-35
CS-6
CS-27

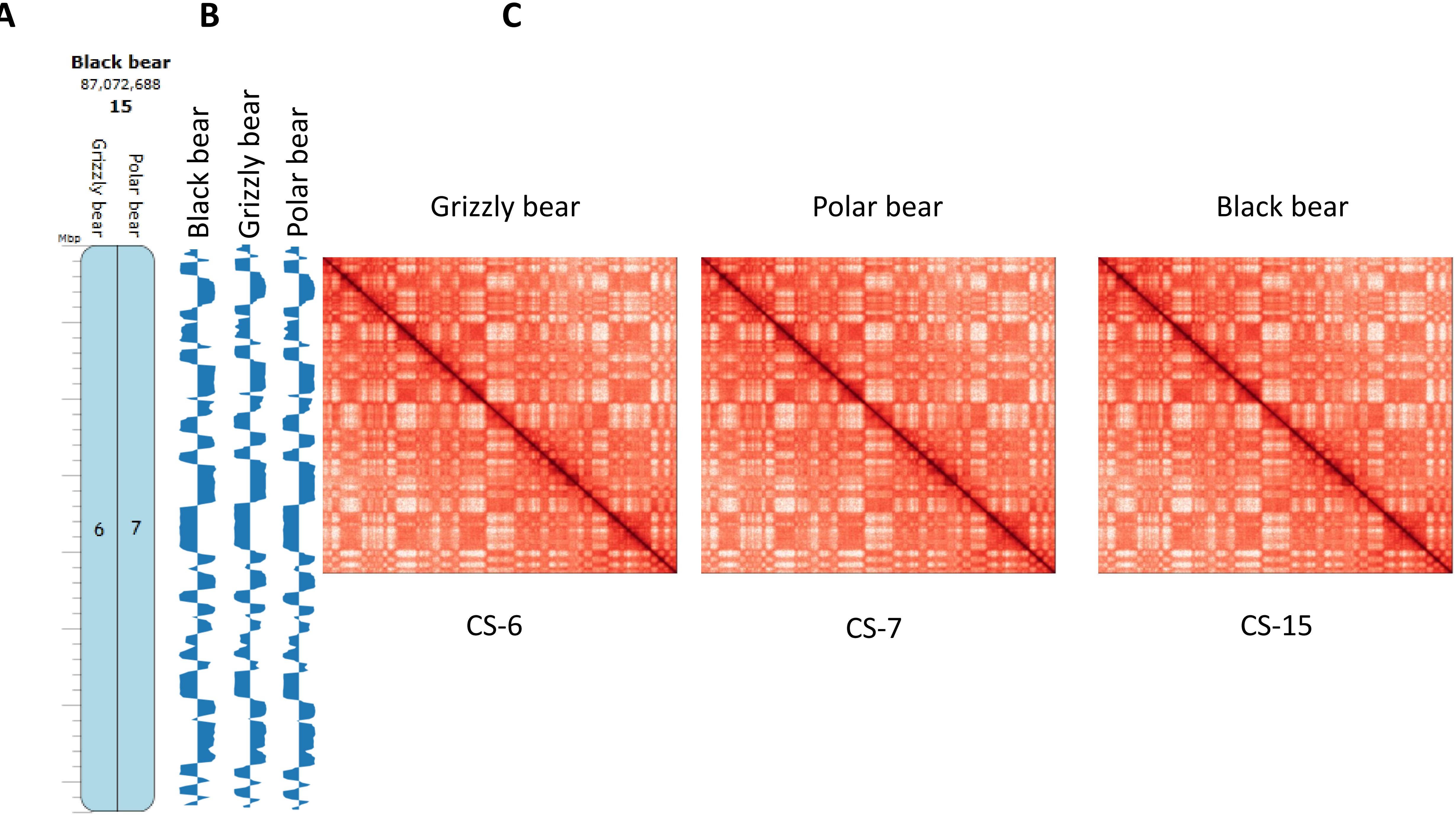
A
B
C
Black bear
87,072,688
15
Grizzly bear
Polar bear
Mbp
6
7
Black bear
Grizzly bear
Polar bear
Grizzly bear
Polar bear
Black bear
CS-6
CS-7
CS-15

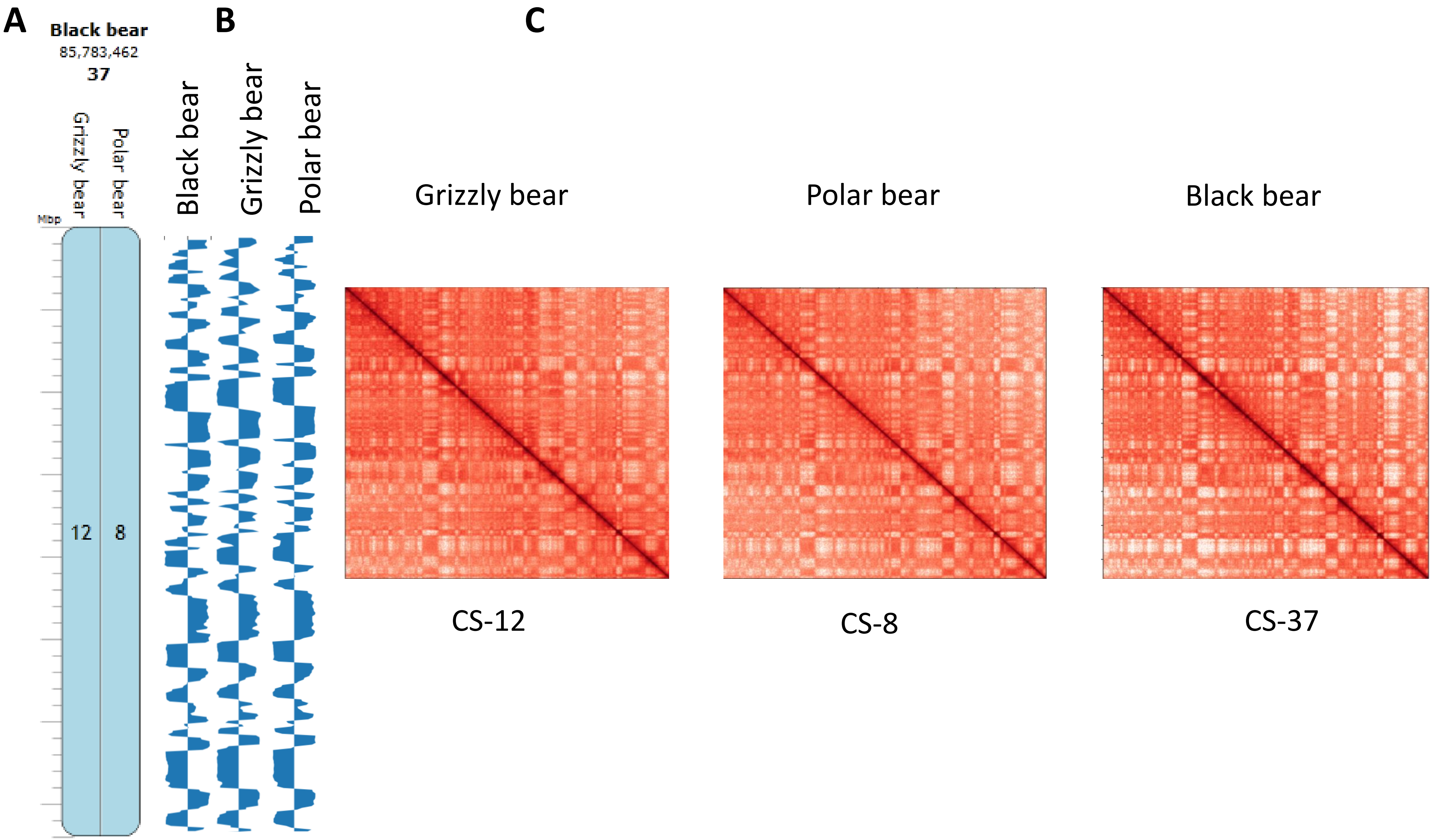
A
Black bear
85,783,462
37
Grizzly bear
Polar bear
Mbp
12
8
B
Black bear
Grizzly bear
Polar bear
C
Grizzly bear
Polar bear
Black bear
CS-12
CS-8
CS-37

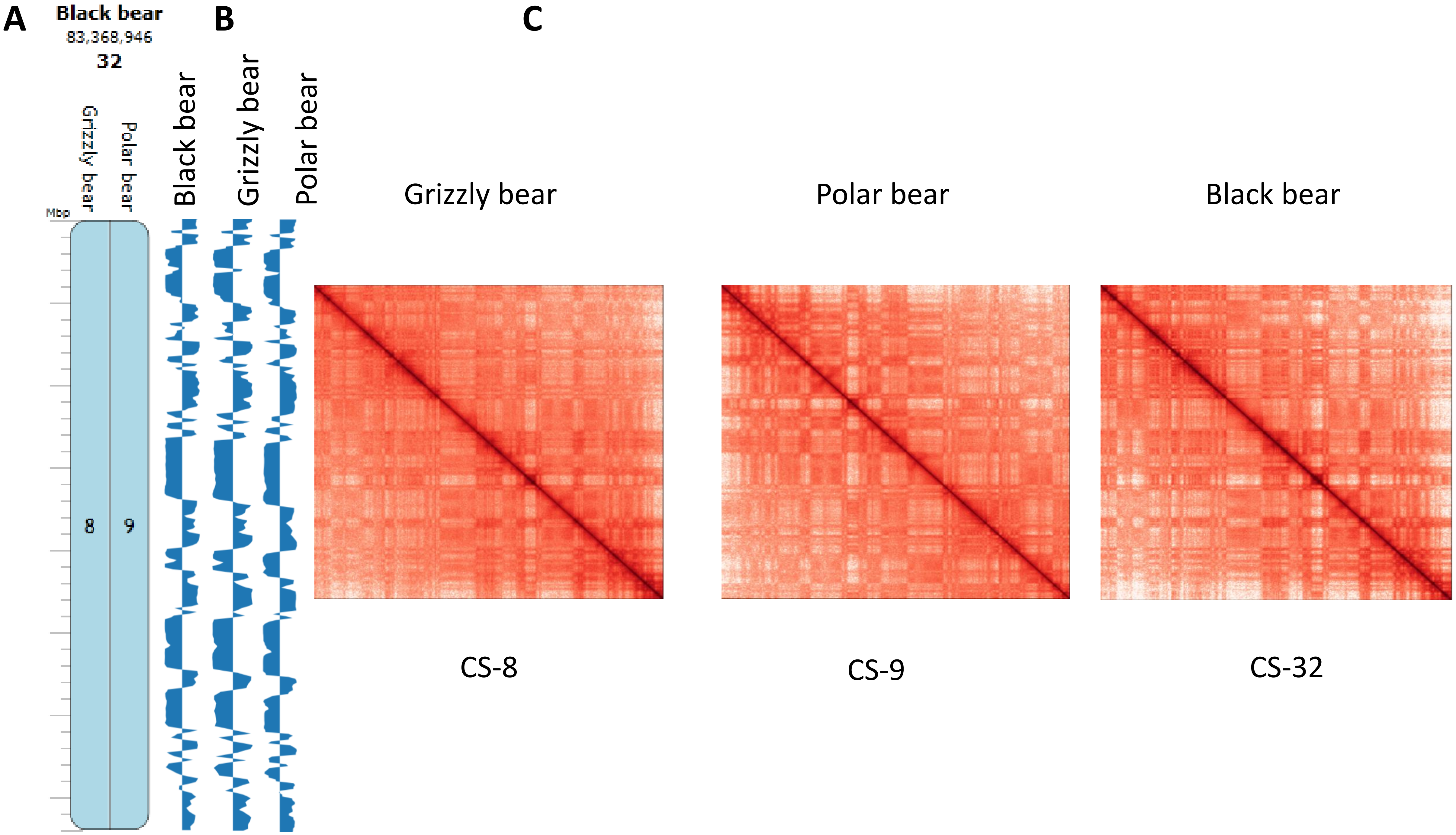
A
Black bear
83,368,946
32
Grizzly bear
Polar bear
Mbp
8
9
B
Black bear
Grizzly bear
Polar bear
C
Grizzly bear
Polar bear
Black bear
CS-8
CS-9
CS-32

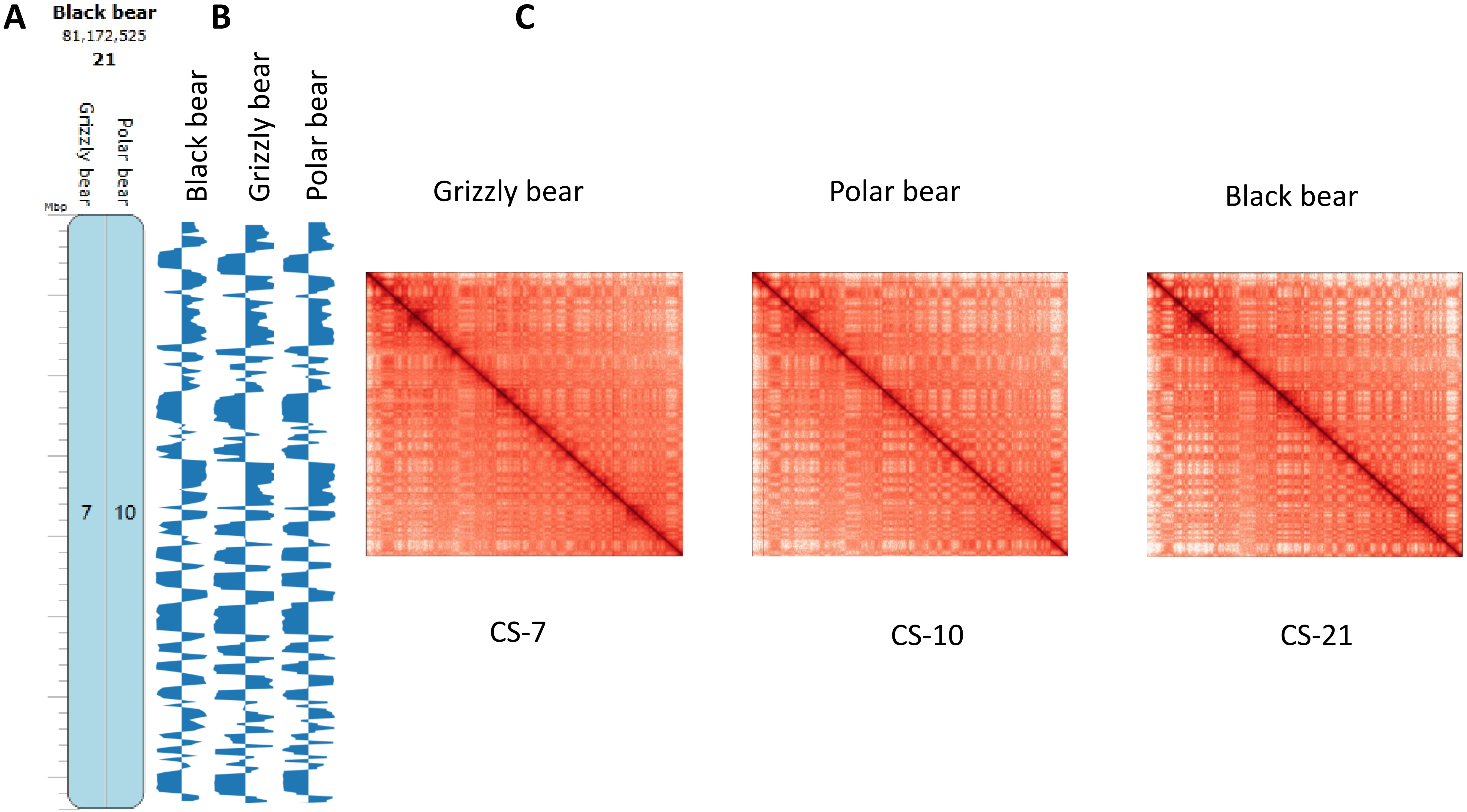
A
Black bear
81,172,525
21
Grizzly bear
Polar bear
Mbp
7
10
B
Black bear
Grizzly bear
Polar bear
C
Grizzly bear
Polar bear
Black bear
CS-7
CS-10
CS-21

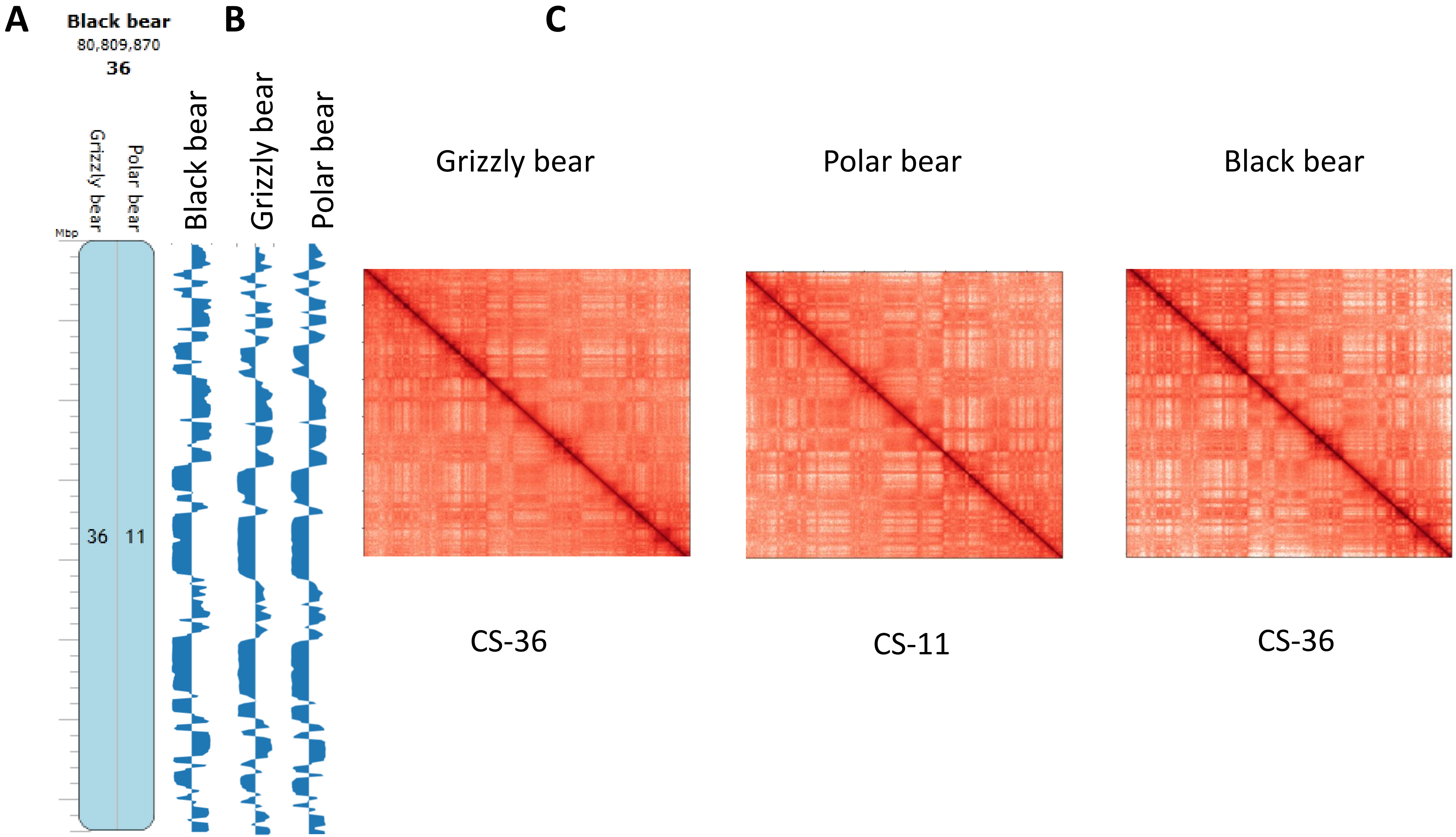
A
Black bear
80,809,870
36
Grizzly bear
Polar bear
Mbp
36
11
B
Black bear
Grizzly bear
Polar bear
C
Grizzly bear
Polar bear
Black bear
CS-36
CS-11
CS-36

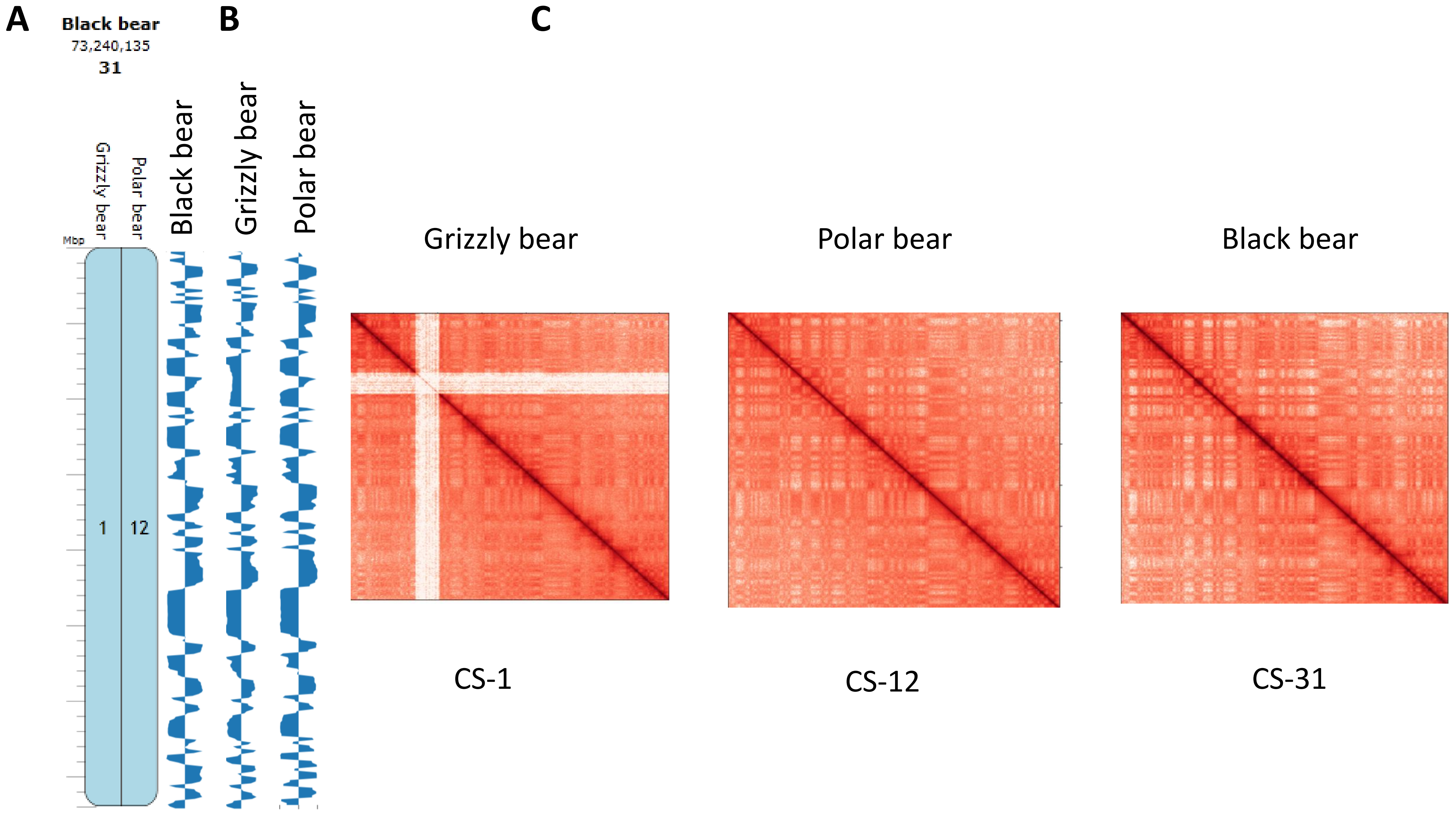
A
Black bear
73,240,135
31
Grizzly bear
Polar bear
Mbp
1
12
B
Black bear
Grizzly bear
Polar bear
C
Grizzly bear
Polar bear
Black bear
CS-1
CS-12
CS-31

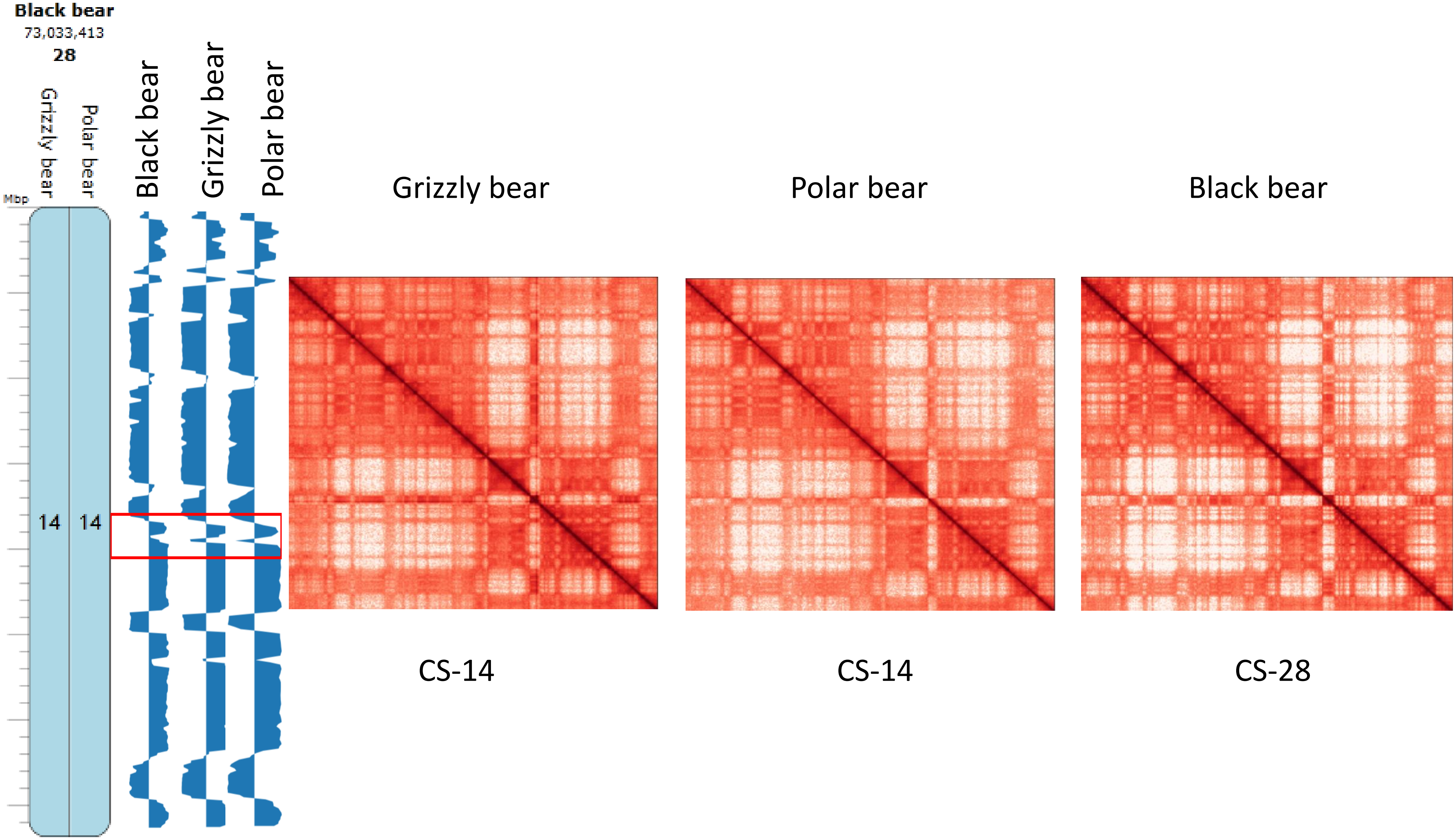

Black bear
73,033,413
28
Grizzly bear
Polar bear
Mbp
14
14
Black bear
Grizzly bear
Polar bear
Grizzly bear
Polar bear
Black bear
CS-14
CS-14
CS-28

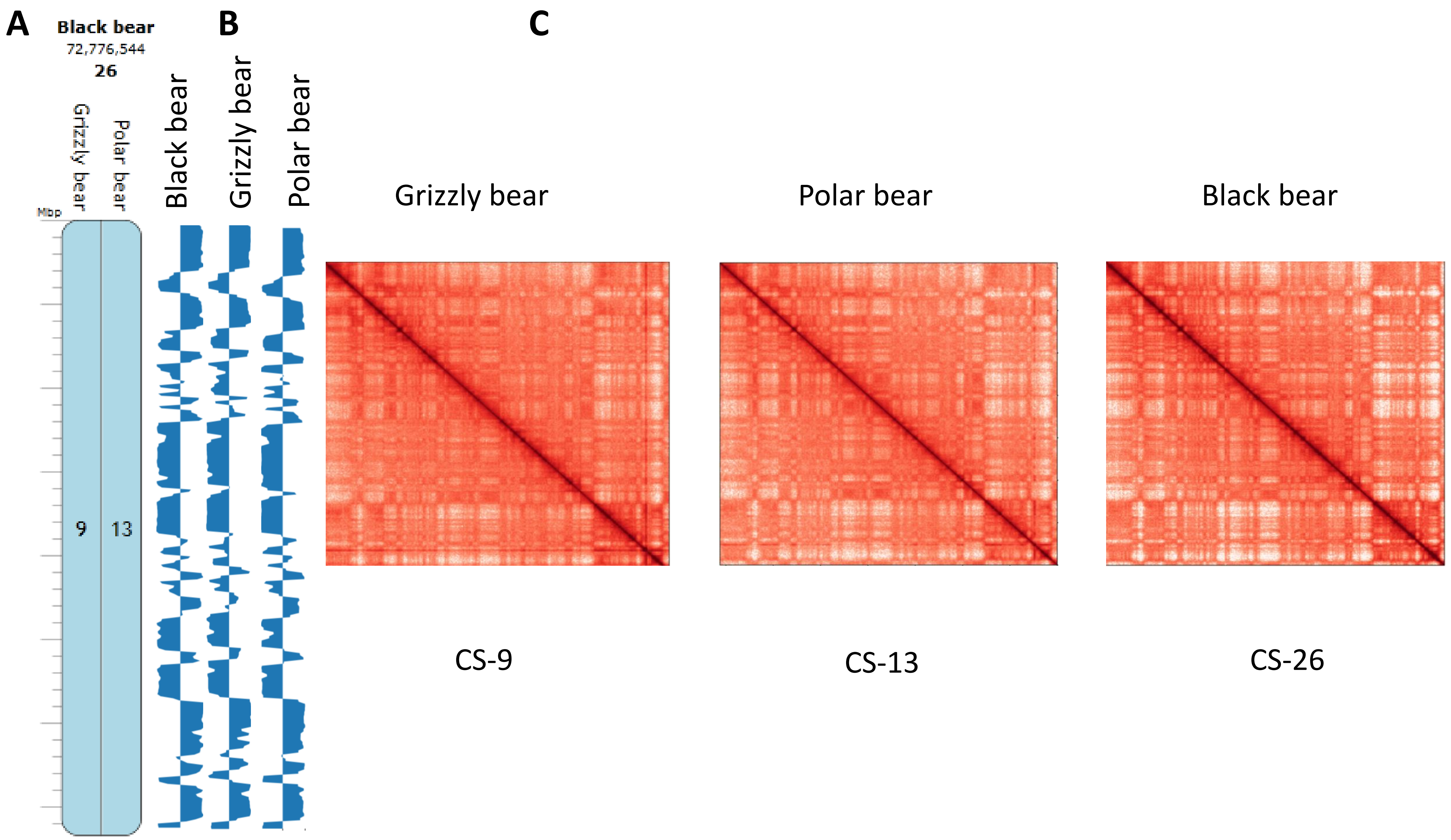
A
Black bear
72,776,544
26
Grizzly bear
Polar bear
Mbp
9
13
B
Black bear
Grizzly bear
Polar bear
C
Grizzly bear
Polar bear
Black bear
CS-9
CS-13
CS-26

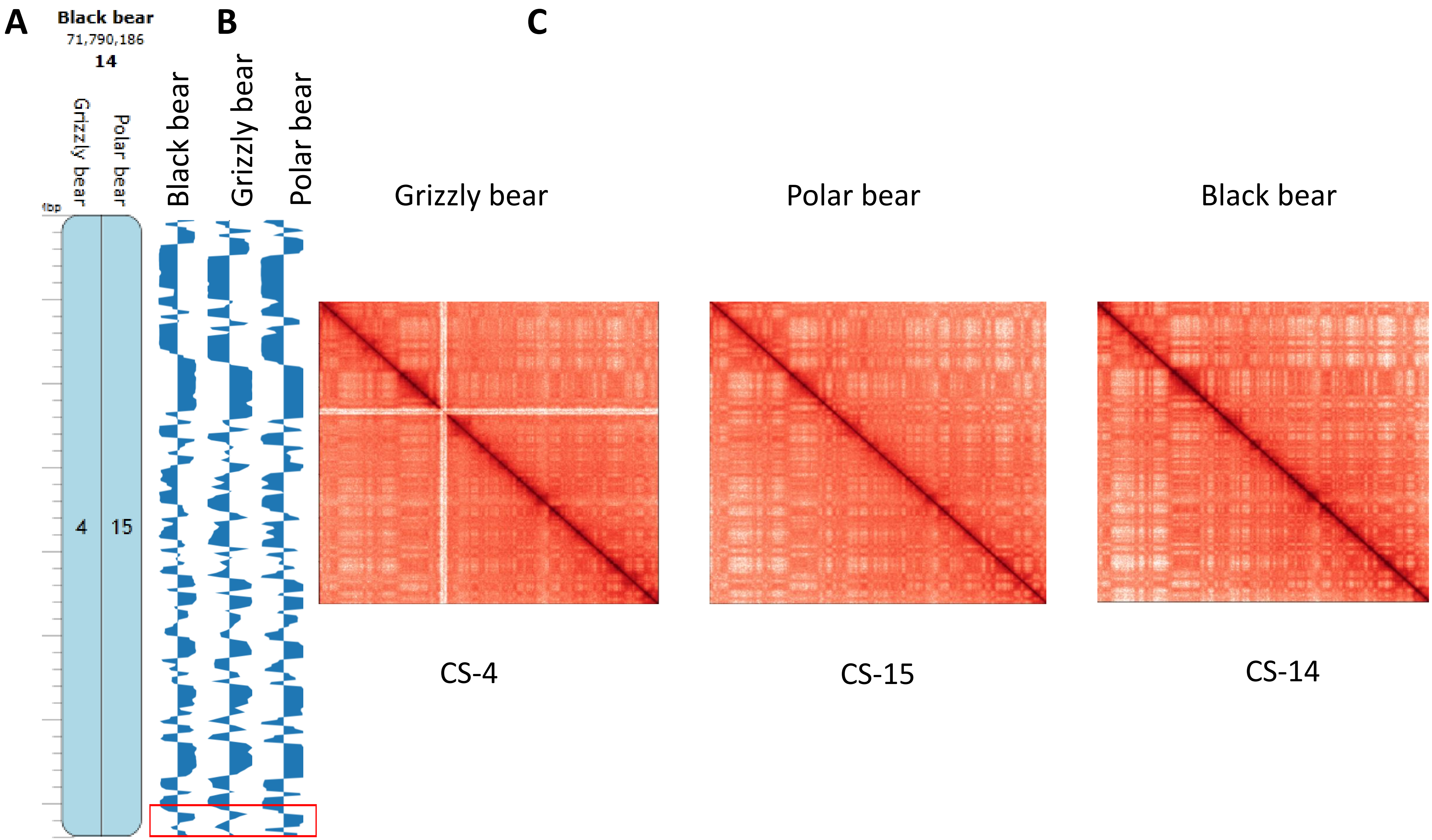
A
Black bear
71,790,186
14
Grizzly bear
Polar bear
4
15
B
Black bear
Grizzly bear
Polar bear
C
Grizzly bear
Polar bear
Black bear
CS-4
CS-15
CS-14

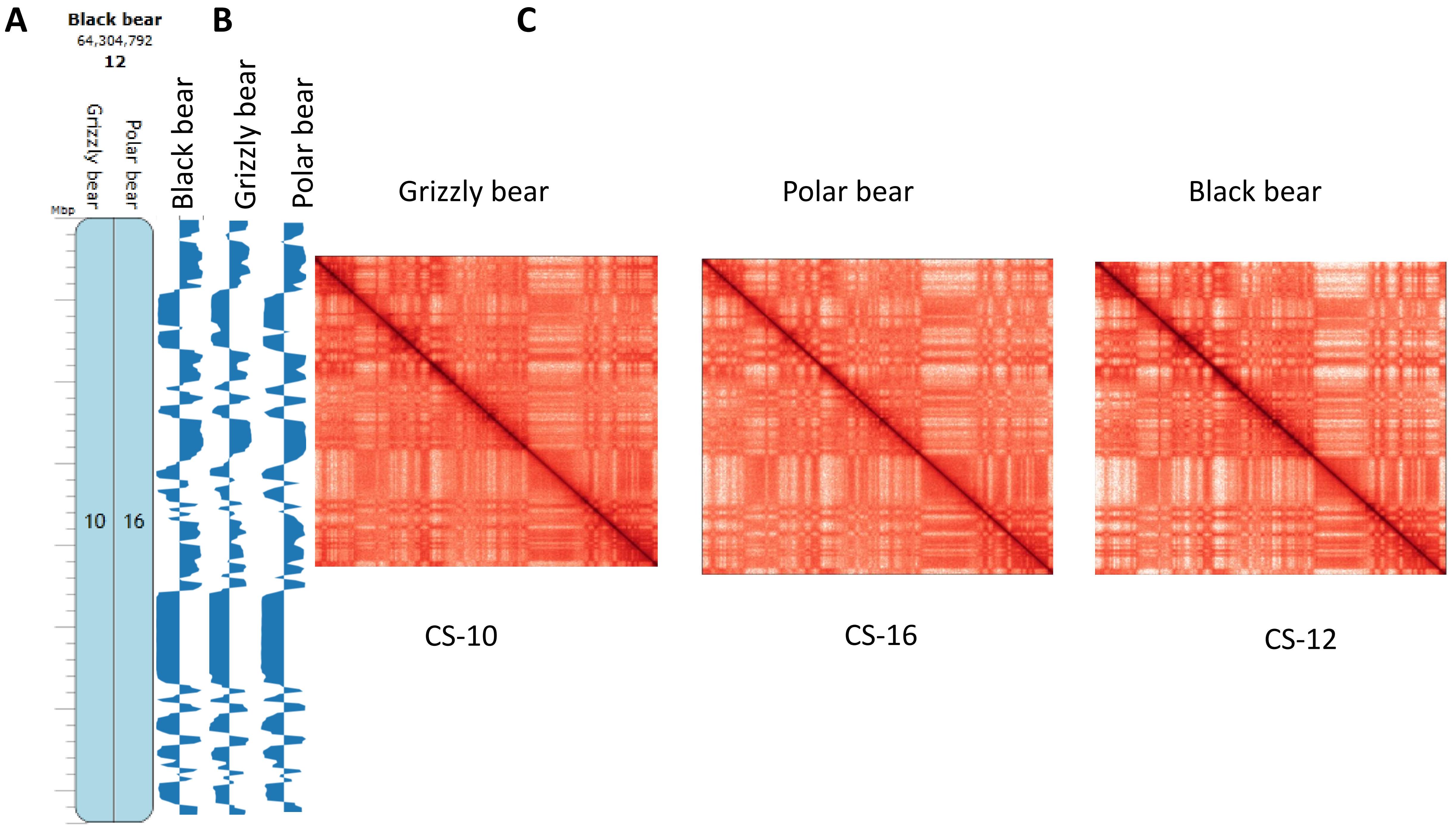
A
Black bear
64,304,792
12
Grizzly bear
Polar bear
Mbp
10
16
B
Black bear
Grizzly bear
Polar bear
C
Grizzly bear
Polar bear
Black bear
CS-10
CS-16
CS-12

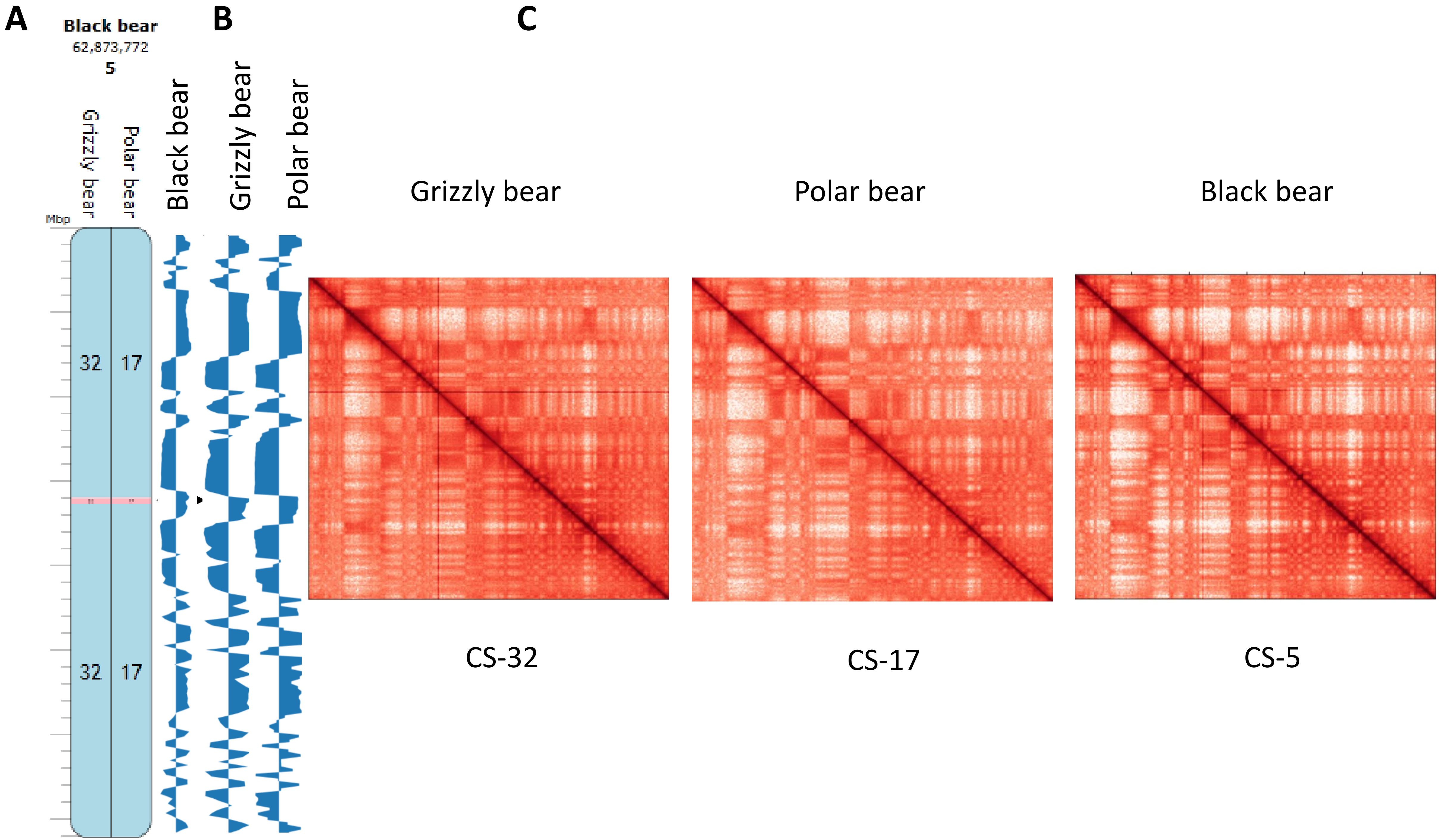
A
Black bear
62,873,772
5
Grizzly bear
Polar bear
Mbp
32
17
32
17
B
Black bear
Grizzly bear
Polar bear
C
Grizzly bear
Polar bear
Black bear
CS-32
CS-17
CS-5

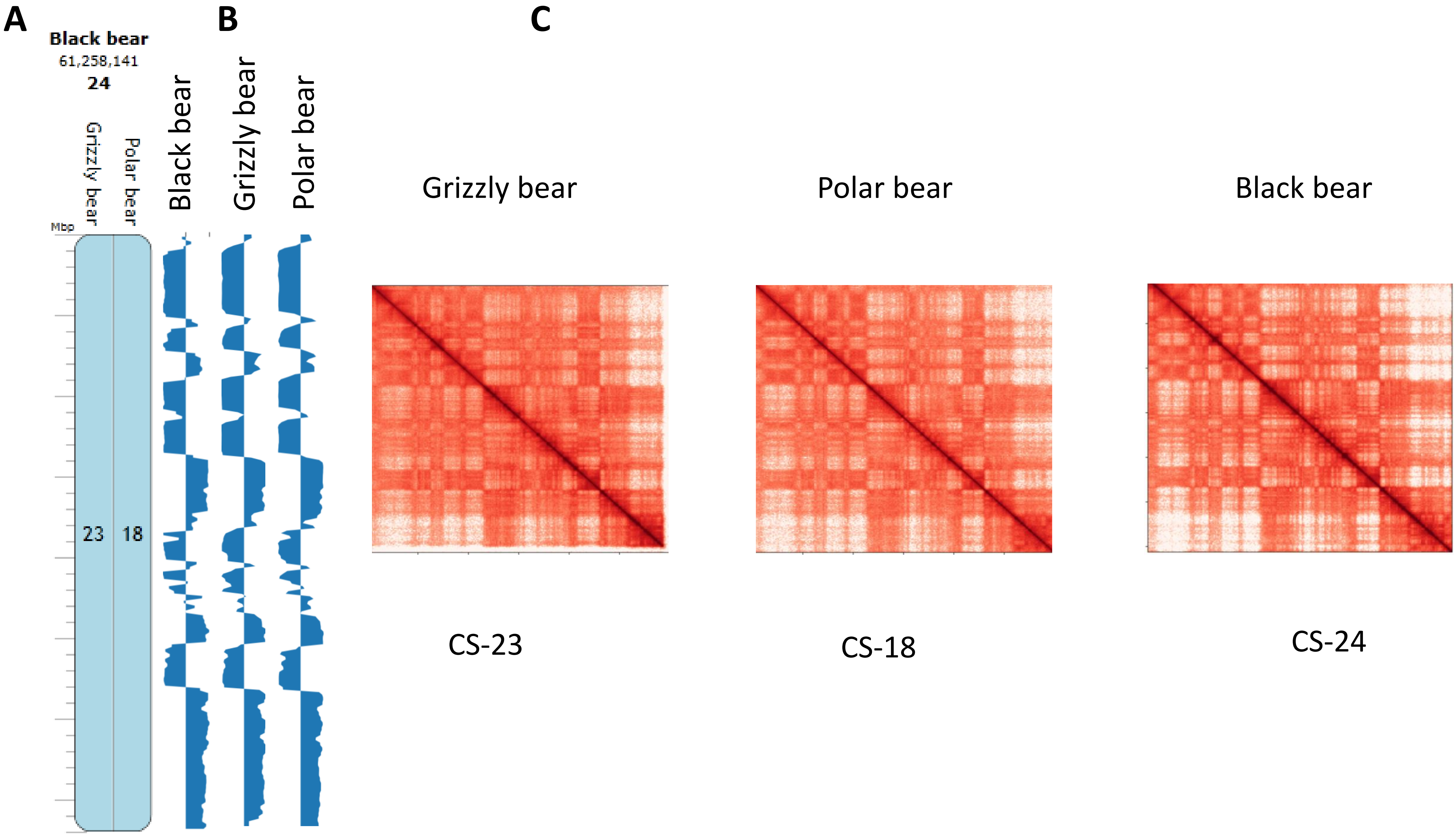
A
Black bear
61,258,141
24
Grizzly bear
Polar bear
Mbp
23
18
B
Black bear
Grizzly bear
Polar bear
C
Grizzly bear
Polar bear
Black bear
CS-23
CS-18
CS-24

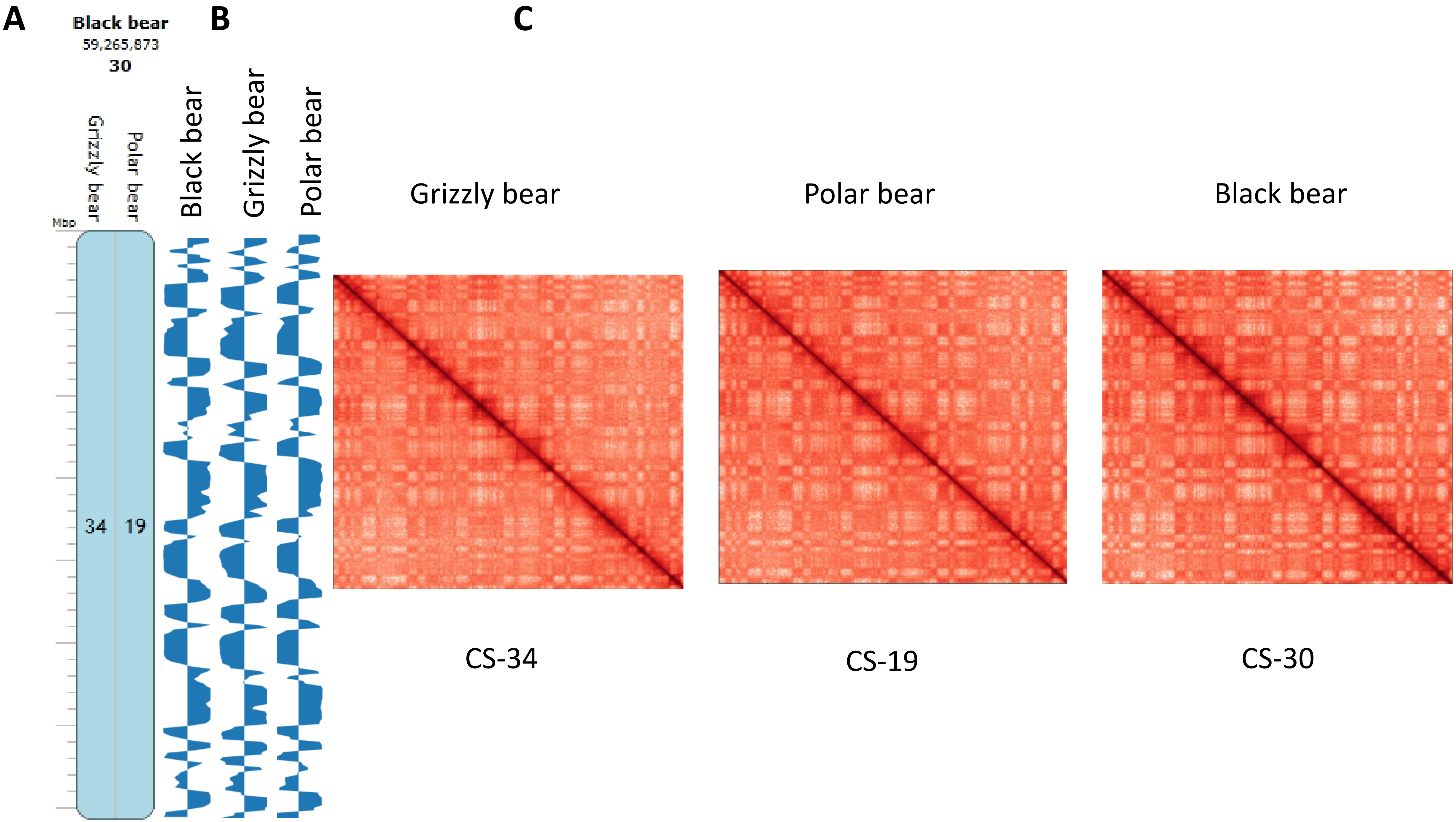
A
Black bear
59,265,873
30
Grizzly bear
Polar bear
Mbp
34
19
B
Black bear
Grizzly bear
Polar bear
C
Grizzly bear
Polar bear
Black bear
CS-34
CS-19
CS-30

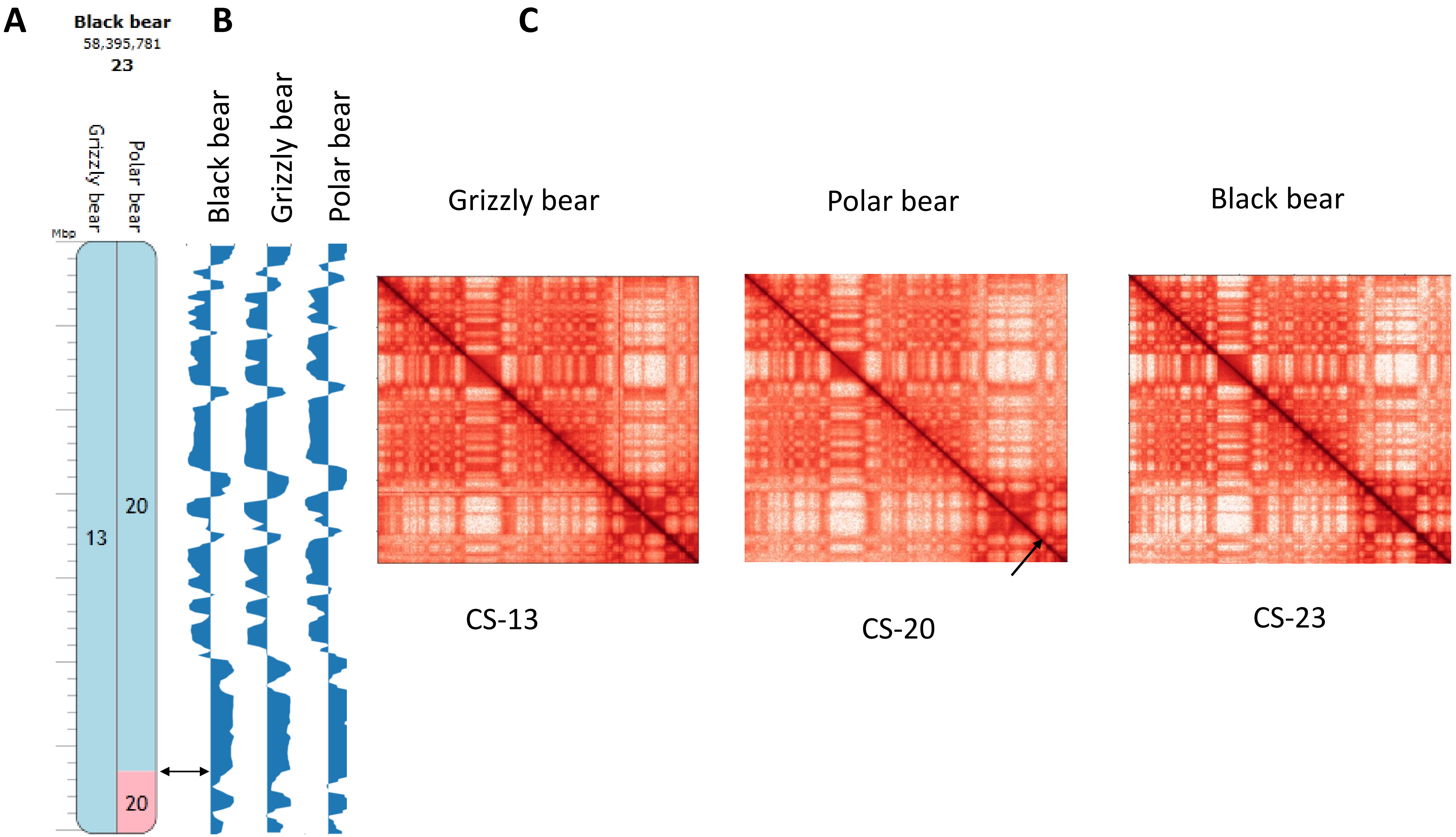
A
Black bear
58,395,781
23
Grizzly bear
Polar bear
Mbp
13
20
20
B
Black bear
Grizzly bear
Polar bear
C
Grizzly bear
Polar bear
Black bear
CS-13
CS-20
CS-23

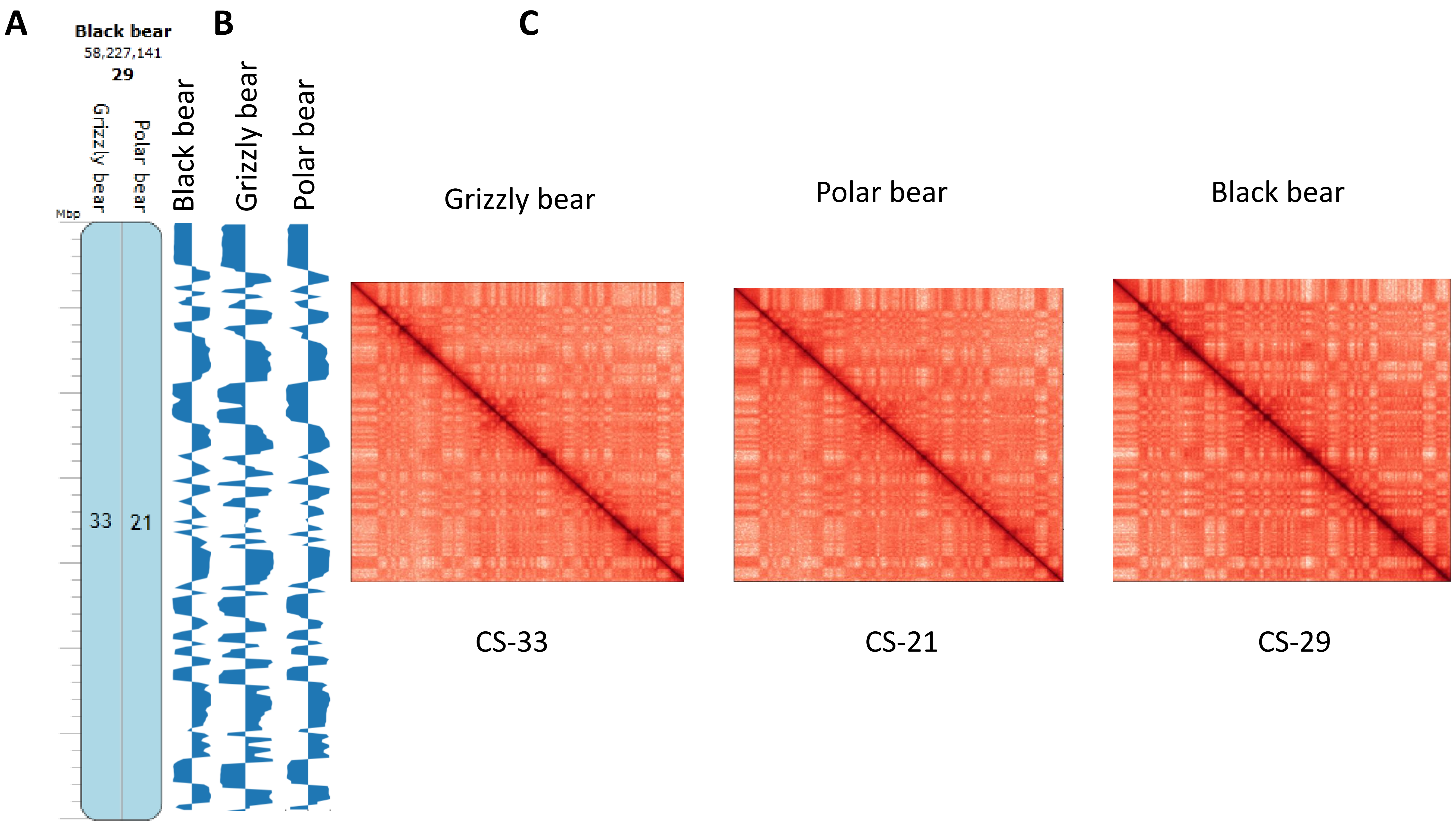
A
Black bear
58,227,141
29
Grizzly bear
Polar bear
Mbp
33
21
B
Black bear
Grizzly bear
Polar bear
C
Grizzly bear
Polar bear
Black bear
CS-33
CS-21
CS-29

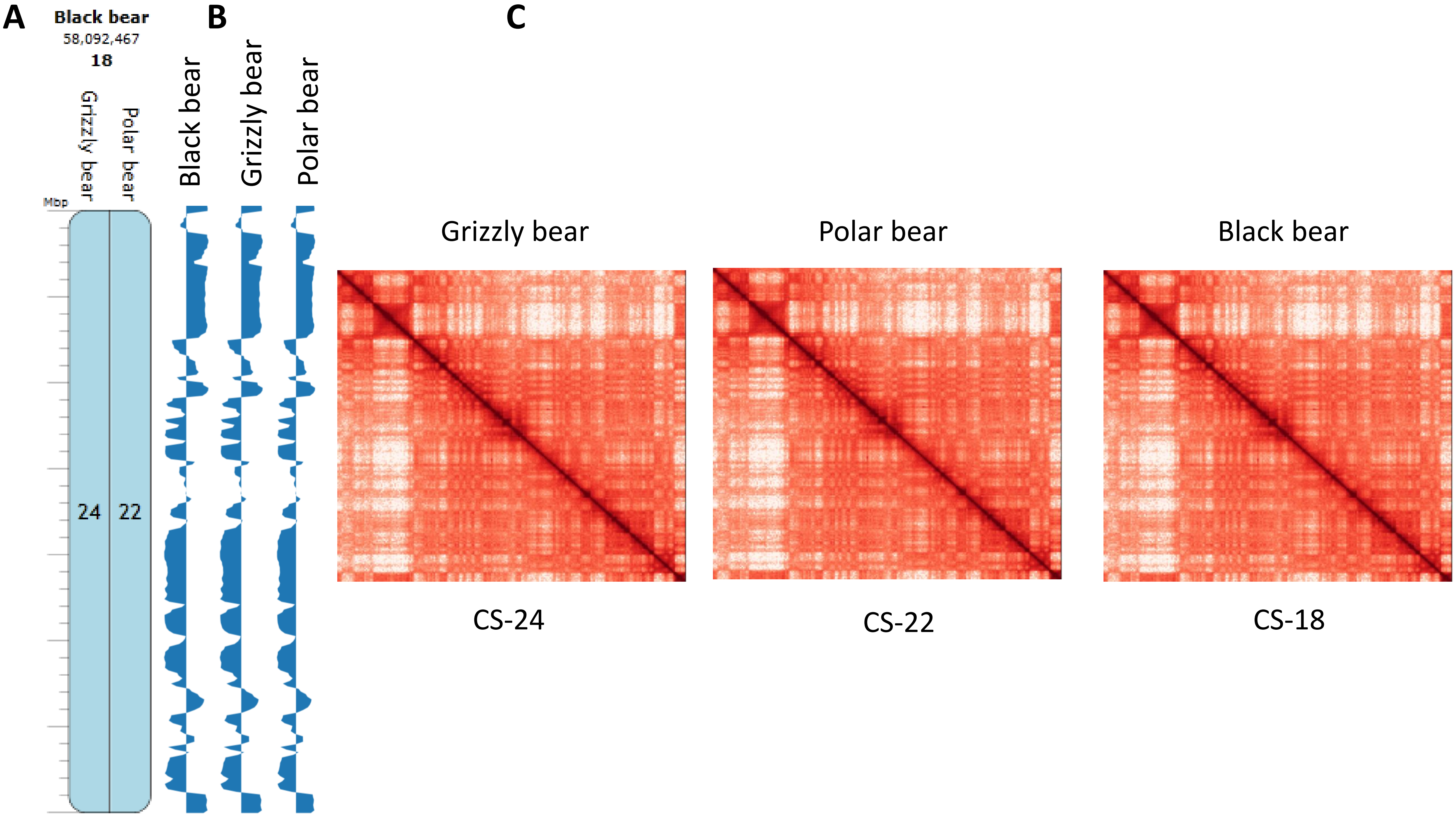
A
Black bear
58,092,467
18
Grizzly bear
Polar bear
Mbp
24
22
B
Black bear
Grizzly bear
Polar bear
C
Grizzly bear
Polar bear
Black bear
CS-24
CS-22
CS-18

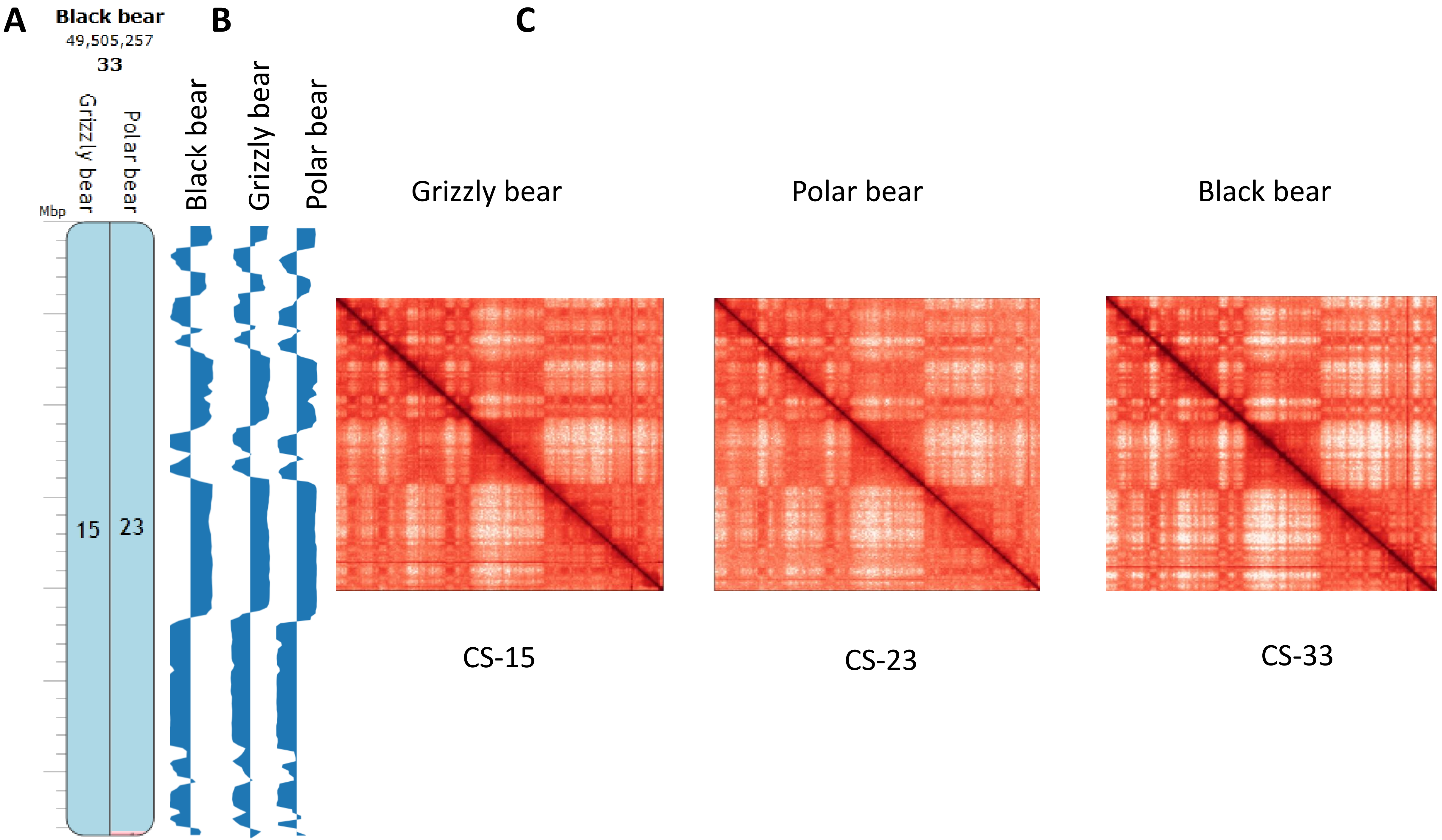
A
Black bear
49,505,257
33
Grizzly bear
Polar bear
Mbp
15
23
B
Black bear
Grizzly bear
Polar bear
C
Grizzly bear
Polar bear
Black bear
CS-15
CS-23
CS-33

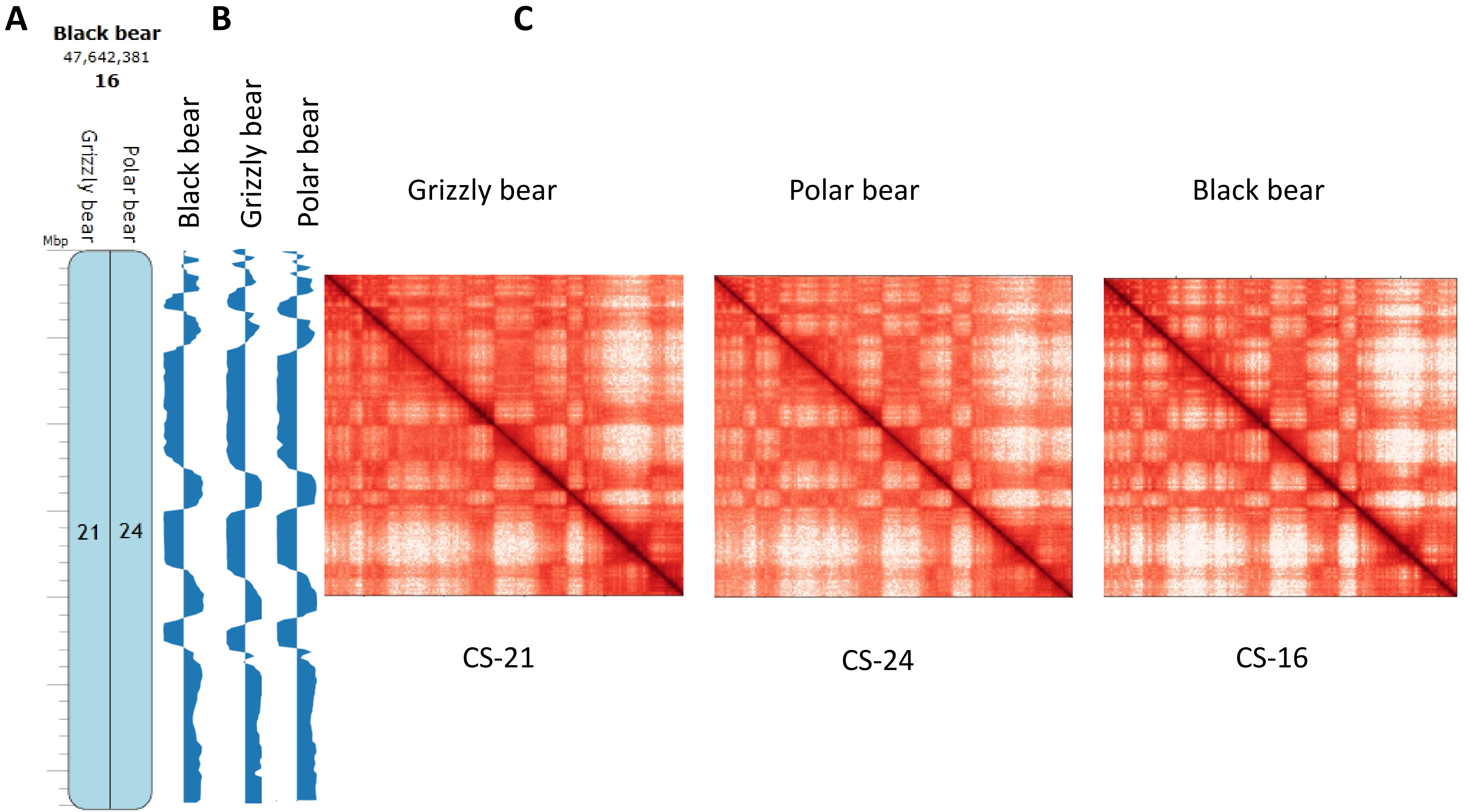
A
Black bear
47,642,381
16
Grizzly bear
Polar bear
Mbp
21
24
B
Black bear
Grizzly bear
Polar bear
C
Grizzly bear
Polar bear
Black bear
CS-21
CS-24
CS-16

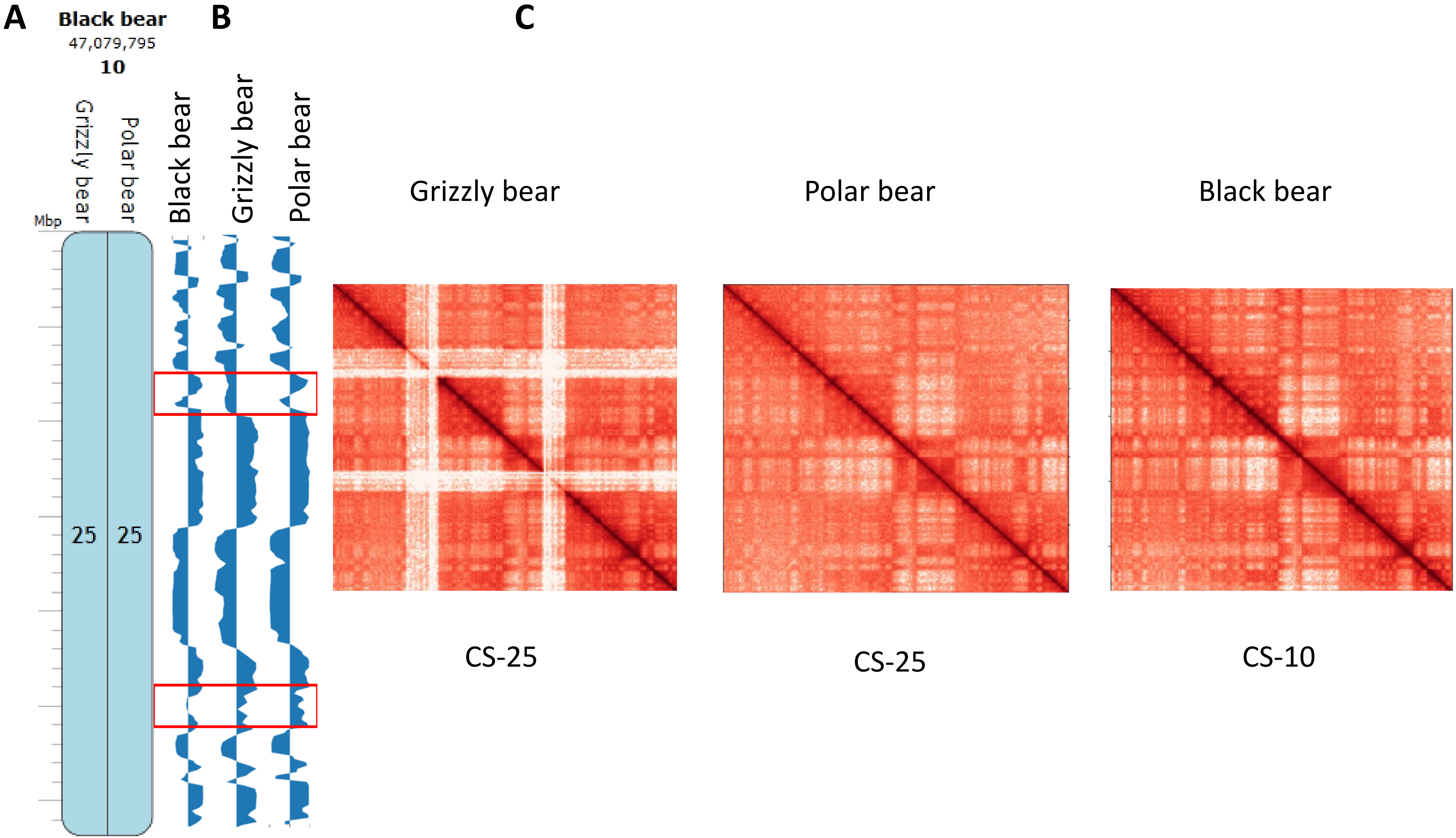

A
Black bear
47,079,795
10
Grizzly bear
Polar bear
Mbp
25
25
B
Black bear
Grizzly bear
Polar bear
C
Grizzly bear
Polar bear
Black bear
CS-25
CS-25
CS-10

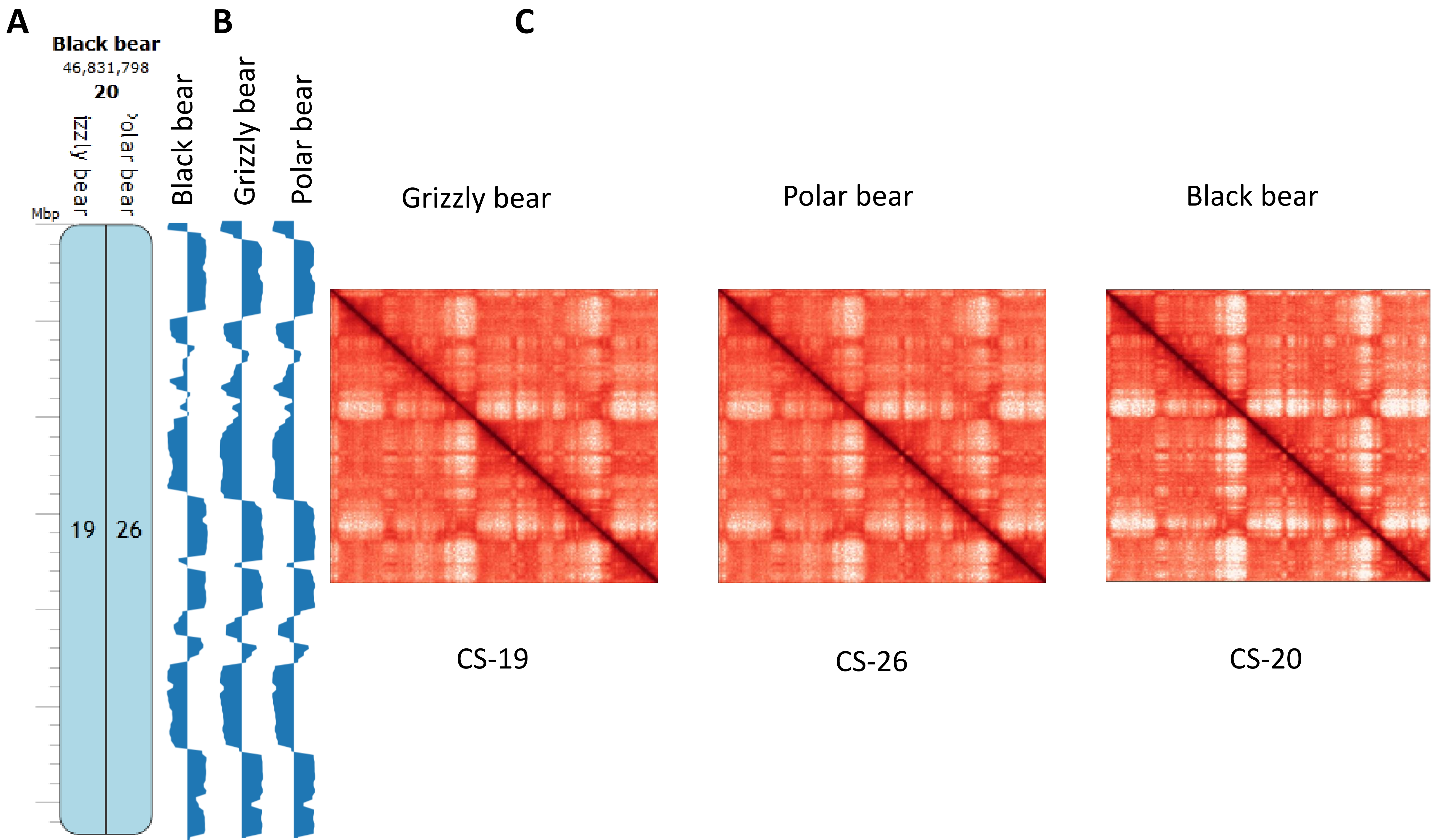
A
Black bear
46,831,798
20
izzly bear
Polar bear
Mbp
19
26
B
Black bear
Grizzly bear
Polar bear
C
Grizzly bear
Polar bear
Black bear
CS-19
CS-26
CS-20

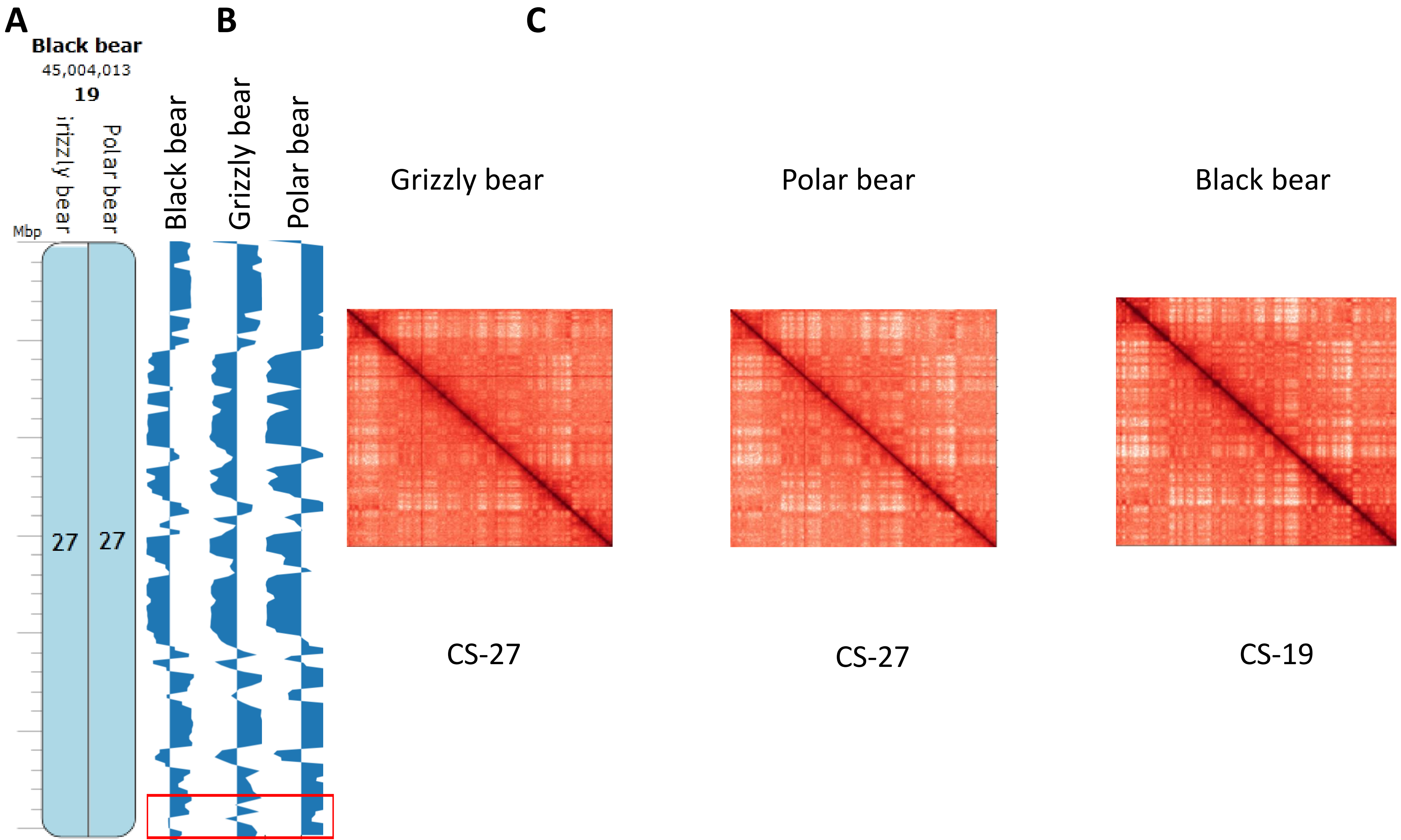
A
Black bear
45,004,013
19
Grizzly bear
Polar bear
Mbp
27
27
B
Black bear
Grizzly bear
Polar bear
C
Grizzly bear
Polar bear
Black bear
CS-27
CS-27
CS-19

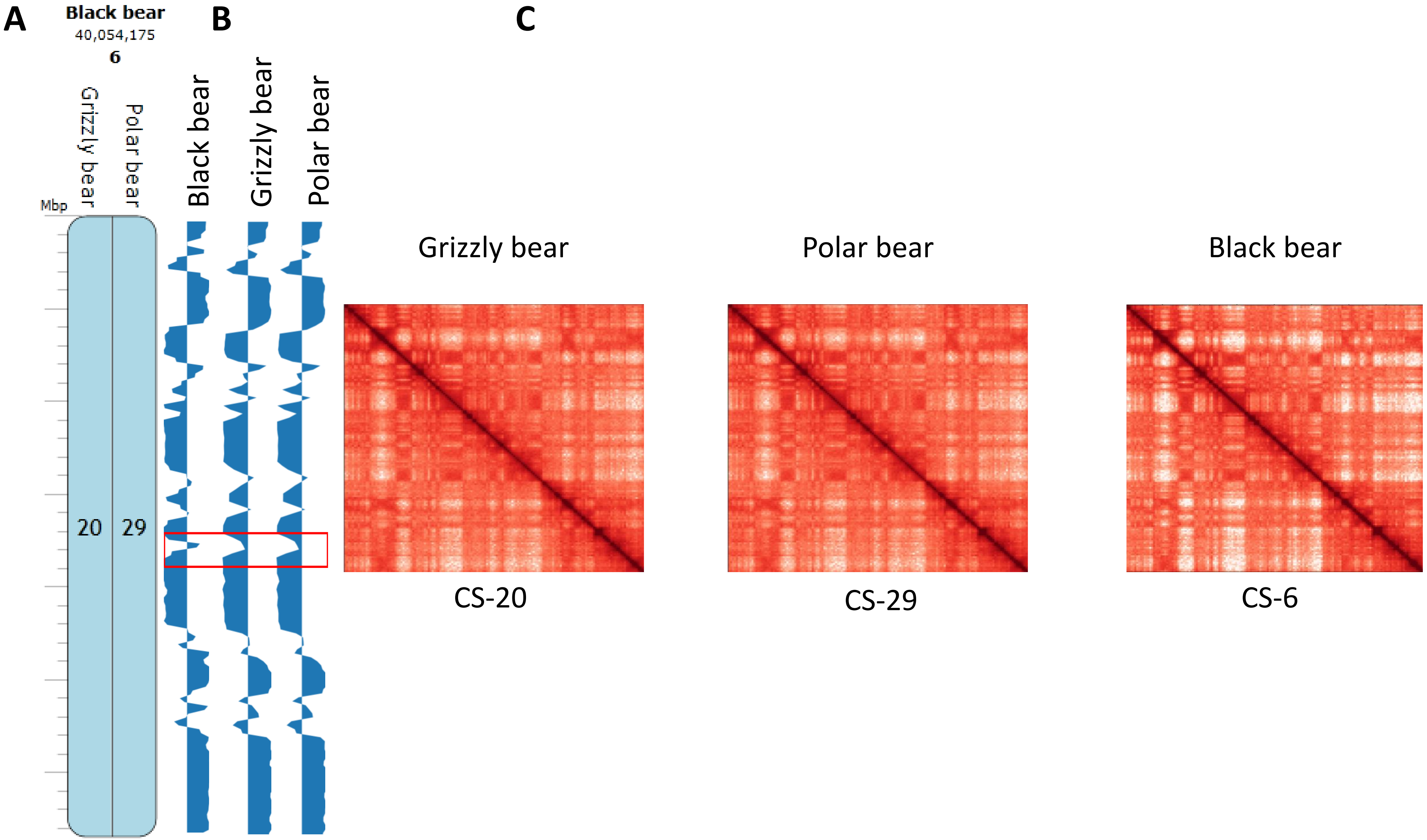
A
Black bear
40,054,175
6
Grizzly bear
Polar bear
Mbp
20
29
B
Black bear
Grizzly bear
Polar bear
C
Grizzly bear
Polar bear
Black bear
CS-20
CS-29
CS-6

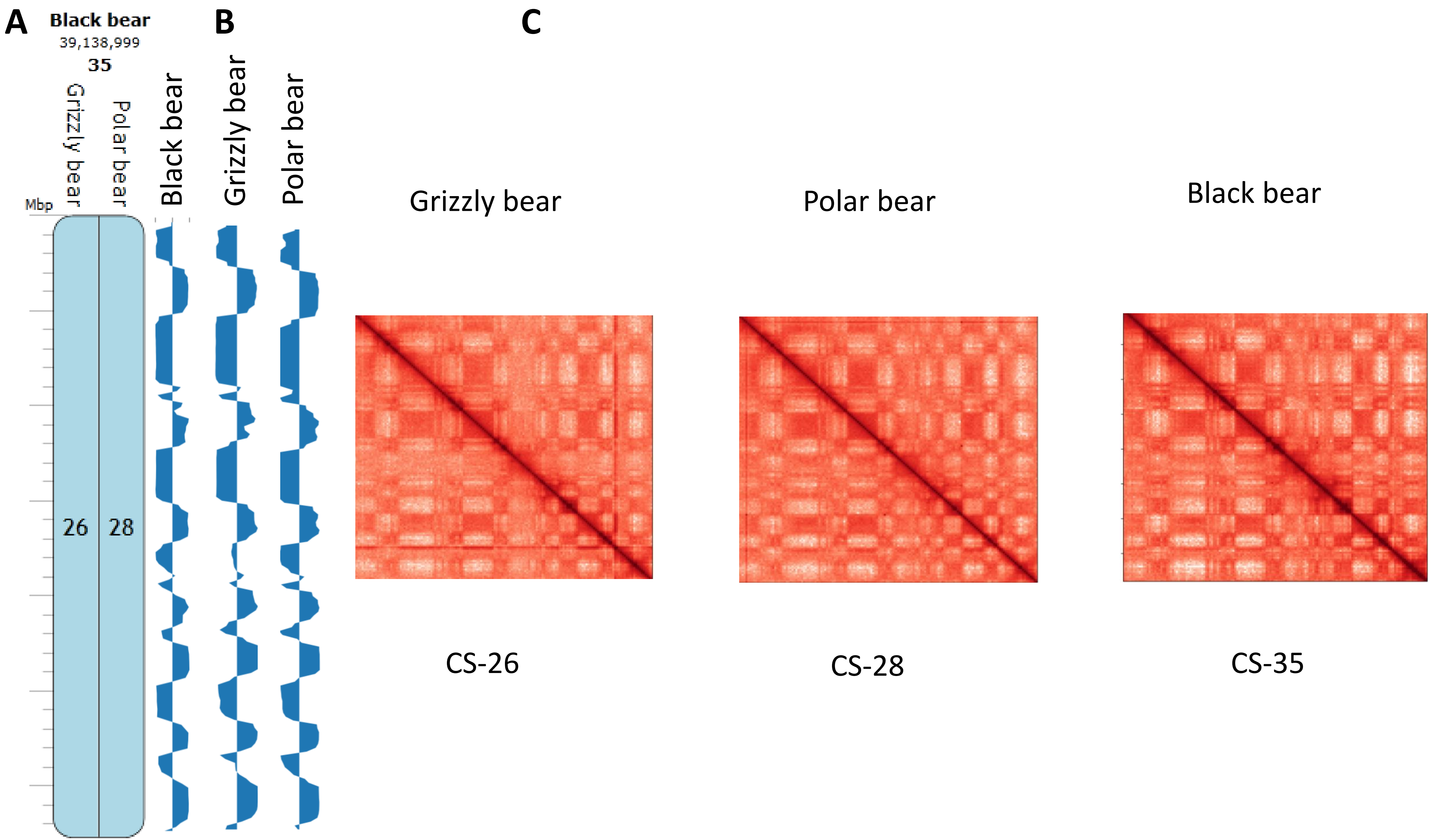
A
Black bear
39,138,999
35
Grizzly bear
Polar bear
Mbp
26
28
B
Black bear
Grizzly bear
Polar bear
C
Grizzly bear
Polar bear
Black bear
CS-26
CS-28
CS-35

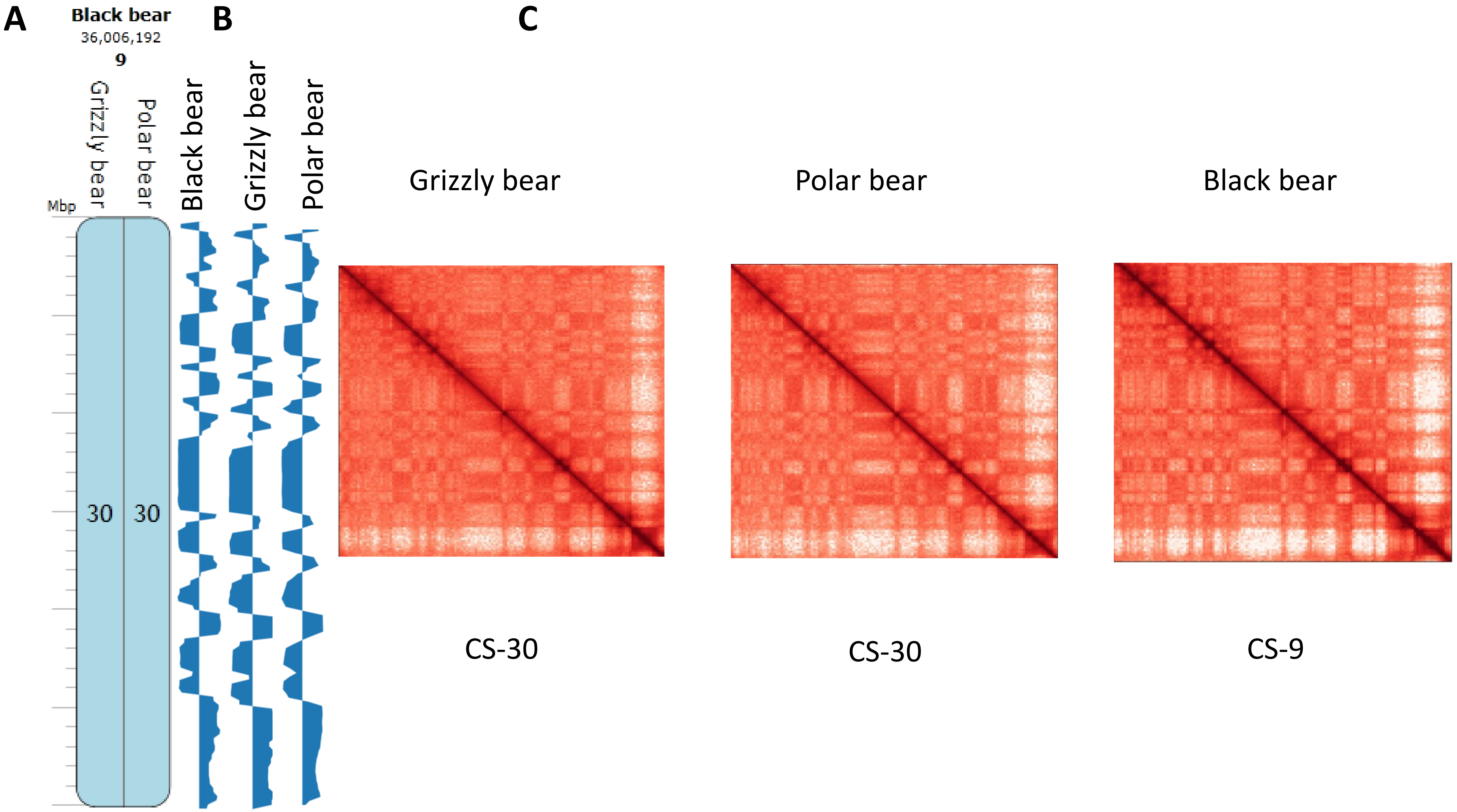
A
Black bear
36,006,192
9
Grizzly bear
Polar bear
Mbp
30
30
B
Black bear
Grizzly bear
Polar bear
C
Grizzly bear
Polar bear
Black bear
CS-30
CS-30
CS-9

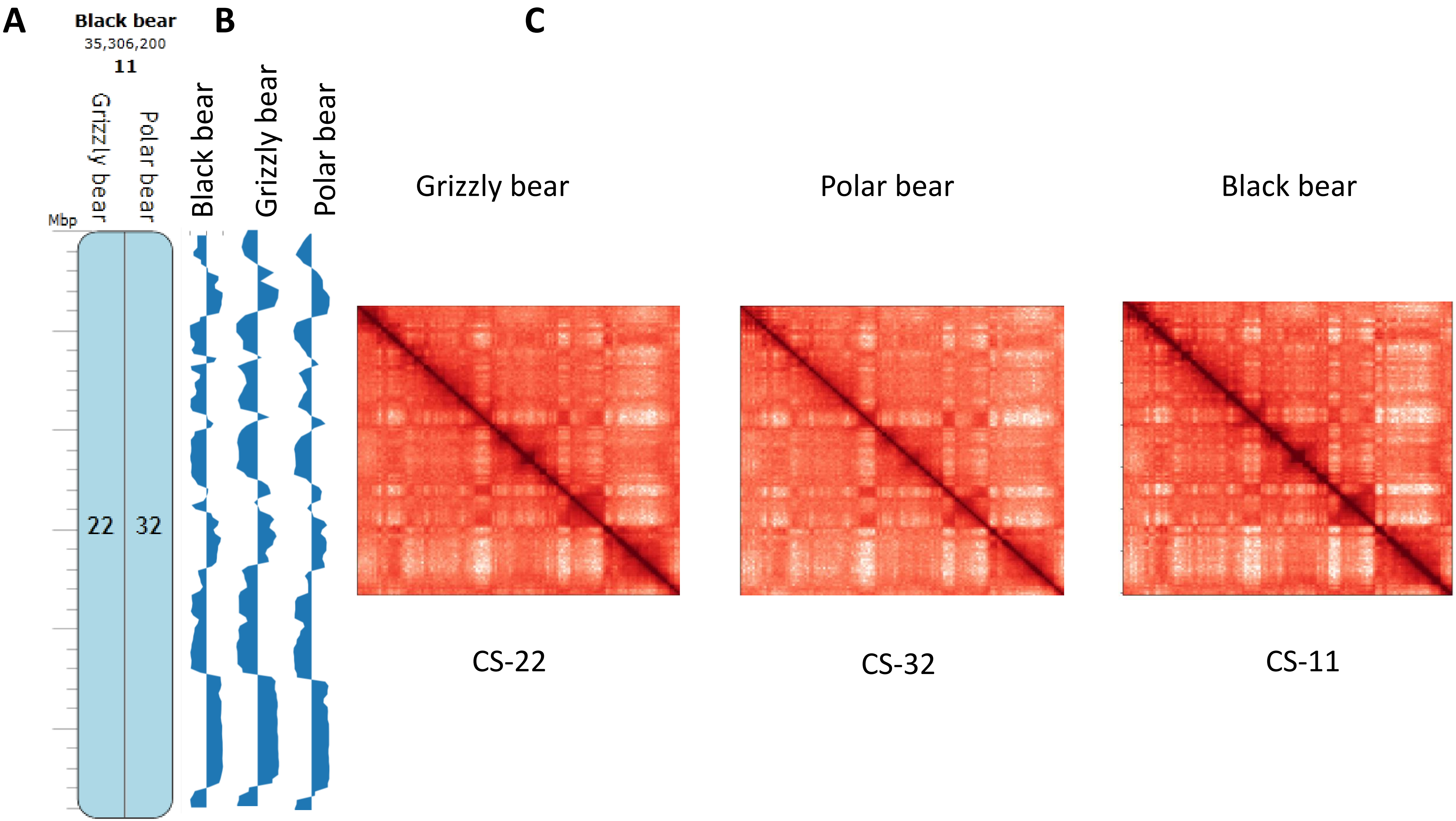

A
Black bear
35,306,200
11
Grizzly bear
Polar bear
Mbp
22
32
B
Black bear
Grizzly bear
Polar bear
C
Grizzly bear
Polar bear
Black bear
CS-22
CS-32
CS-11

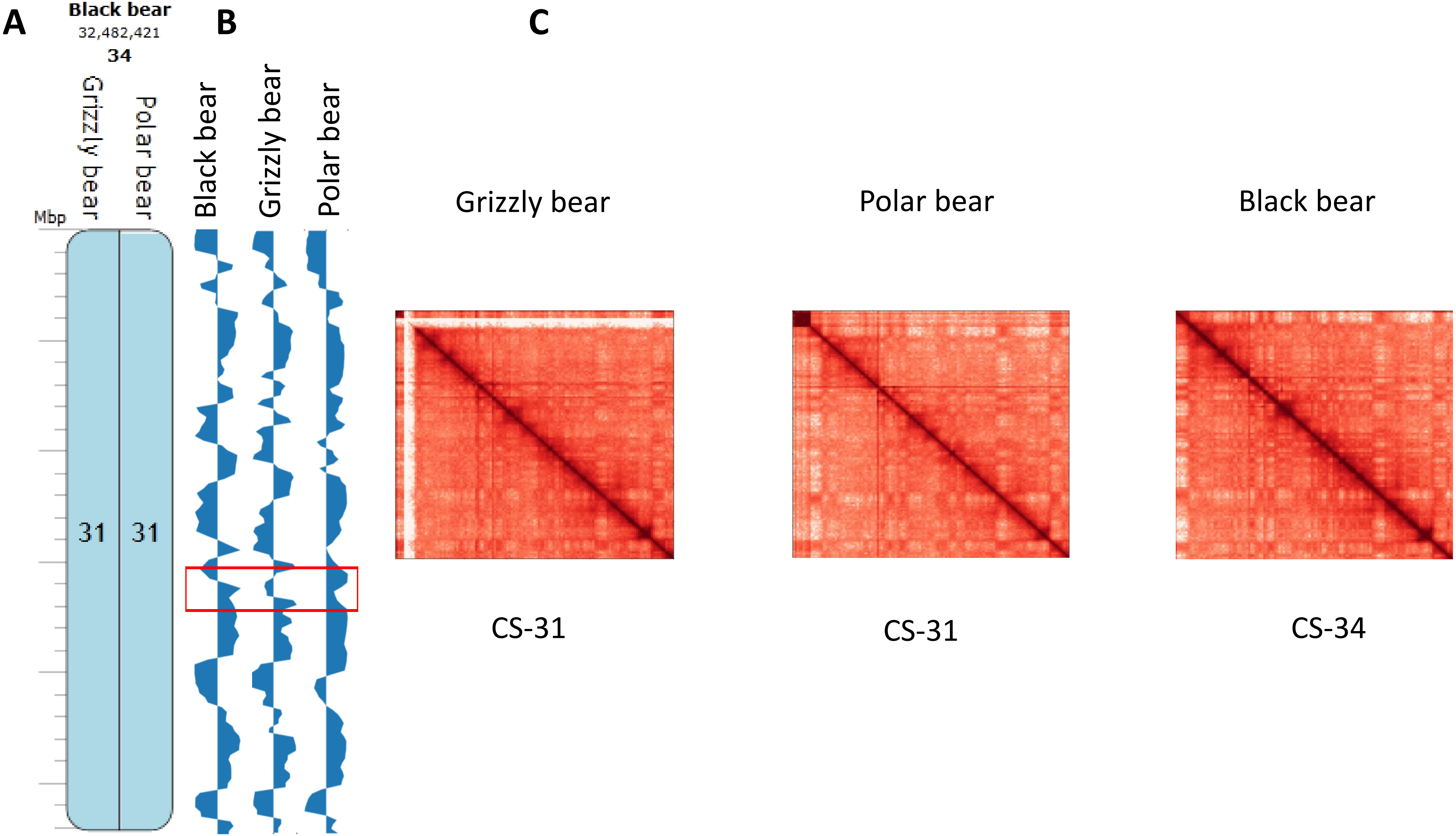
A
Black bear
32,482,421
34
Grizzly bear
Polar bear
Mbp
31
31
B
Black bear
Grizzly bear
Polar bear
C
Grizzly bear
Polar bear
Black bear
CS-31
CS-31
CS-34

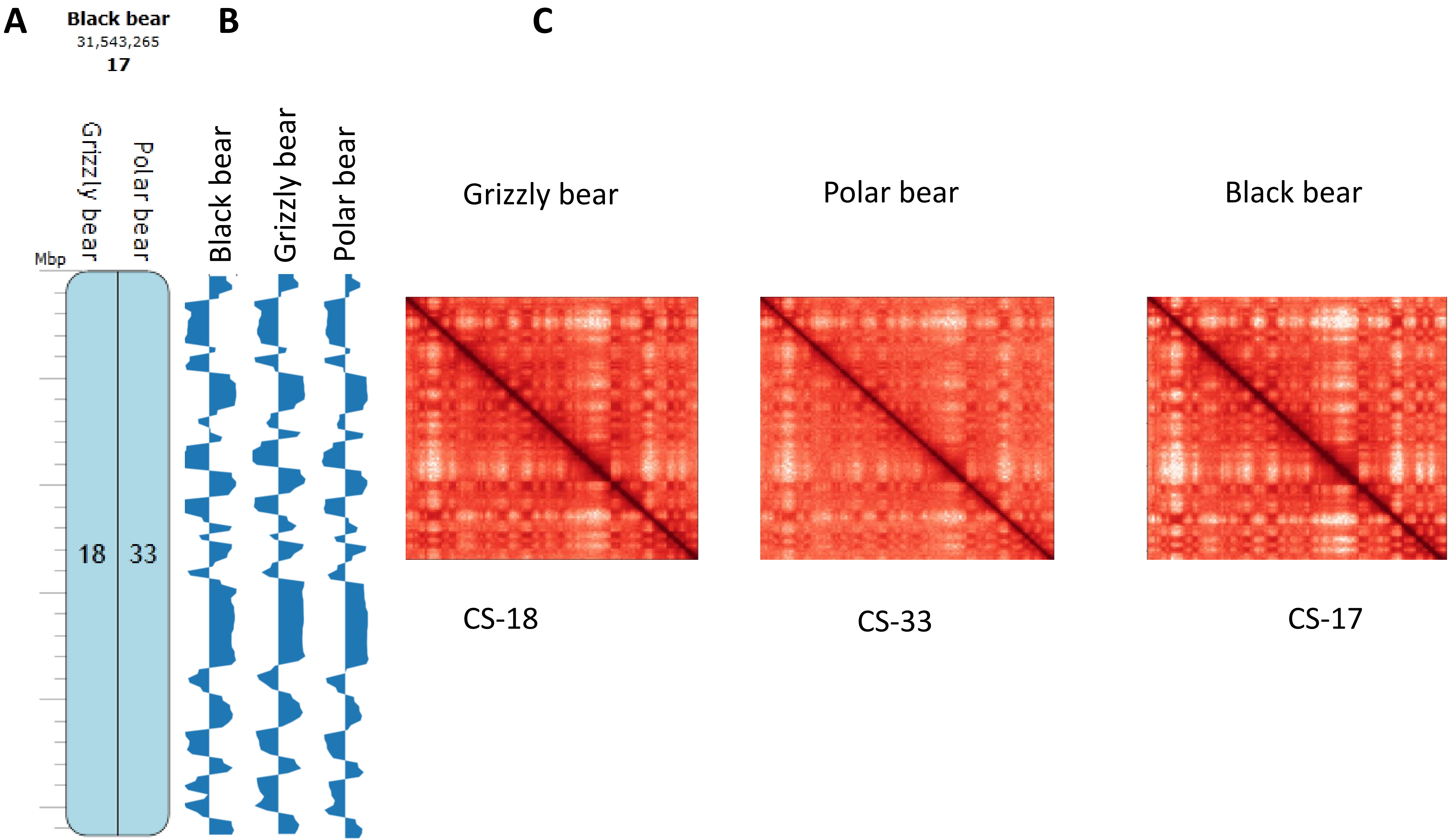
A
Black bear
31,543,265
17
Grizzly bear
Polar bear
Mbp
18
33
B
Black bear
Grizzly bear
Polar bear
C
Grizzly bear
Polar bear
Black bear
CS-18
CS-33
CS-17

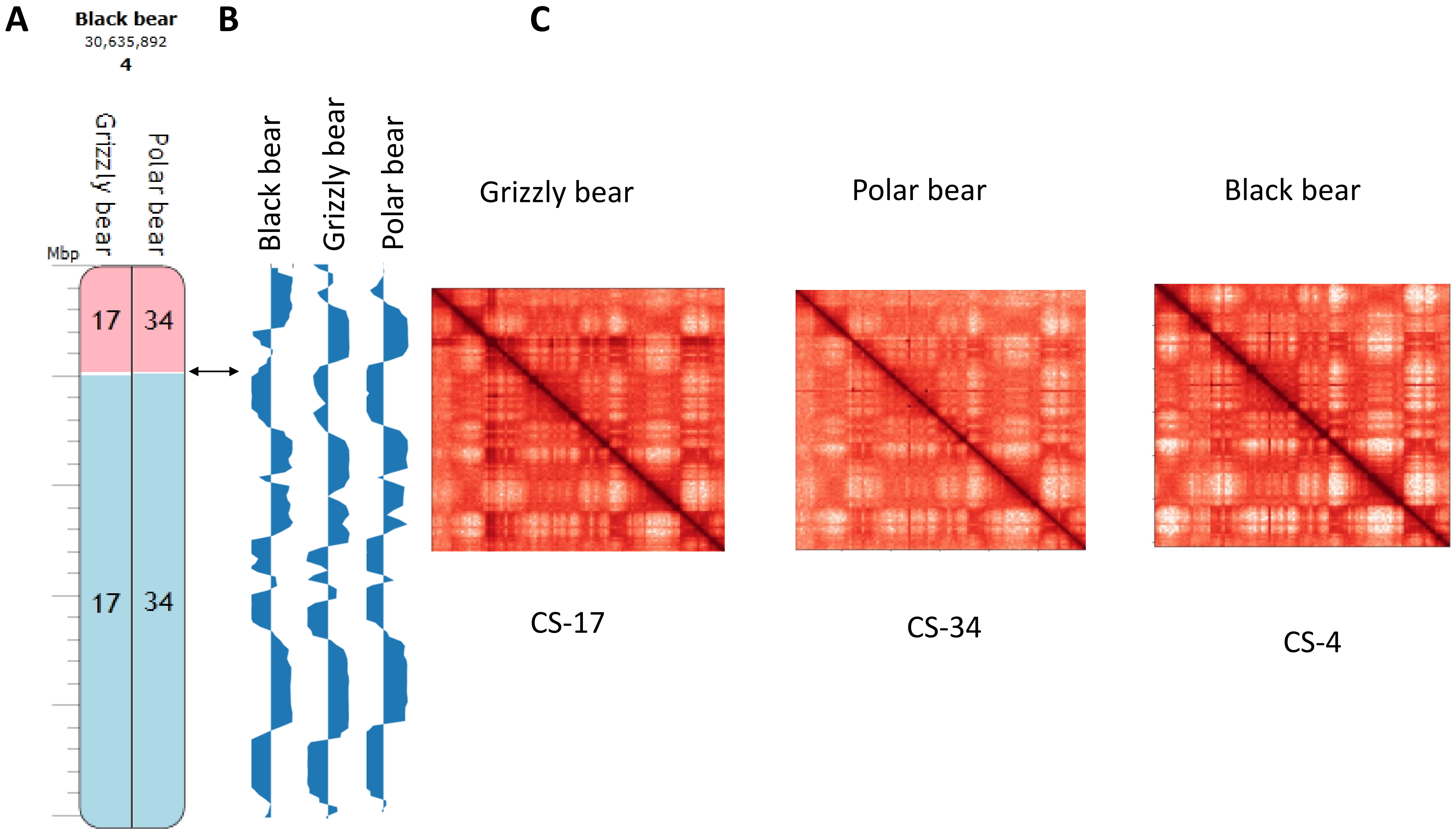

A
Black bear
30,635,892
4
Grizzly bear
Polar bear
Mbp
17
34
17
34
B
Black bear
Grizzly bear
Polar bear
C
Grizzly bear
Polar bear
Black bear
CS-17
CS-34
CS-4

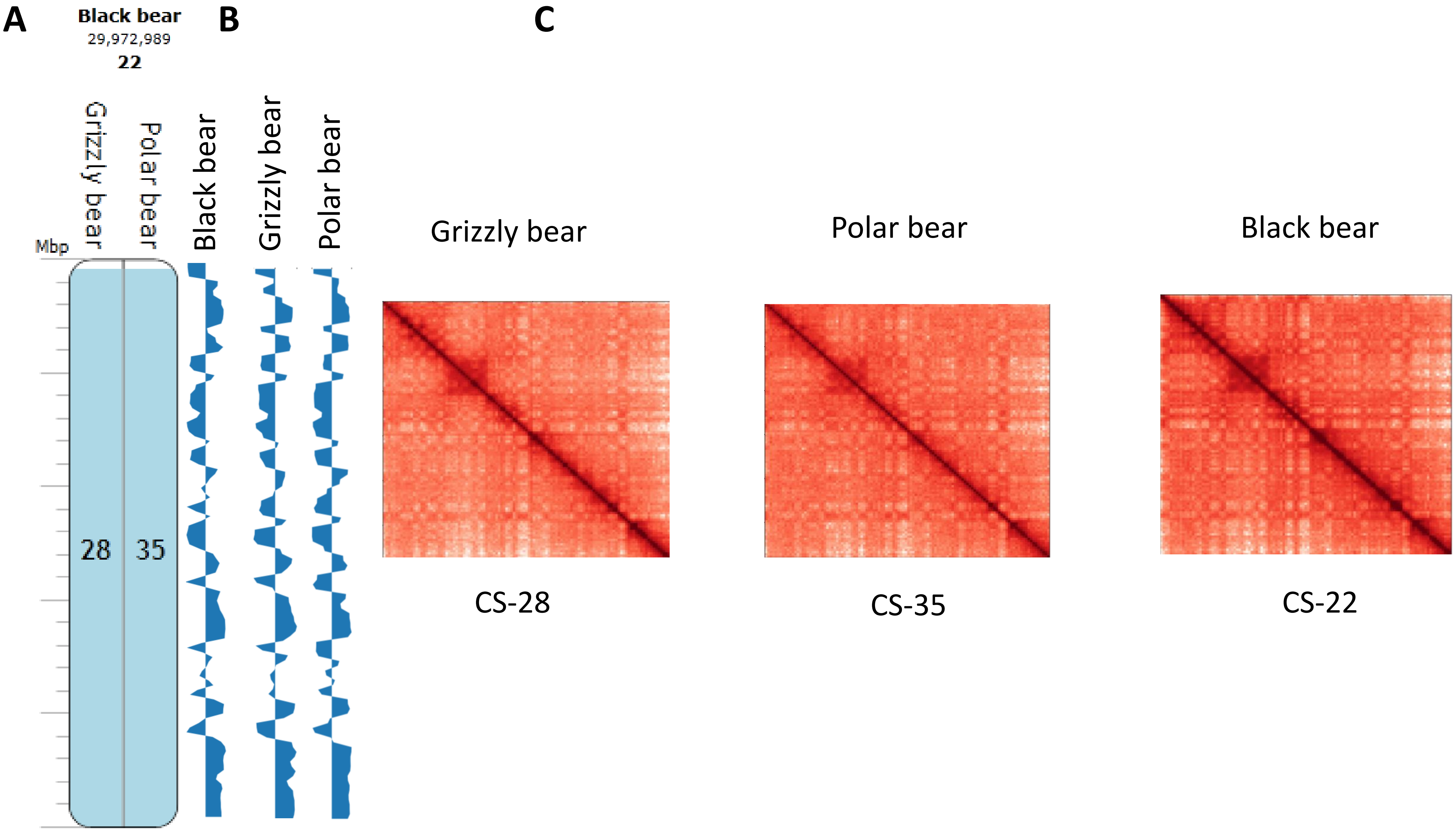
A
Black bear
29,972,989
22
Grizzly bear
Polar bear
Mbp
28
35
B
Black bear
Grizzly bear
Polar bear
C
Grizzly bear
Polar bear
Black bear
CS-28
CS-35
CS-22

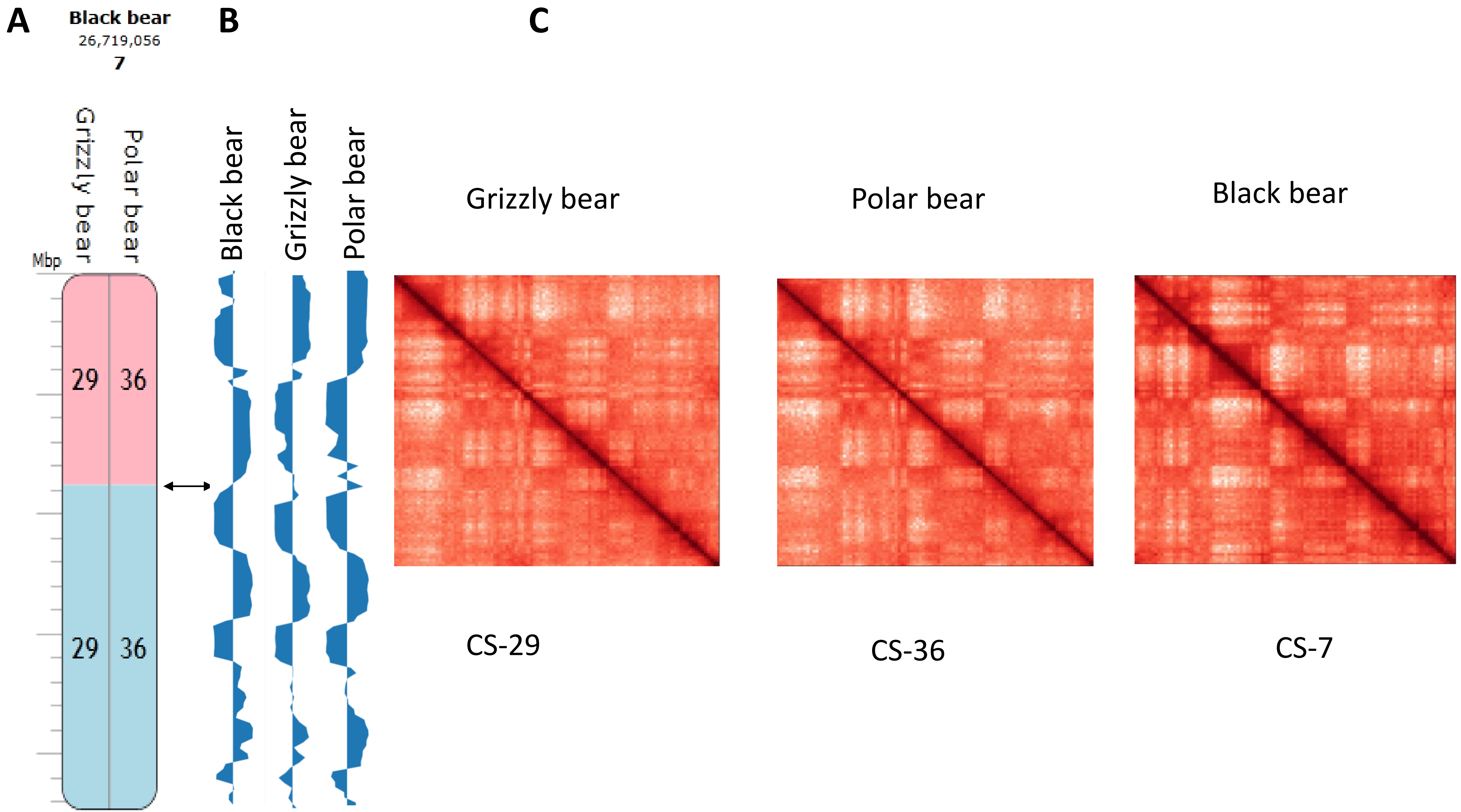
A
Black bear
26,719,056
7
Grizzly bear
Polar bear
Mbp
29
36
29
36
B
Black bear
Grizzly bear
Polar bear
C
Grizzly bear
Polar bear
Black bear
CS-29
CS-36
CS-7

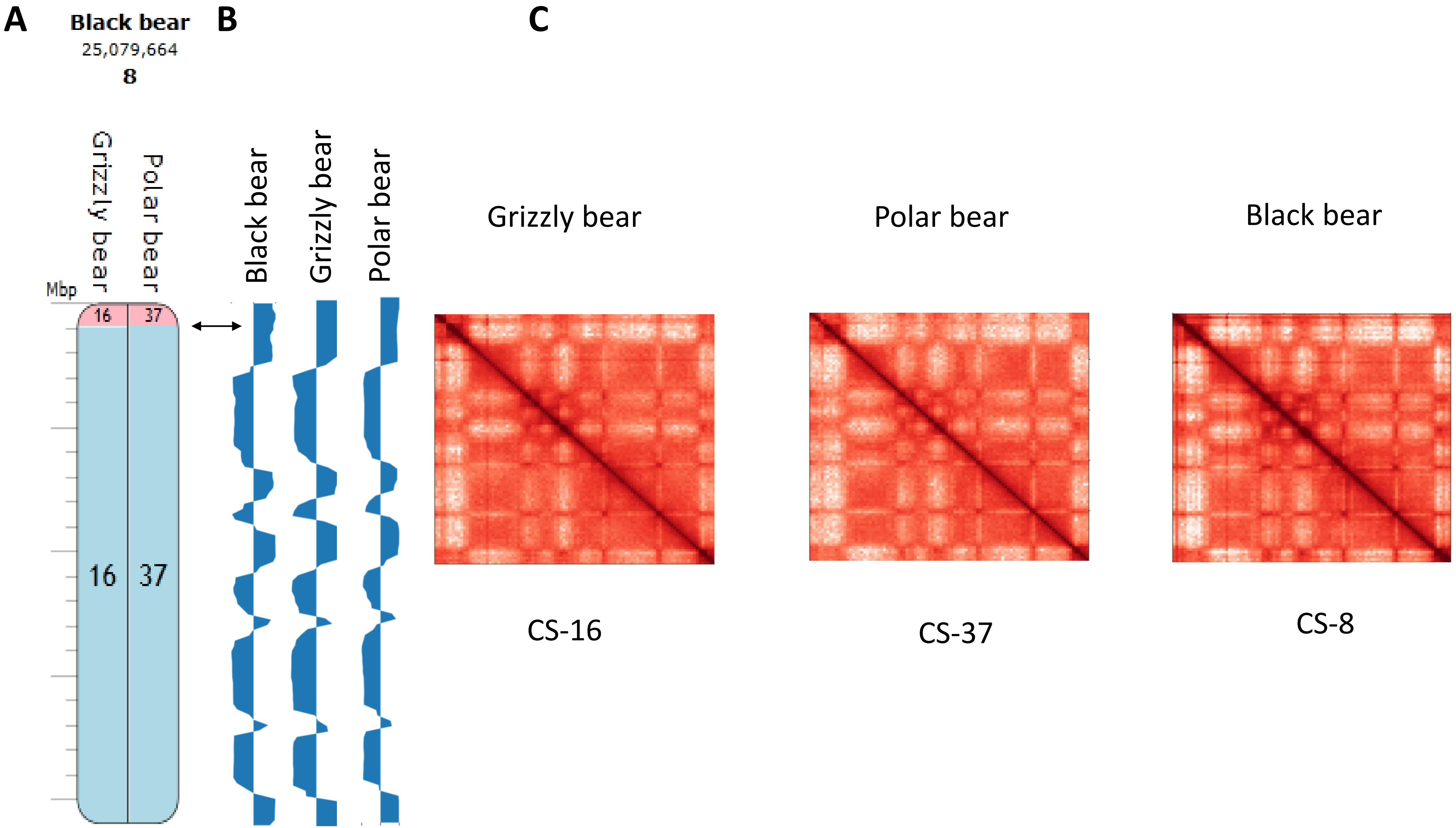
A
Black bear
25,079,664
8
Grizzly bear
Polar bear
Mbp
16
37
16
37
B
Black bear
Grizzly bear
Polar bear
C
Grizzly bear
Polar bear
Black bear
CS-16
CS-37
CS-8

Figure S4. Comparative chromatin conformation analysis in ursids.

Black bear, grizzly bear and polar bear C-scaffolds orthologous to the rest of black bear C-scaffolds. (A) Homologous synteny blocks of the three ursids visualized in Evolution Highway at 300 kb resolution. Blue indicates same sequence orientation as the reference genome. Pink depicts chromosome inversions. Numbers represent the scaffold identifier of the target species. (B) Eigenvector values of each species aligned to the black bear reference genome at 500 kb resolution. Red boxes highlight compartment shift. (C) Juicer plots of C-scaffolds for the three ursids. Color intensity reflects the frequency of interactions between pairs of loci on the C-scaffolds (range 1-1,000 for each map). Alignment coordinates can be found in SI Dataset S7.

Cat Chr	Puma	Cheetah	Leopard	Tiger	Clouded leopard	Dingo	Wild dog	Red fox	Black bear	Grizzly bear	Polar bear
**2*n*** 38	38	38	38	38	38	78	78	34	74	74	74
A1	1	1	1	1	1	9	9	8	3	3	3
A2	1	1	1	1	1	4	4	3	2	2	2
A3	1	1	1	1	1	4	4	4	2	2	2
B1	1	1	1	1	1	8	8	7	3	3	3
B2	1	1	1	1	1	3	3	2	3	3	3
B3	1	1	1	1	1	4	4	4	5	5	5
B4	1	1	1	1	1	4	4	4	3	3	3
C1	1	1	1	1	1	9	9	8	4	4	4
C2	1	1	1	1	1	4	4	4	2	2	2
D1	1	1	1	1	1	3	3	3	2	2	2
D2	1	1	1	1	1	3	3	3	1	1	1
D3	1	1	1	1	1	3	3	3	2	2	2
D4	1	1	1	1	1	3	3	3	2	2	2
E1	1	1	1	1	1	2	2	2	2	2	2
E2	1	1	1	1	1	3	2	3	1	1	1
E3	1	1	1	1	1	1	1	1	1	1	1
F1	1	1	1	1	1	2	2	2	1	1	1
F2	1	1	1	1	1	3	3	2	1	1	1
X	1	1	1	1	1	1	1	1	1	1	1

Figure S5. Chromosome orthology grid.

Comparative orthology grid of C-scaffolds of felids, ursids, and canids. Number in each box represents the total number of chromosomal segments orthologous to each cat chromosome. Green represents 1:1 chromosome orthology of all species compared to the cat chromosomes. Light blue represents 1:1 full chromosome orthology of the canid species. Yellow represents 1:1 chromosome orthology of the bear species. Grey represents lack of 1:1 chromosome orthology.

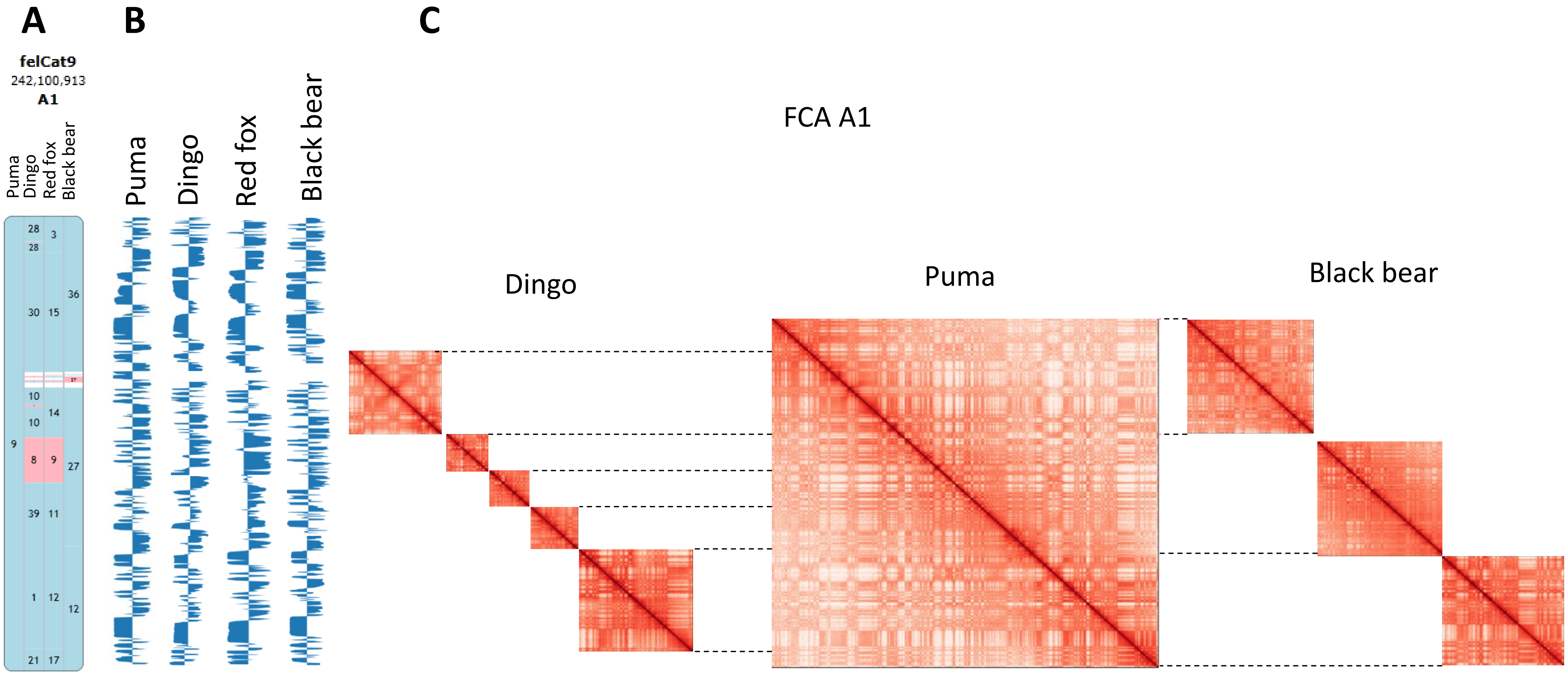

A
B
C
felCat9
242,100,913
A1
Puma
Dingo
Red fox
Black bear
28
28
3
36
30
15
10
10
14
9
8
9
27
39
11
1
12
12
21
17
Puma
Dingo
Red fox
Black bear
FCA A1
Dingo
Puma
Black bear

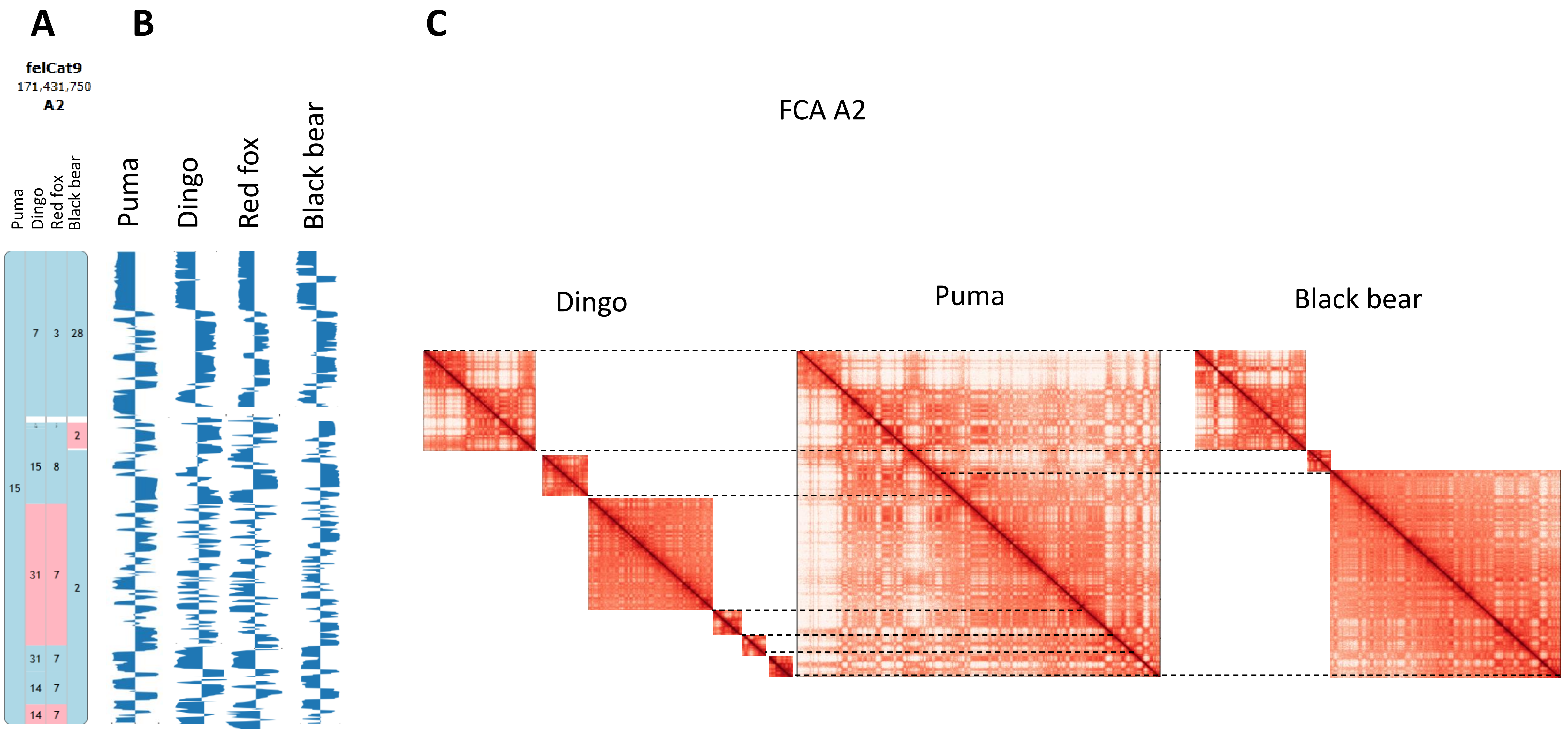
A
B
C
felCat9
171,431,750
A2
Puma
Dingo
Red fox
Black bear
7
3
28
2
15
8
15
31
7
2
31
7
14
7
14
7
Puma
Dingo
Red fox
Black bear
FCA A2
Dingo
Puma
Black bear

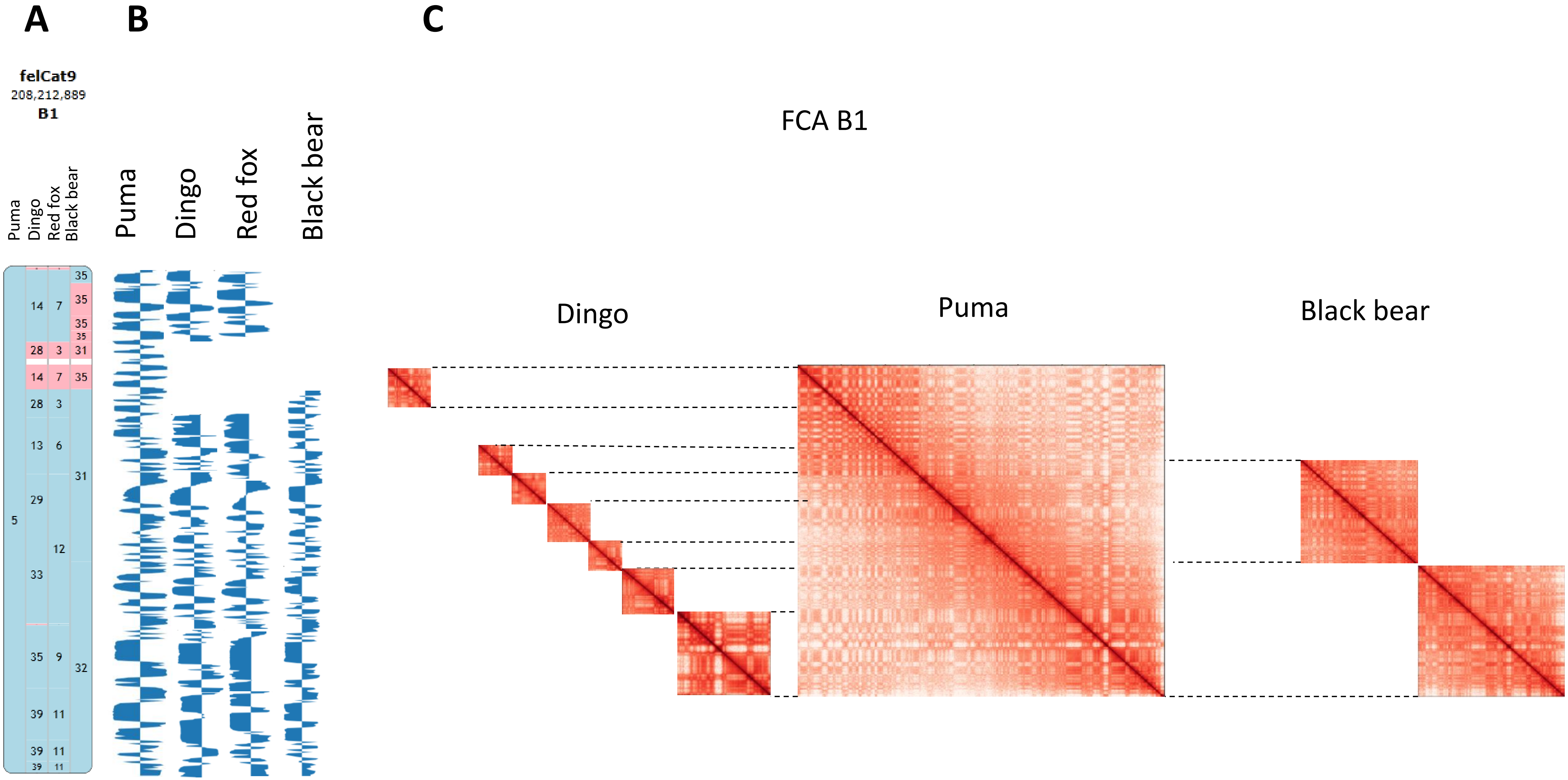

A
B
C
felCat9
208,212,889
B1
Puma
Dingo
Red fox
Black bear
FCA B1
Dingo
Puma
Black bear

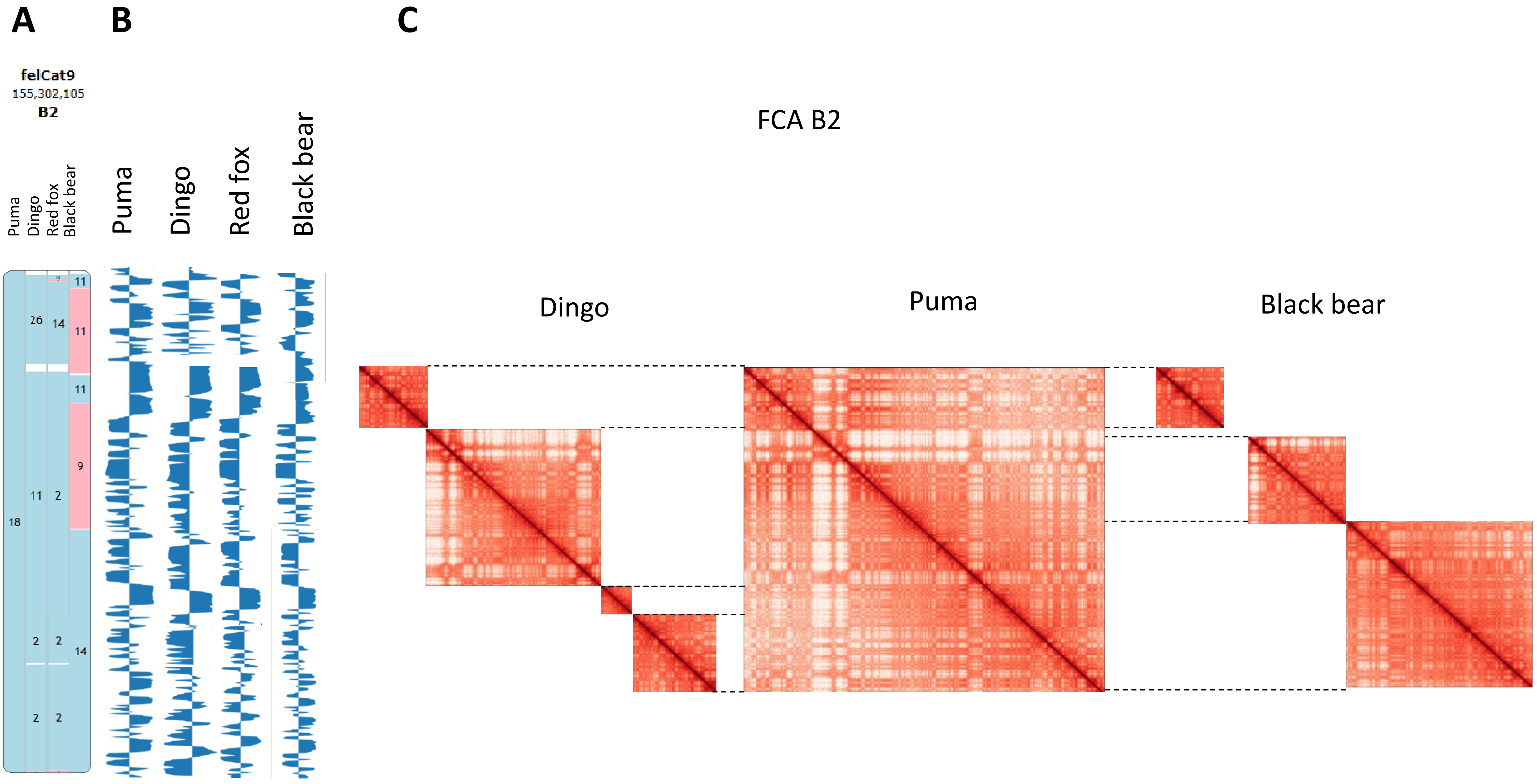
A
B
C
felCat9
155,302,105
B2
Puma
Dingo
Red fox
Black bear
FCA B2
Dingo
Puma
Black bear

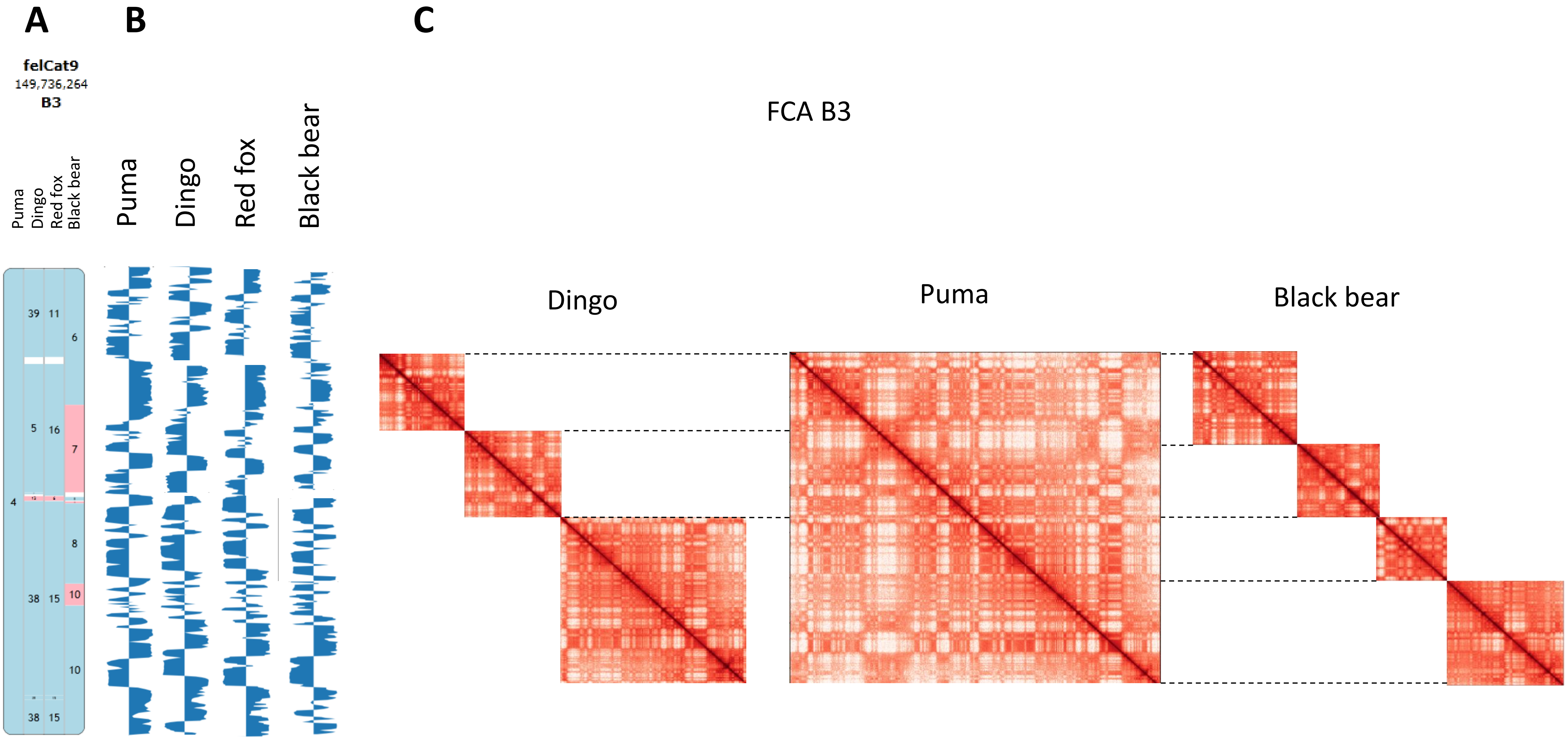
A
B
C
felCat9
149,736,264
B3
Puma
Dingo
Red fox
Black bear
39
11
6
5
16
7
4
8
38
15
10
10
38
15
Puma
Dingo
Red fox
Black bear
FCA B3
Dingo
Puma
Black bear

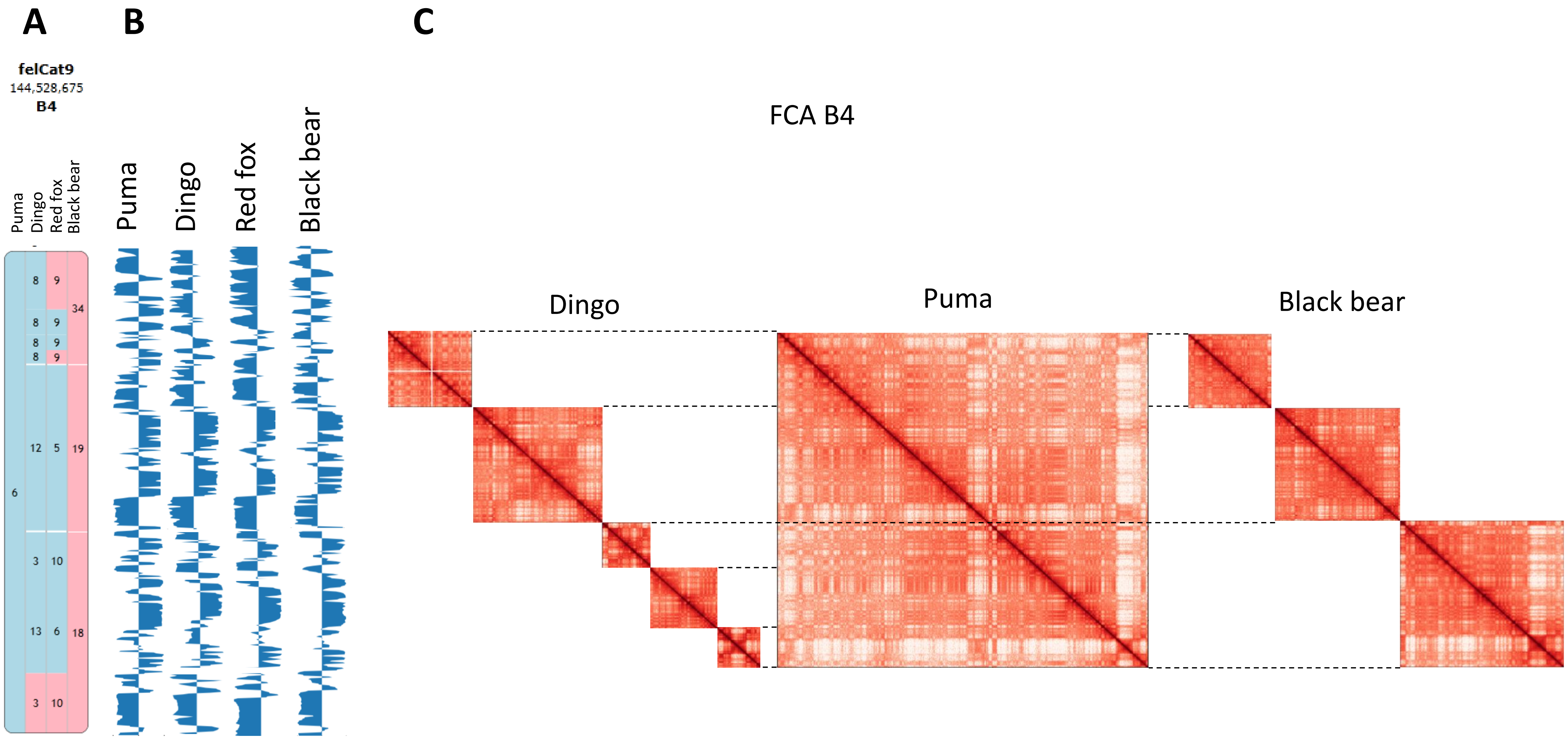
A
B
C
felCat9
144,528,675
B4
Puma
Dingo
Red fox
Black bear
8
9
34
8
9
8
9
8
9
12
5
19
6
3
10
13
6
18
3
10
Puma
Dingo
Red fox
Black bear
FCA B4
Dingo
Puma
Black bear

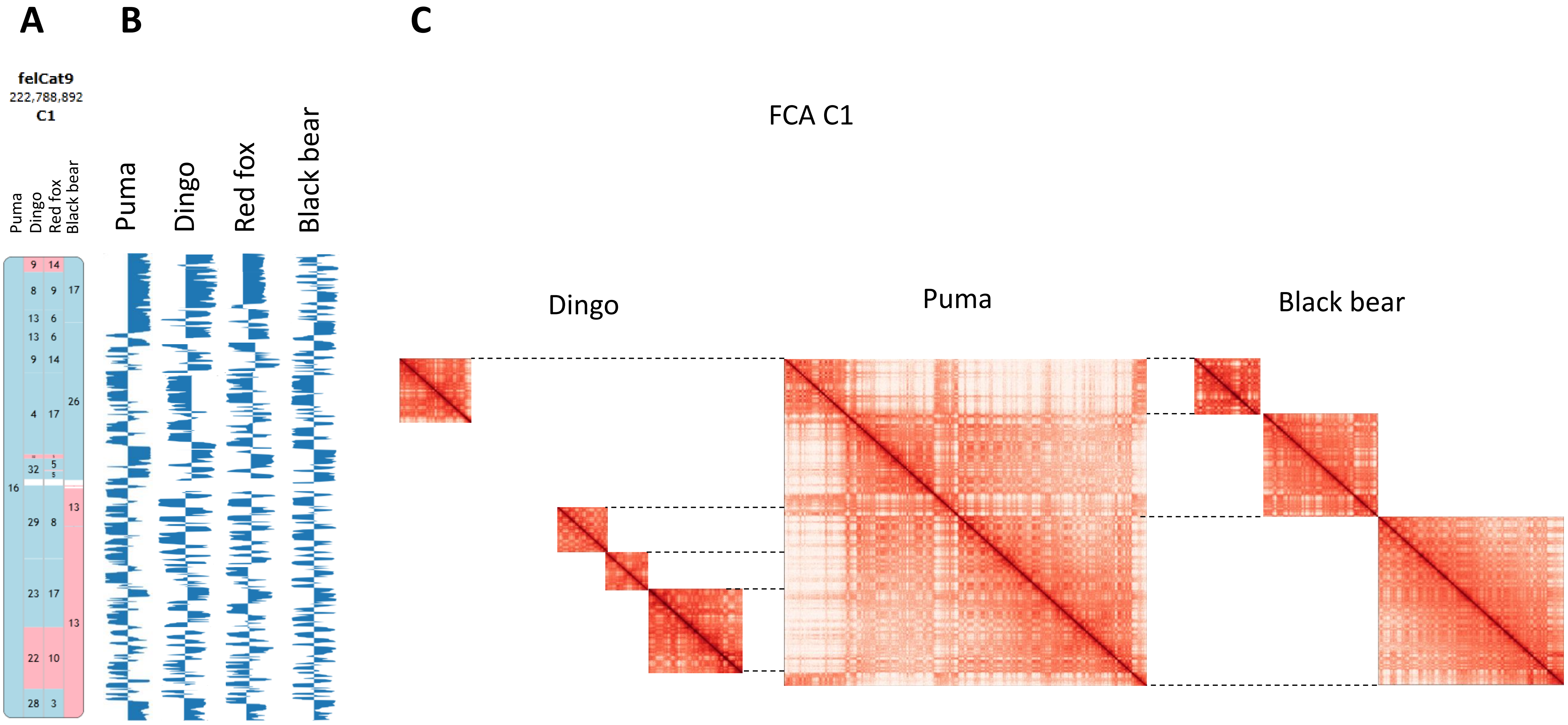
A
B
C
felCat9
222,788,892
C1
Puma
Dingo
Red fox
Black bear
9
14
8
9
17
13
6
13
6
9
14
26
4
17
32
5
16
13
29
8
23
17
13
22
10
28
3
Puma
Dingo
Red fox
Black bear
FCA C1
Dingo
Puma
Black bear

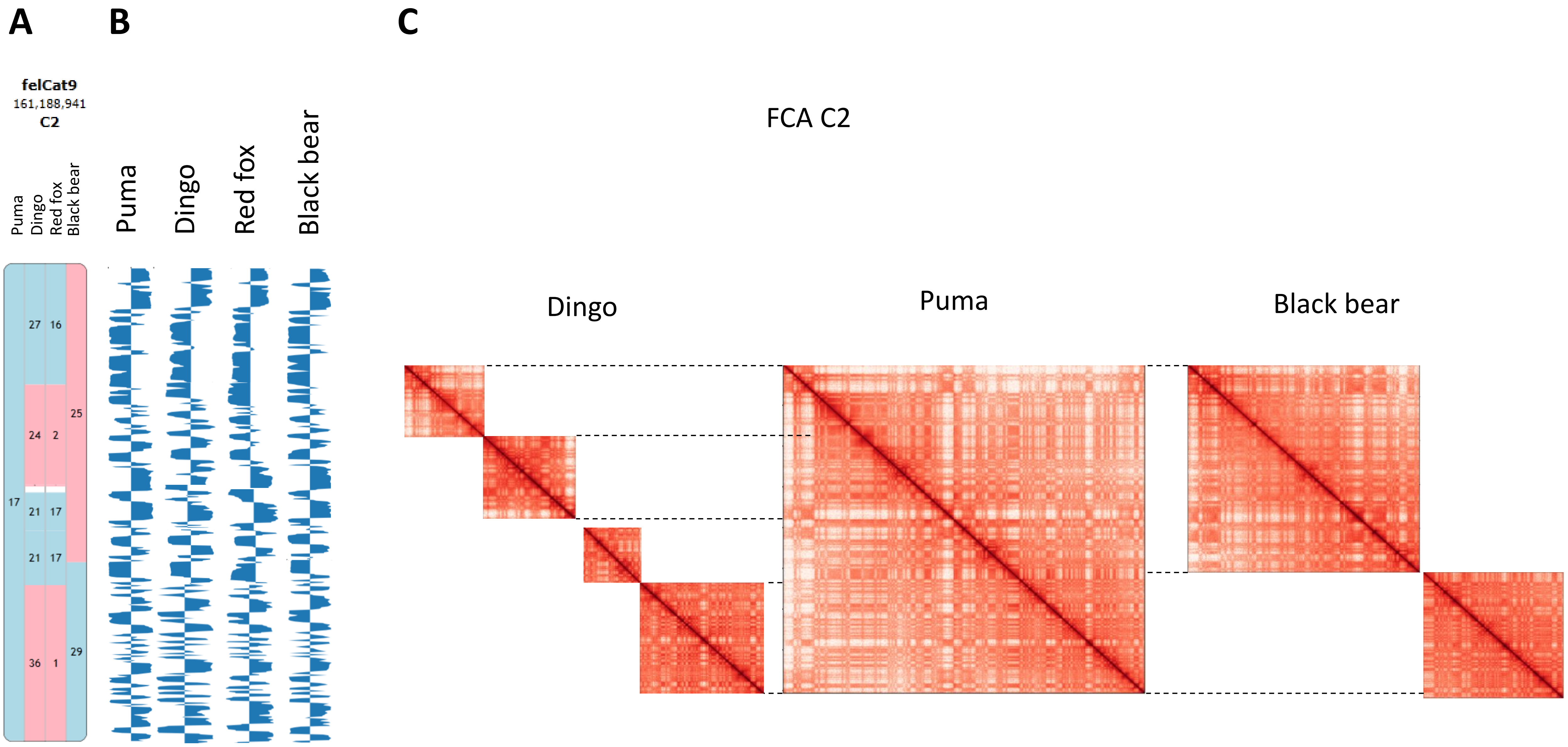
A
B
C
felCat9
161,188,941
C2
Puma
Dingo
Red fox
Black bear
27
16
25
24
2
17
21
17
21
17
36
1
29
Puma
Dingo
Red fox
Black bear
FCA C2
Dingo
Puma
Black bear

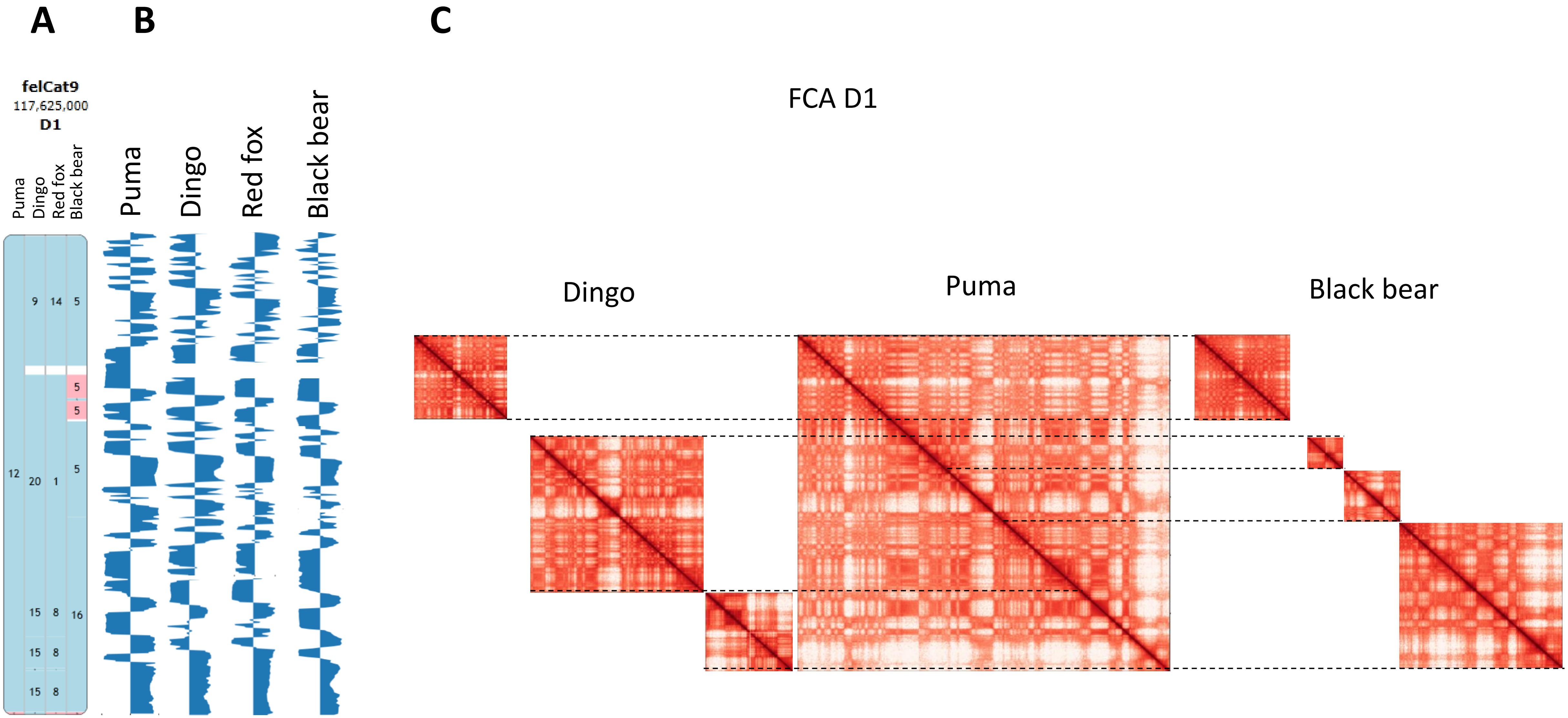
A
B
C
felCat9
117,625,000
D1
Puma
Dingo
Red fox
Black bear
9
14
5
5
5
5
12
20
1
15
8
16
15
8
15
8
Puma
Dingo
Red fox
Black bear
FCA D1
Dingo
Puma
Black bear

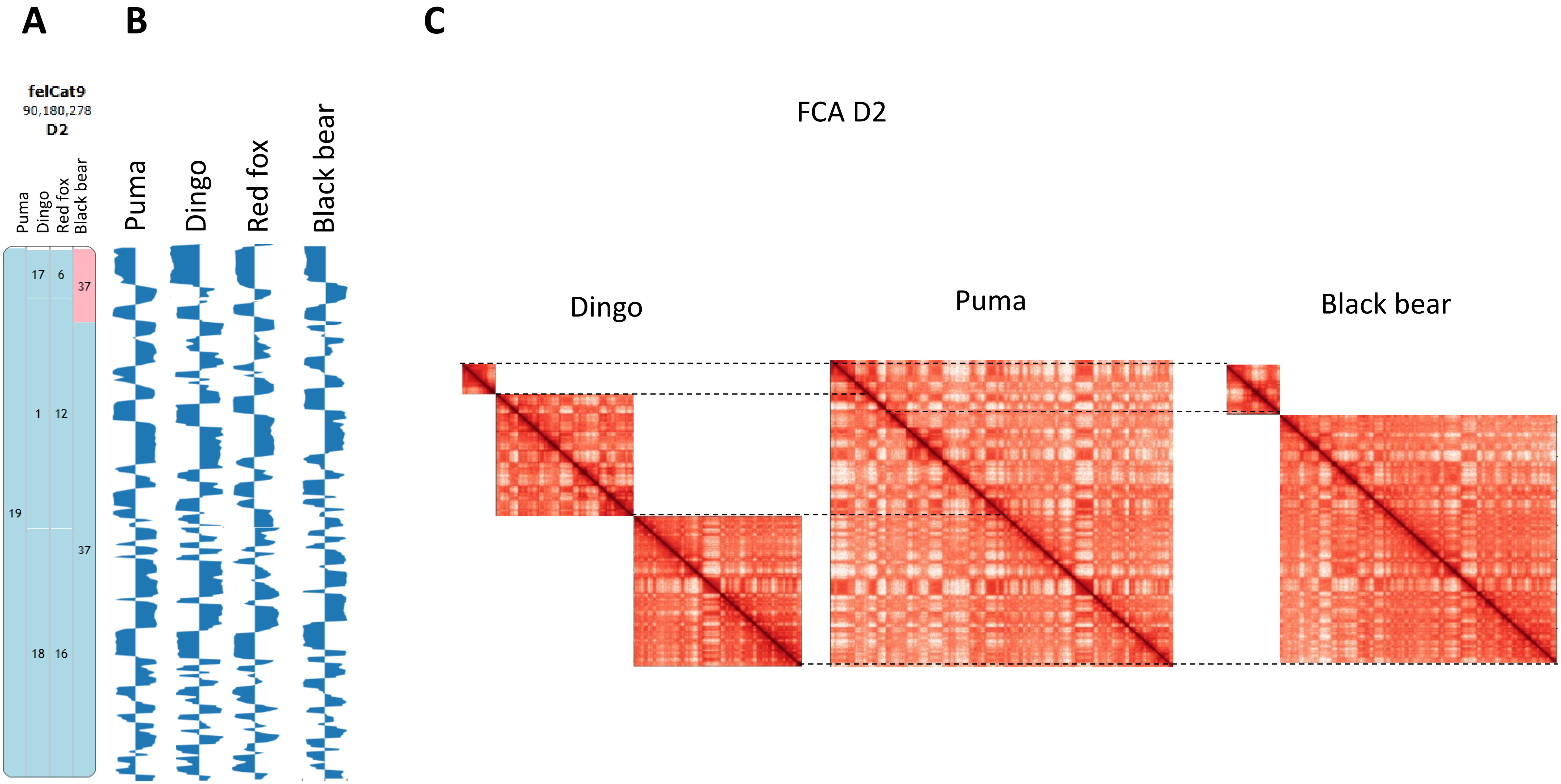
A
B
C
felCat9
90,180,278
D2
Puma
Dingo
Red fox
Black bear
17
6
37
1
12
19
37
18
16
Puma
Dingo
Red fox
Black bear
FCA D2
Dingo
Puma
Black bear

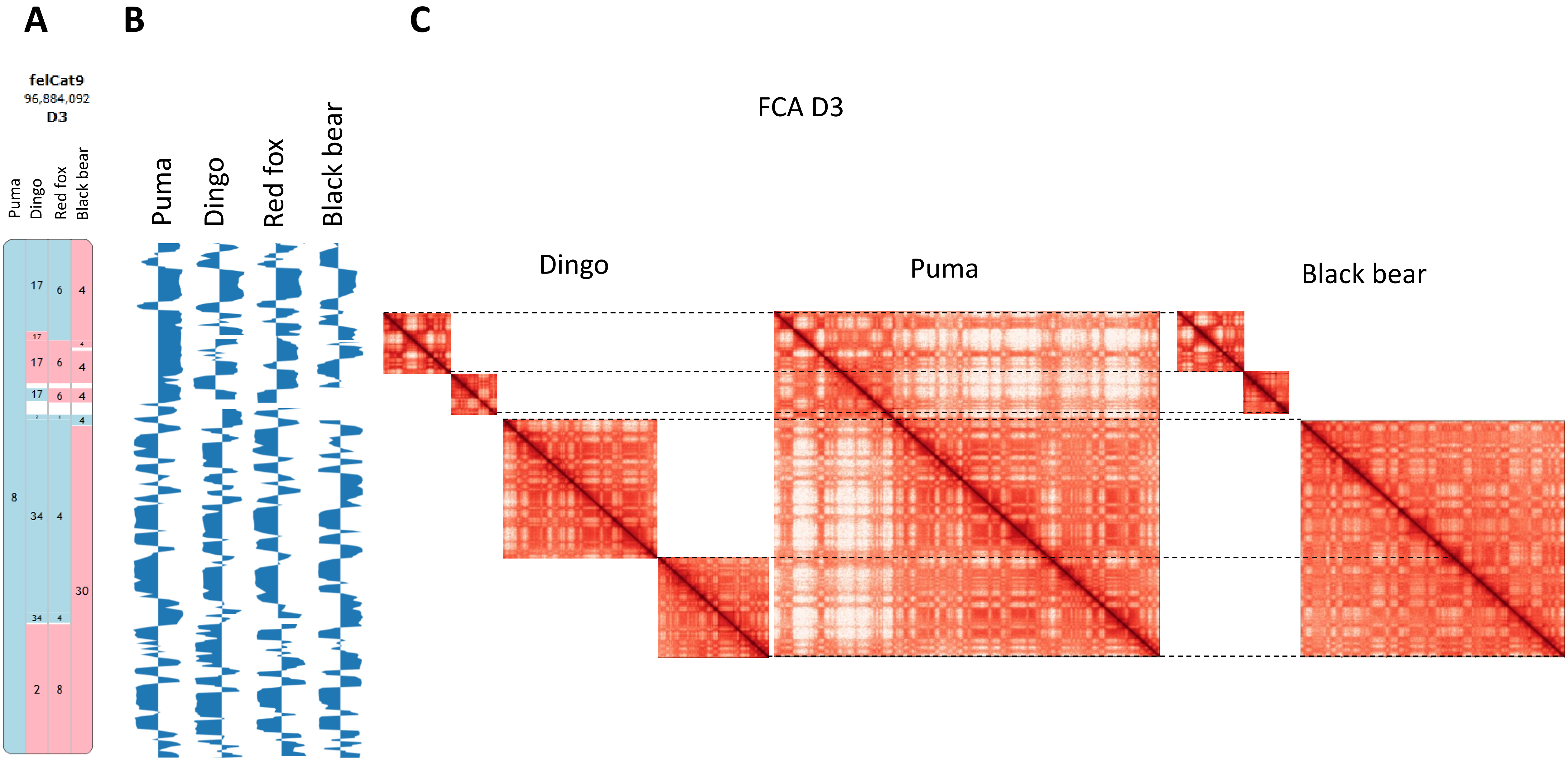
A
B
C
felCat9
96,884,092
D3
Puma
Dingo
Red fox
Black bear
FCA D3
Dingo
Puma
Black bear

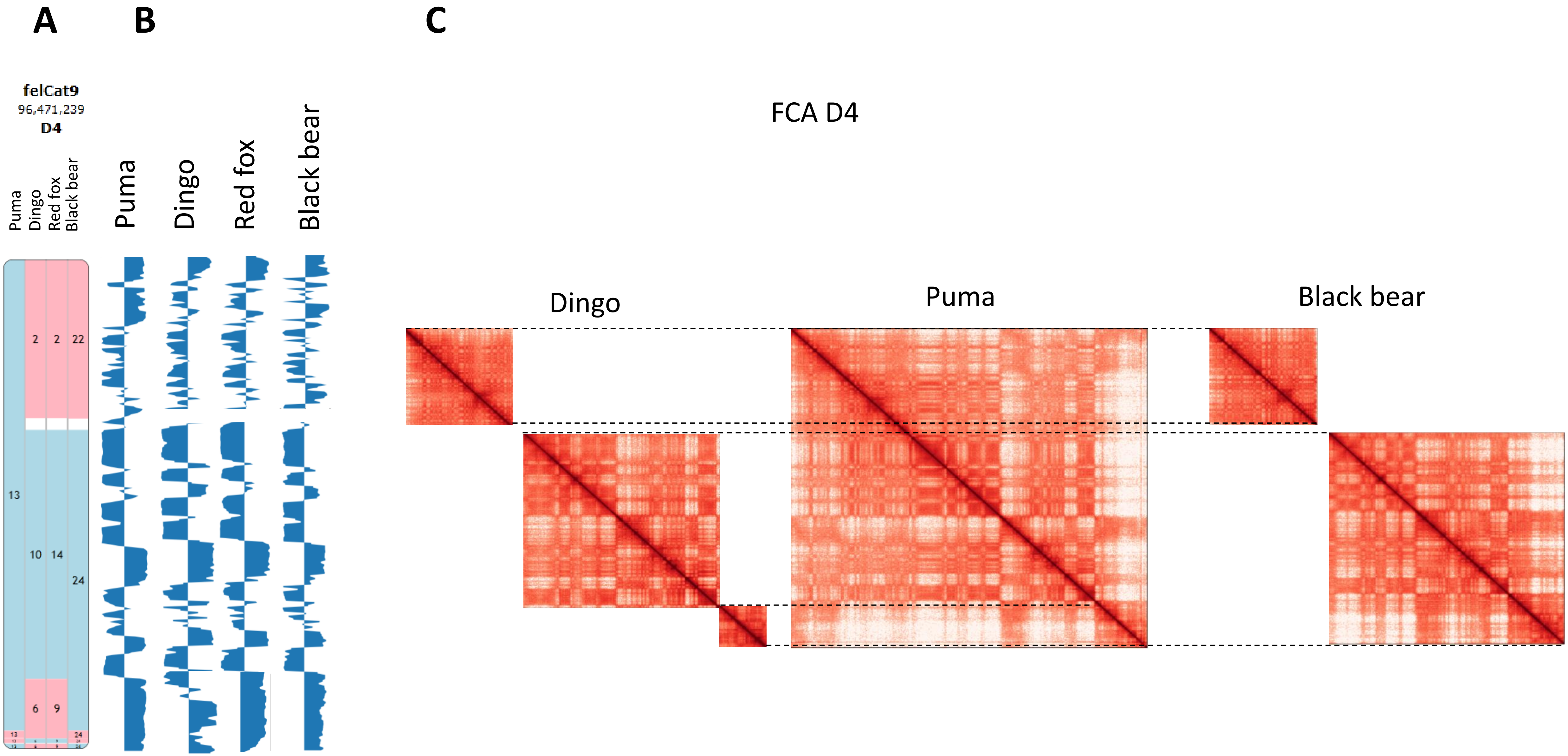
A
B
C
felCat9
96,471,239
D4
Puma
Dingo
Red fox
Black bear
2
2
22
13
10
14
24
6
9
Puma
Dingo
Red fox
Black bear
FCA D4
Dingo
Puma
Black bear

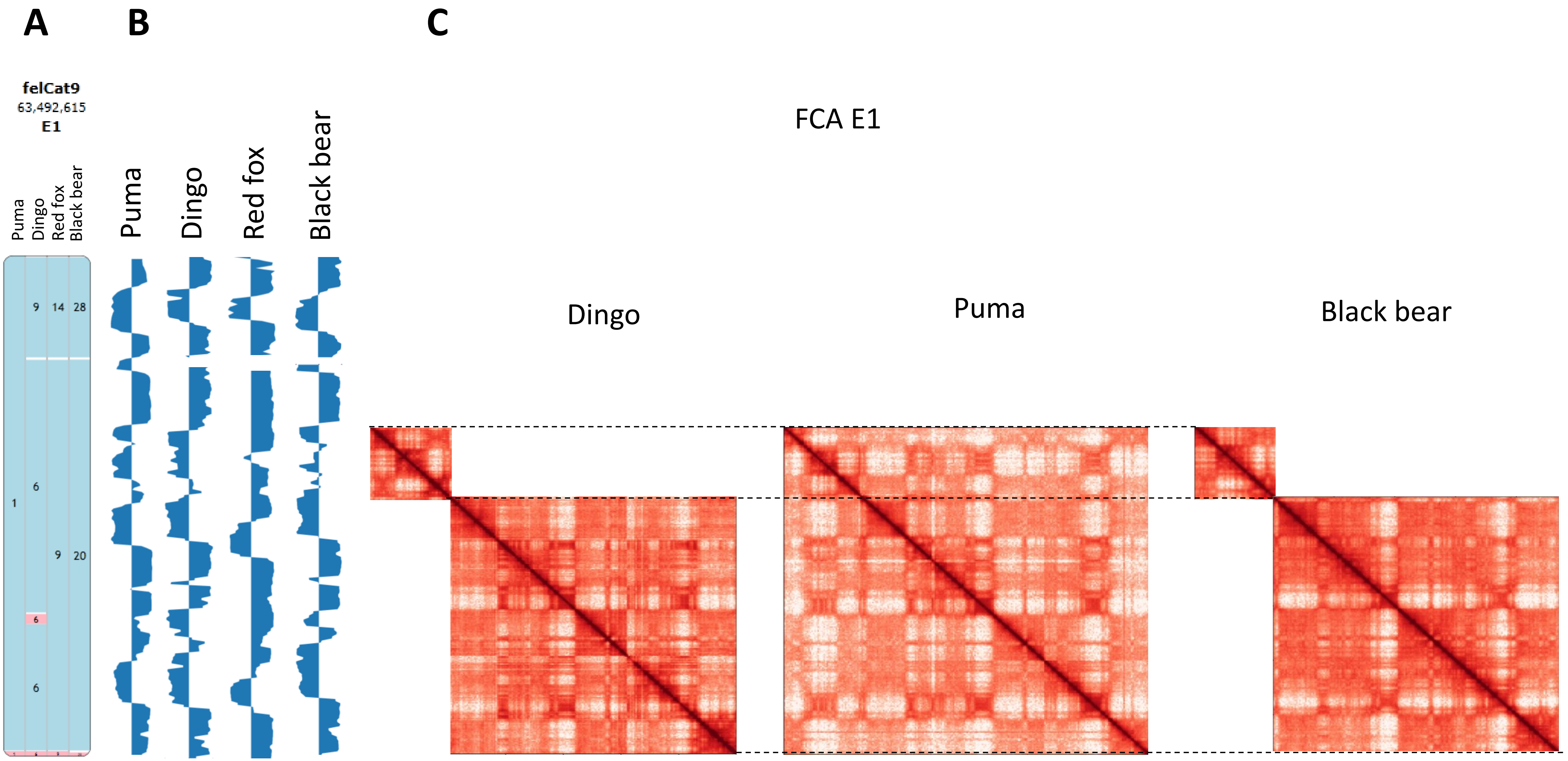
A
B
C
felCat9
63,492,615
E1
Puma
Dingo
Red fox
Black bear
9
14
28
1
6
9
20
6
6
FCA E1
Dingo
Puma
Black bear

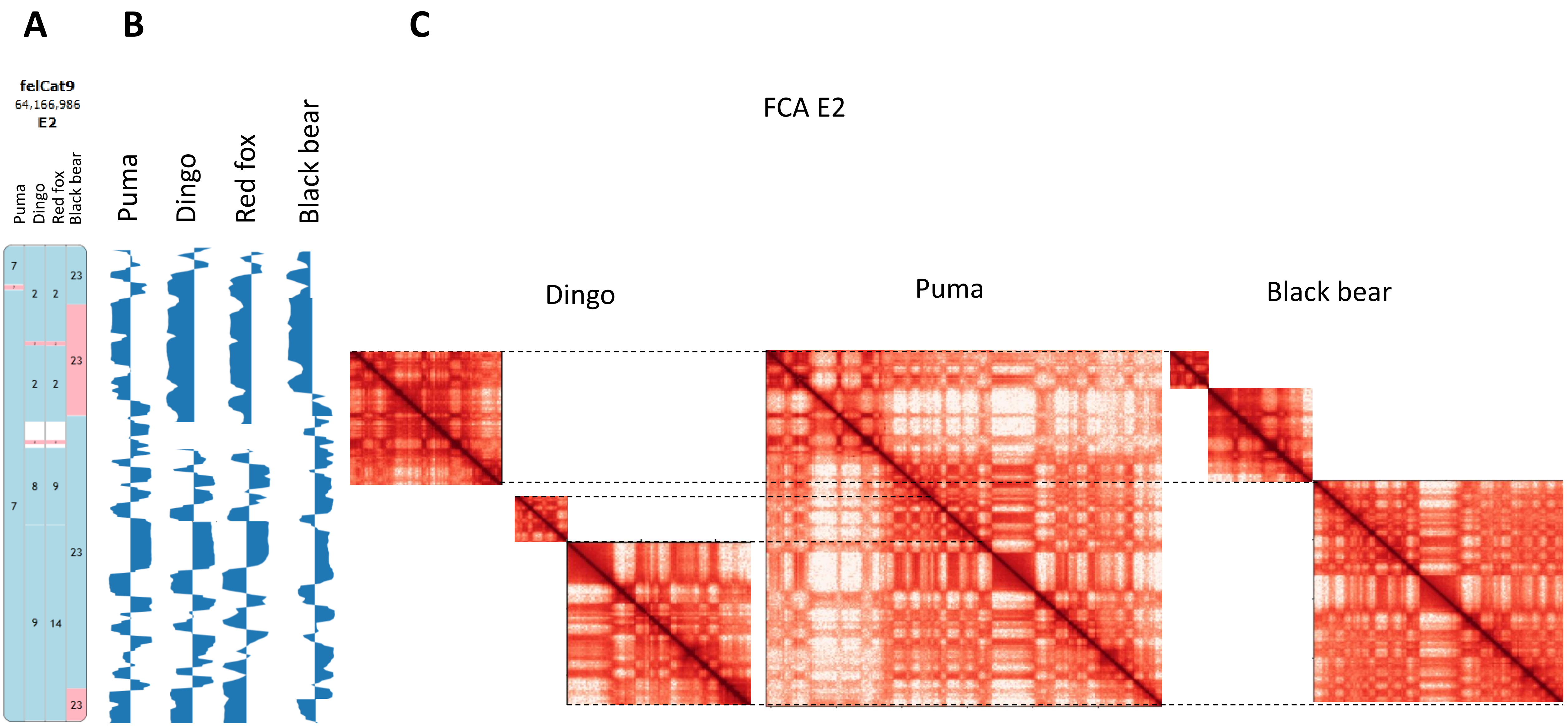

A
B
C
felCat9
64,166,986
E2
Puma
Dingo
Red fox
Black bear
FCA E2
Dingo
Puma
Black bear

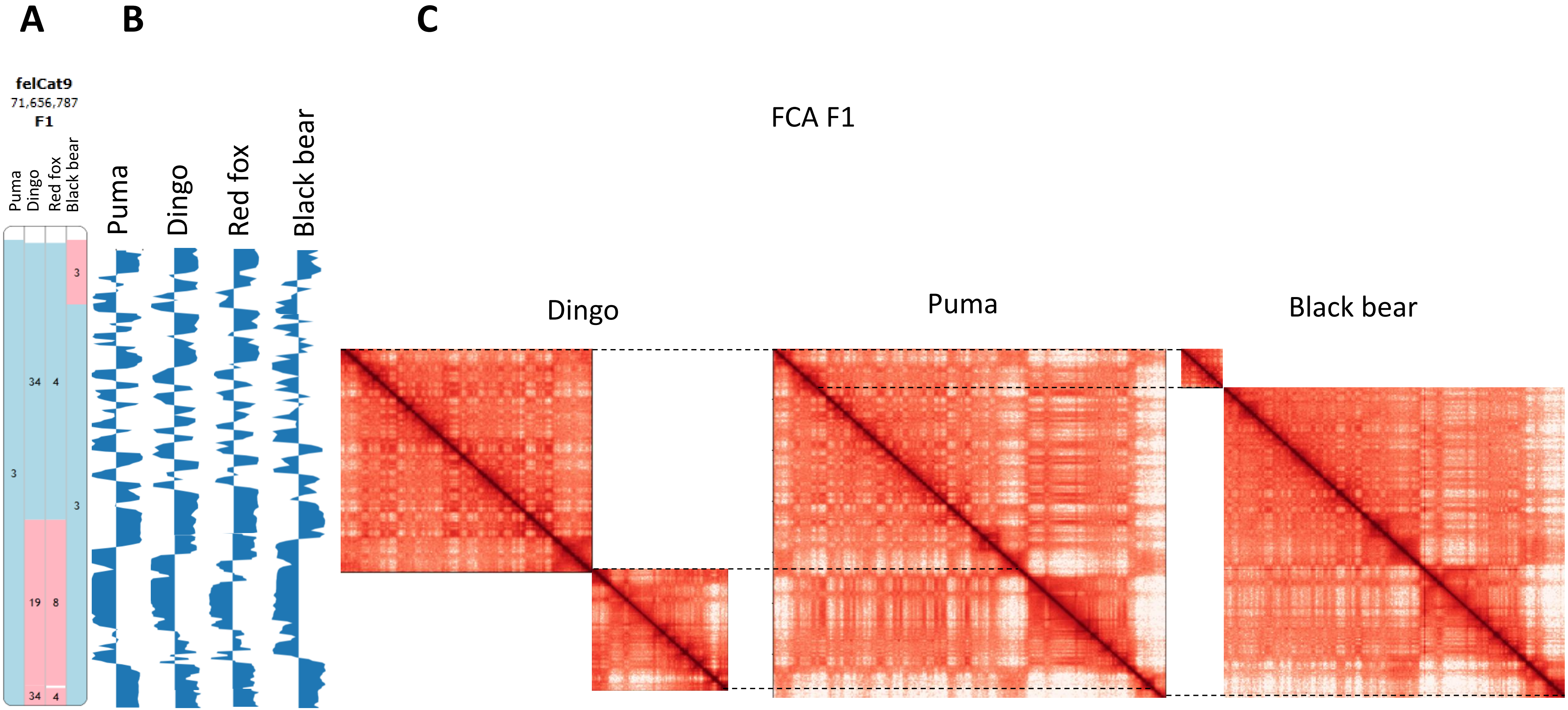
A
B
C
felCat9
71,656,787
F1
Puma
Dingo
Red fox
Black bear
3
34
4
3
19
8
34
4
3
3
Puma
Dingo
Red fox
Black bear
FCA F1
Dingo
Puma
Black bear

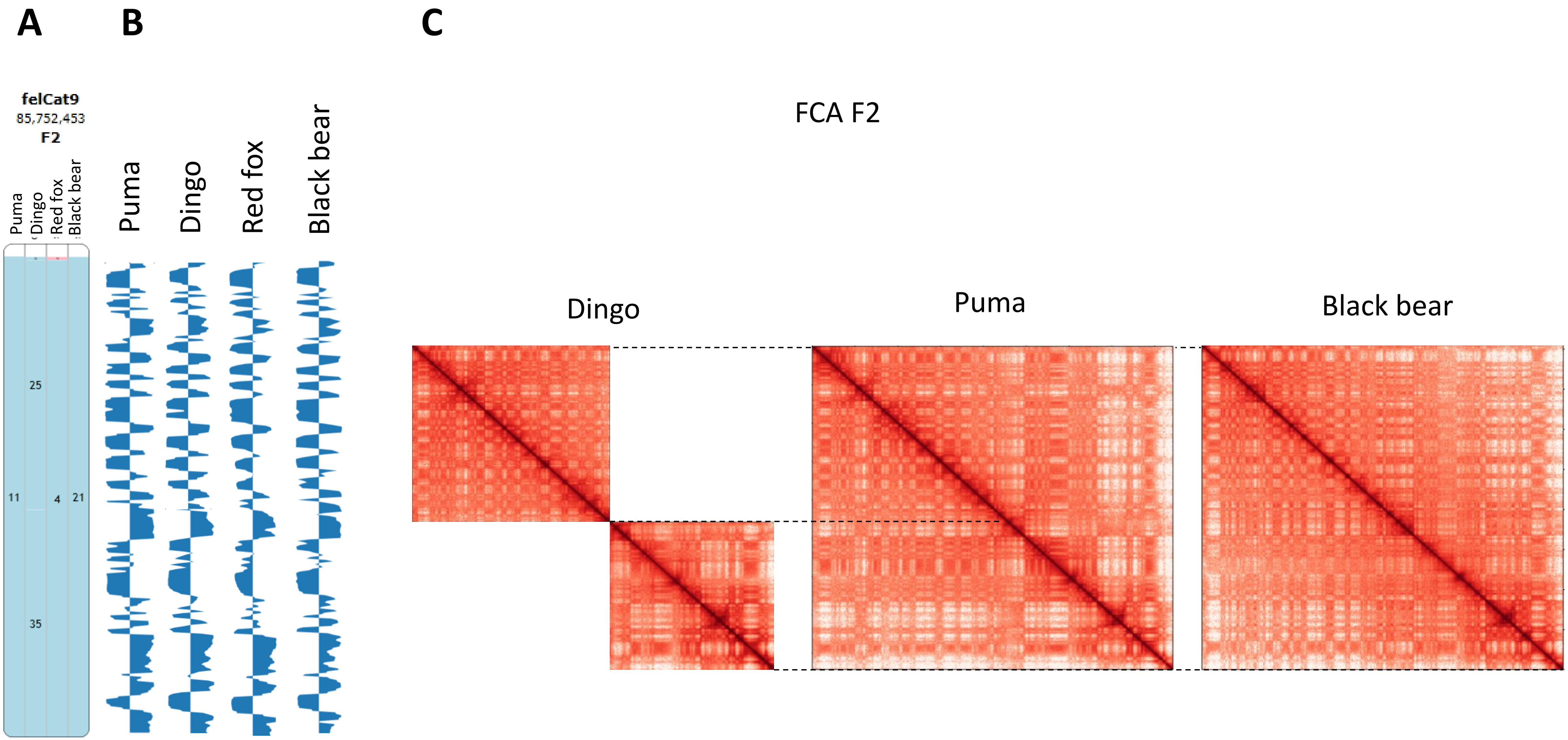

A
B
C
felCat9
85,752,453
F2
Puma
Dingo
Red fox
Black bear
25
11
4
21
35
Puma
Dingo
Red fox
Black bear
FCA F2
Dingo
Puma
Black bear

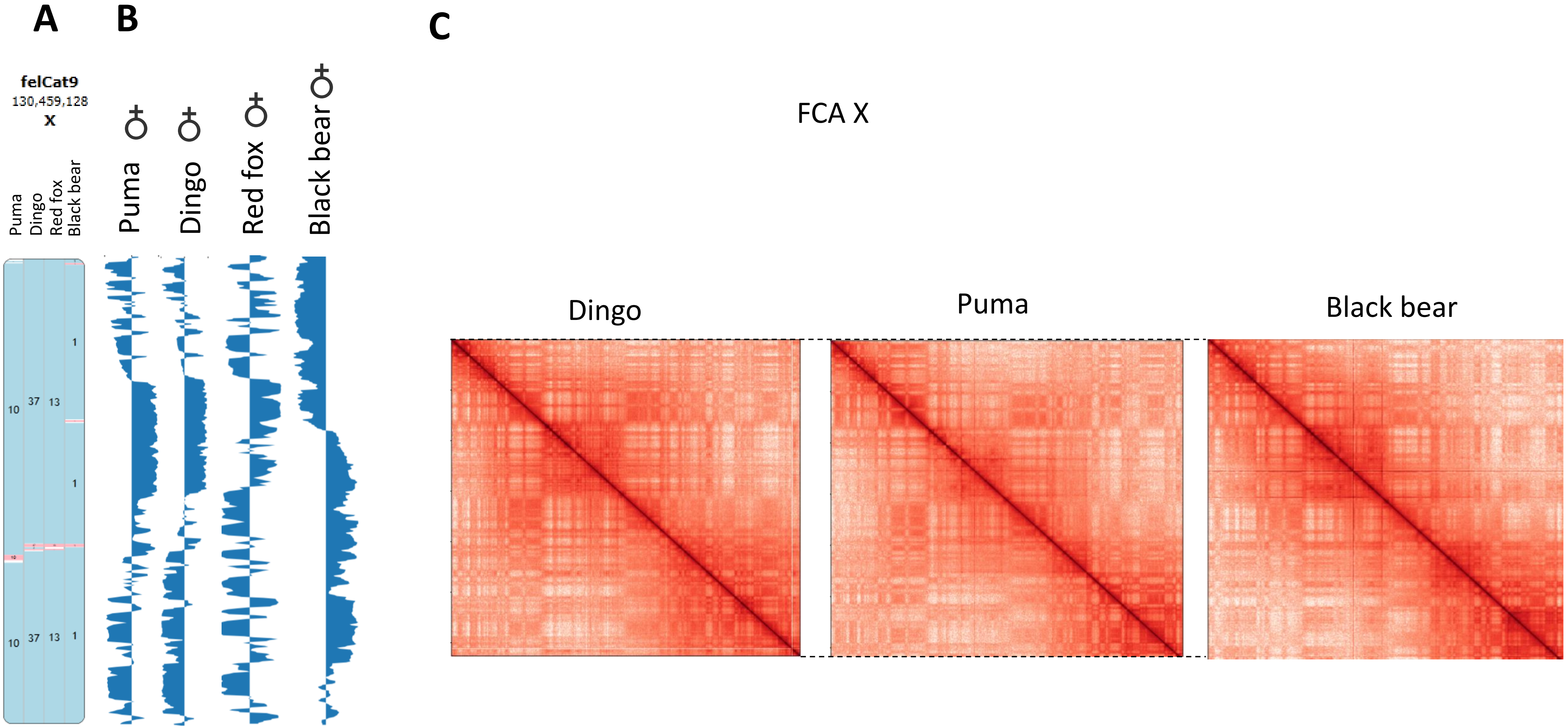
A
B
C
felCat9
130,459,128
X
Puma
Dingo
Red fox
Black bear
FCA X
Dingo
Puma
Black bear

Figure S6. Comparative 3D chromatin conformation analysis in carnivores.

(A) Alignments of representative species of felids, ursids, and canids (puma, dingo, red fox and black bear) C-scaffolds to cat chromosomes. Blue indicates the homologous synteny blocks in the same sequence orientation and numbers indicate C-scaffold identifiers. (B) Eigenvector values of each species aligned to the cat reference genome at 500 kb resolution. (C) Comparative analysis of Hi-C maps of the C-scaffolds. Color intensity reflects the frequency of interactions between pairs of loci on the C-scaffolds (range 1-1,000 for each map). Alignment coordinates can be found in SI Dataset S3.

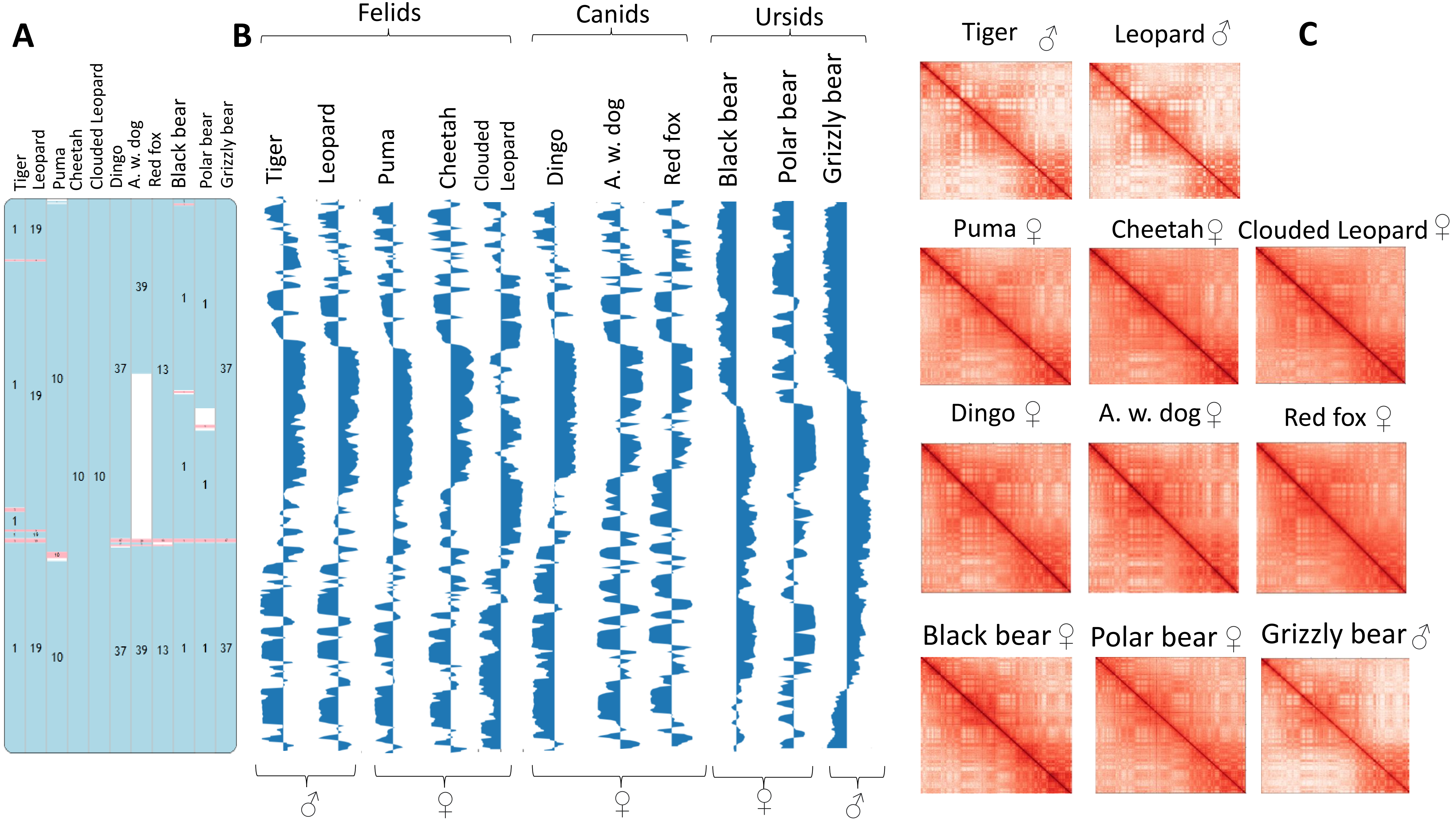

A
Tiger
Leopard
Puma
Cheetah
Clouded Leopard
Dingo
A. w. dog
Red fox
Black bear
Polar bear
Grizzly bear
1 19 39 1 1 1 19 10 37 13 37 1 1 10 10 1 1 19 10 37 39 13 1 1 37
B
Felids
Canids
Ursids
Tiger
Leopard
Puma
Cheetah
Clouded Leopard
Dingo
A. w. dog
Red fox
Black bear
Polar bear
Grizzly bear
♂ ♀ ♀ ♀ ♂
C
Tiger ♂
Leopard ♂
Puma ♀
Cheetah ♀
Clouded Leopard ♀
Dingo ♀
A. w. dog ♀
Red fox ♀
Black bear ♀
Polar bear ♀
Grizzly bear ♂

Figure S7 Comparative chromatin conformation analysis of the X chromosome.

(A) Alignments of felid, ursid, and canid C-scaffolds orthologous to FCA X. Blue indicates the homologous synteny blocks in the same sequence orientation and numbers indicate C-scaffold identifiers. (B) Eigenvector values of each species aligned to FCA X at 500 kb resolution. (C) Comparative analysis of X chromosome Hi-C maps of all species. Color intensity reflects the frequency of interactions between pairs of loci on the C-scaffolds (range 1-1,000 for each map). Black lines in puma, cheetah and clouded leopard Hi-C maps indicate boundaries of the two mega-domains. Alignment coordinates can be found in SI Dataset S3.

Dataset S1. Assemblies and genome statistics.

Dataset S2. Chromosome orthologies in felids and numbers of rearrangements.

Dataset S3. LastZ alignments of all species to the cat genome.

Dataset S4. Chromosome orthologies in canids and numbers of rearrangements.

Dataset S5. LastZ alignments of all canids to the red fox genome.

Dataset S6. Chromosome orthologies in ursids and numbers of rearrangements.

Dataset S7. LastZ alignments of all ursids to the black bear genome.

REFERENCES

1. A. Rhie, *et al.*, Towards complete and error-free genome assemblies of all vertebrate species. *Nature* **592**, 737–746 (2021).

2. H. A. Lewin, J. A. M. Graves, O. A. Ryder, A. S. Graphodatsky, S. J. O'Brien, Precision nomenclature for the new genomics. *GigaScience* **8** (2019).

3. J. Kim, *et al.*, Reconstruction and evolutionary history of eutherian chromosomes. *Proc. Natl. Acad. Sci. U.S.A.* **114**, E5379–E5388 (2017).

4. S. B. Hedges, J. Dudley, S. Kumar, TimeTree: a public knowledge-base of divergence times among organisms. *Bioinform* **22**, 2971–2 (2006).

5. B. C. Stöver, K. F. Müller, TreeGraph 2: Combining and visualizing evidence from different phylogenetic analyses. *BMC Bioinform* **11**, 7 (2010).

6. C. Román-Palacios, C. A. Medina, S. H. Zhan, M. S. Barker, Animal chromosome counts reveal a similar range of chromosome numbers but with less polyploidy in animals compared to flowering plants. *J Evol Biol* **34**, 1333–1339 (2021).

7. A. I. Makunin, *et al.*, Sequencing of Supernumerary Chromosomes of Red Fox and Raccoon Dog Confirms a Non-Random Gene Acquisition by B Chromosomes. *Genes* **9**, 405 (2018).
